# Profilin promotes lamellipodium protrusion by tuning the antagonistic activities of capping protein and VASP

**DOI:** 10.1038/s41467-026-77694-8

**Published:** 2026-09-16

**Authors:** Yubo Tang, Matthias Schaks, Xinqi Jiang, Marius Karger, Magdalena Mietkowska, Julian Kaltenhäuser, Jonas Scholz, Ruth Benavente-Naranjo, Sijian Hu, Sarah Körber, Zhilun Li, Christopher Lambert, Jessica L. Henty-Ridilla, Theresia E. B. Stradal, Roger Karlsson, Peter Bieling, Jan Faix, Martin Falcke, Klemens Rottner

**Affiliations:** 1 Division of Molecular Cell Biology, Institute for Cell- and Neurobiology, Technische Universität Braunschweig, Braunschweig, Germany; 2 Molecular Cell Biology, Helmholtz Centre for Infection Research, Braunschweig, Germany; 3 Institute for Biophysical Chemistry, Hannover Medical School, Hannover, Germany; 4 Department of Molecular Biosciences, The Wenner-Gren Institute, Stockholm University, Stockholm, Sweden; 5 Department of Microbial Drugs, Helmholtz Centre for Infection Research (HZI), Braunschweig, Germany; 6 Department of Biochemistry and Molecular Biology, SUNY Upstate Medical University, Syracuse, NY USA; 7 Department of Neuroscience and Physiology, SUNY Upstate Medical University, Syracuse, NY USA; 8 Department of Cell Biology, Helmholtz Centre for Infection Research, Braunschweig, Germany; 9 Department of Systemic Cell Biology, Max Planck Institute of Molecular Physiology, Dortmund, Germany; 10 Cellular Biophysics Section, Randall Centre for Cell and Molecular Biophysics, King’s College London, London, UK; 11 Max Delbrück Center for Molecular Medicine in the Helmholtz Association, Berlin, Germany; 12 Department of Physics, Humboldt University, Berlin, Germany; 13 Braunschweig Integrated Centre of Systems Biology (BRICS), Braunschweig, Germany; 14 Present Address: Division of Life Sciences and Medicine, University of Science and Technology of China, Hefei, China; 15 Present Address: Soilytix GmbH, Hamburg, Germany; 16 Present Address: Nanoscale Infection Biology, Helmholtz Center for Infection Research HZI, Braunschweig, Germany; 17 Present Address: Faculty of Biological and Environmental Sciences and HiLIFE Institute of Biotechnology, University of Helsinki, Helsinki, Finland; 18 Present Address: Westerdijk Fungal Biodiversity Institute, Utrecht, The Netherlands

**Keywords:** Lamellipodia, Actin, Filopodia, Applied mathematics, CRISPR-Cas9 genome editing

## Abstract

Cell migration frequently employs protrusions termed lamellipodia, constituting the prime model system for generation of branched actin filament networks. Here we utilize genome editing to explore the functional connections between the actin monomer-binding protein profilin (Pfn), the filament nucleating Arp2/3 complex, its co-factor heterodimeric capping protein (CP) and Ena/VASP family polymerases in lamellipodial actin assembly. We show that Pfn counters Ena/VASP but promotes Arp2/3 complex activity, while Ena/VASP and CP mutually antagonize each other. While Pfn promotes Arp2/3 complex activity irrespective of Ena/VASP, sensitivity of CP to Pfn removal vanishes in the absence of Ena/VASP. Our findings establish Pfn as master regulator of Arp2/3 complex-dependent actin network formation, differentially regulating VASP and its antagonizer CP. Mathematical modeling of our data suggest Ena/VASP and CP to compete for recruitment to lamellipodial edges. Our work provides critical insights into the molecular logic of branched actin network assembly in protrusion and force generation.

## Abstract (web summary)

Genetic disruption combined with mathematical modeling establish the actin monomer binding protein profilin as promoter of Arp2/3 complex-dependent actin network formation and tuner of the antagonistic activities of the growing actin filament end regulators VASP and capping protein.

## Introduction

Actin filament assembly is essential for numerous cellular processes such as shape establishment and change, division, organelle trafficking as well as cell-cell and cell- environment interactions. Actin filaments are continuously assembled from ATP-bound monomers, which are tightly regulated by conserved monomer-binding proteins. However, most research in past decades has focused on factors promoting filament nucleation^1–3^. Cells construct diverse actin structures depending on type, physiological state as well as tissue context. Actin filaments in these structures constantly polymerize and depolymerize, but the rate of turnover differs depending not only on network type, but also cell type and function. For example, actin networks at the leading edge of moving cells are more dynamic than those found in the interior of static cells, which feature large, rigid structures such as stress fibers anchored at focal adhesions^4,5^.

One of the most dynamic actin structures is the lamellipodium, crucial for cell migration and axonal growth^6–8^. It serves as key model system for studying rapid actin remodeling, due to its consistent features, such as a network density gradient from front to back and a uniform criss-cross arrangement of branched actin filaments. Arp2/3 complex activation at the lamellipodium front generates a dense array of branched filaments that create pushing forces, which drive cell protrusion. The WAVE Regulatory Complex (WRC), activated by the Rho-family GTPase Rac (1, 2 or 3), is a major activator of Arp2/3 complex, leading to branching at the lamellipodium front^9,10^. Despite the central role of the WH2-, central and acidic motifs (WCA) in autoinhibitory interactions of the WRC and the activation of the Arp2/3 complex^11^, recent data suggest that the Arp2/3-activating activity of the WCA domain can be replaced by alternative pathways^12^. Aside from continuous branching at the lamellipodium front, the interruption of which leads to lamellipodium collapse^13^, a limited number of additional activities are needed in order to maintain a balance between actin filament assembly and disassembly within the lamellipodium. These include actin filament polymerases such as of the Ena/VASP or formin classes of proteins^14,15^, filament capping proteins such as CP^16^ and actin disassembly factors such as cofilin/actin depolymerizing factor (ADF) family members^17^. The latter are essential for actin network turnover^18,19^, whereas CP limits filament elongation and thereby length and also indirectly promotes Arp2/3-dependent branching^20,21^.

Profilin (Pfn), an actin monomer-binding protein, plays a critical, yet underexplored, role in this process. Of the four Pfn genes present in the mammalian genome, Pfn1 is most abundant and ubiquitous, whereas −2 (specifically −2a) is enriched in the nervous system and Pfn3 and −4 are testis-specific^22^. Pfn inhibits spontaneous, uncontrolled actin filament nucleation by forming a stoichiometric complex with actin monomers and promotes nucleotide exchange, thereby generating the main source of polymerizable actin available for elongation of the fast-growing, so called barbed ends of actin filaments. It also interacts with formins to accelerate actin elongation^23–27^. Though less dependent on Pfn than formins in vitro^28–30^, Ena/VASP proteins also interact with profilin:actin, but their relationship with Pfn in vivo remains unclear^14^.

Conflicting studies have suggested that Pfn either promotes or inhibits Arp2/3 complex activity. Seminal work in fission yeast showed that Pfn favors the assembly of formin- over Arp2/3-dependent networks^31^. Work in mammalian cells described that Pfn overexpression inhibits lamellipodia by impeding Arp2/3 complex activity and localization at the leading edge^32^. Both studies thus suggested that Pfn funnels polymerizable actin into formin- or Ena/VASP-dependent structures at the expense of Arp2/3 complex-generated networks^33^. However, a more recent study genetically disrupting Pfn1 expression in the neuronal CAD cell line came to opposite conclusions concerning the relation between Pfn and Arp2/3 complex functions^34^.

To resolve these conflicts, we here investigate the impact of Pfn disruption on lamellipodia formation in B16-F1 melanoma cells, a well-established model for studying actin protrusions^35–37^. We examine Pfn’s effects on Arp2/3 complex activity and Ena/VASP distribution and the interconnections between Pfn, Ena/VASP, and CP within lamellipodia. Finally, mathematical modeling of Pfn-deficient and combined knockouts suggests that VASP and CP compete for binding sites at the lamellipodium front, contributing to their functional antagonism in actin network regulation.

## Results

### Profilin suppression differentially affects lamellipodia *versus* filopodia formation and formin function

**Fig. 1:**
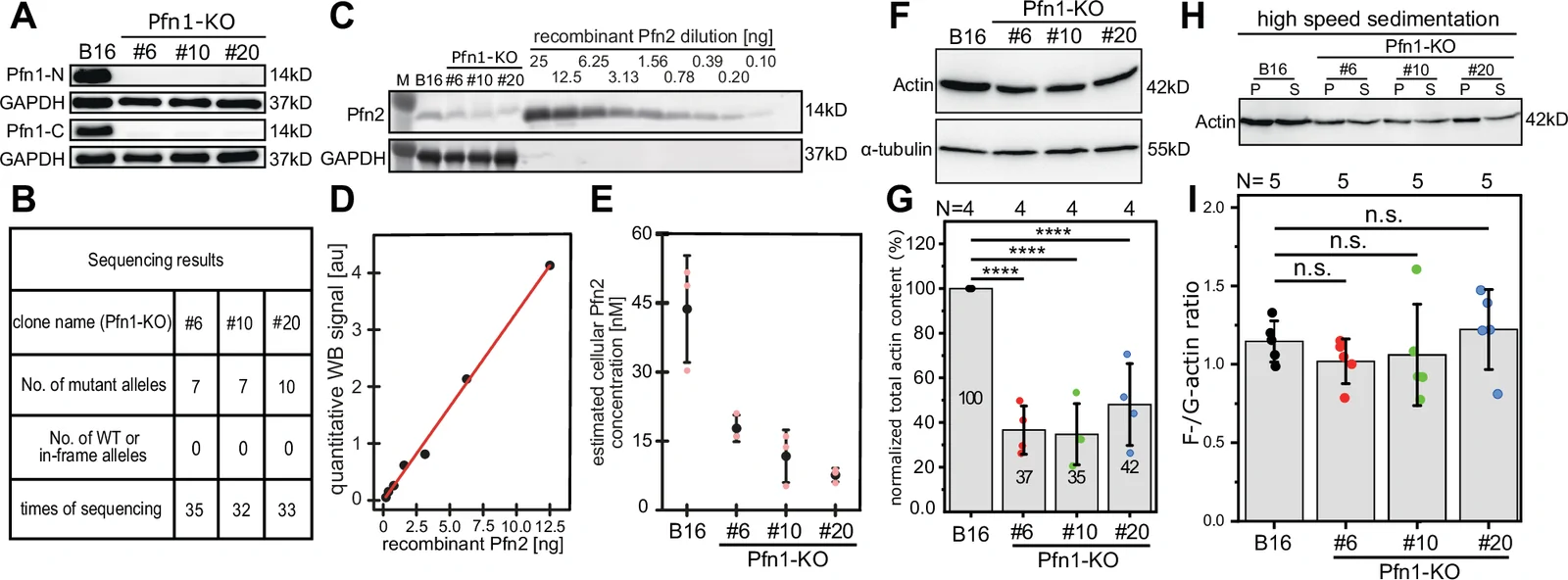
Generation and initial characterization of profilin 1 knockout (Pfn1-KO) cell lines. **A** Western blotting of B16-F1 cell lines before (B16) and after clonal isolation of cells treated with a Pfn1-specific guide construct, using two independent antibodies specific for the N- and C-terminus of profilin and GAPDH as loading control. **B** Summary of the sequencing results of the genomic locus encoding the Pfn1 gene in each cell line, as indicated. No WT or in-frame deletion was detected in at least 32 independent sequencing reactions per cell line. **C** Representative Western blot of whole-cell extracts of Pfn1-KO clones and B16-F1 cells next to a serial dilution of recombinant human profilin 2 (Pfn2). GAPDH was used to normalize for unequal sample loading. **D** Example standard curve that relates the immunoblot signal to the known quantity of recombinant Pfn2. The line is a linear fit to the data. **E** Calculated cellular concentrations of Pfn2 for WT (B16) and Pfn1-KO clones. Data from three independent experiments (pink, small symbols) were averaged to obtain mean (large, black symbols) concentrations. Error bars are standard deviations of means (SD). **F** Comparison of actin and tubulin expression in WT *versus* Pfn1-KO cell lines, as indicated. **G** Actin expression as normalized to α-tubulin and 100% in WT B16-F1 cells. Note the significant reduction of actin expression (to app. 40% on average) as compared to B16-F1 control. Data are arithmetic means and standard deviations (error bars) from four independent experiments (N). One-way ANOVA and Tukey Multiple Comparison test were used to reveal statistically significant differences between datasets. ****p ≤ 0.001. **H** Representative Western blot using actin antibodies of supernatant (S) and pellet (P) fractions of respective cell lysates upon high speed sedimentation. **I** Quantification of the Western blotting data in (**H**), displayed as F- over G-actin ratio (P/S-fractions) for each cell line, as indicated. Data are arithmetic means and standard deviations (error bars) from five independent experiments (N). One-way ANOVA and Tukey Multiple Comparison test were used to reveal statistically significant differences between datasets. n.s., not significant.

Pfn1 has been implicated in the formation of both lamellipodia and filopodia, for instance in the brain-tumor-derived CAD (Cath.-a-differentiated) cell line, where it is expressed at ~10-fold higher levels than Pfn2^34^. Surprisingly, Pfn1 knockout in these cells was described to cause loss of Arp2/3 complex at the cell periphery—eliminating canonical lamellipodia—and a marked reduction in linear actin filament arrays, likely corresponding to microspike- or filopodia-like bundles. This is unexpected, given prior suggestions of an antagonistic relationship between profilin and Arp2/3 complex^31,32^. To probe the relevance of Pfn1 and overall profilin activity for lamellipodia *versus* filopodia formation, we first disrupted the *Pfn1* gene in B16-F1 melanoma cells using CRISPR/Cas9. Three independent Pfn1 knockout clones (termed Pfn1-KO #6, #10, #20) were identified by Western blotting and locus sequencing (Fig. 1A, B). To assess the contribution of Pfn2 in the absence of Pfn1, we quantified Pfn2 expression in these lines (Fig. 1C–E). Unexpectedly, Pfn2 levels were not upregulated; instead, they were even lower than in WT cells, ranging from ~7 to 22 nM depending on mutant cell line. Based on a previously reported Pfn1 concentration of 66 µM in the related B16-F10 line^38^, this indicates that Pfn2 is expressed at levels 4–5 orders of magnitude lower than Pfn1 in WT cells. This scarcity is consistent with the virtual elimination of mDia2 formin-dependent filopodia, in spite of retained peripheral localization of this formin (Supplementary Movie 1). A similar loss was observed for filopodia induced by active variants of FMNL3 (Supplementary Fig. 1) and in conditions favoring spontaneous filopodia formation (Supplementary Fig. 2). More specifically, while lamellipodia formation frequency remained unchanged in Pfn1-KO cells compared to WT (Supplementary Fig. 2A, B), filopodia were strongly suppressed in all conditions, even if enhancing spontaneous filopodia by combining low laminin (2.5 ng/ml) and serum concentration (1%) (Supplementary Fig. 2C, D). These findings indicate that our Pfn1-KO lines exhibit severely impaired formin-dependent actin filament elongation^23^, potentially approximating pan-formin knockouts in this respect.

### Pfn elimination is lethal and its suppression reduces actin expression without altering the F-/G-actin ratio

Next, we attempted to fully abolish profilin activity by targeting Pfn2 in Pfn1-KO clone #20 or disrupting Pfn1 in a stable Pfn2-KO line (clone #41). Although we achieved near-complete, transient elimination of the remaining profilin in both cases, we were unable to obtain any stably growing double knockout clones (Supplementary Fig. 3A–E). Similarly, in *Dictyostelium*, which expresses three functional profilins^39^, stable elimination of Pfn I and II is possible^40^, but attempts to additionally disrupt Pfn III failed (Supplementary Fig. 4A, B), despite it being non-essential in a WT background (Supplementary Fig. 4B, left panel)^39^. These findings suggest that complete loss of profilin activity is lethal to cells, at least in the Amorphea clade, likely due to essential roles in processes such as cell division^41–43^. In line with this, we observed an increased fraction of multinucleated nuclei in our Pfn1-KO lines, inversely correlated with residual Pfn2 expression (Fig. 1E and Supplementary Fig. 5).

We then examined actin expression in Pfn1-deficient cells and found it reduced to app. 40% of WT levels (Fig. 1F, G). This decrease, however, did not alter the F-/G-actin ratio—the proportion of actin incorporated into filaments *versus* monomers (Fig. 1H, I). Thus, despite severely impaired formin-dependent filament elongation, the relative extent of actin filament assembly remained unchanged. While this contrasts with findings in CAD neuronal cells^34^, it aligns with only moderately altered migratory capability (Supplementary Movies 2 and 3) or formation of major actin structures—including lamellipodia and stress fibers—in our Pfn1-deficient lines (Supplementary Fig. 2).

### Pfn1 loss changes lamellipodial morphology and dynamics, reducing protrusion and migration

**Fig. 2:**
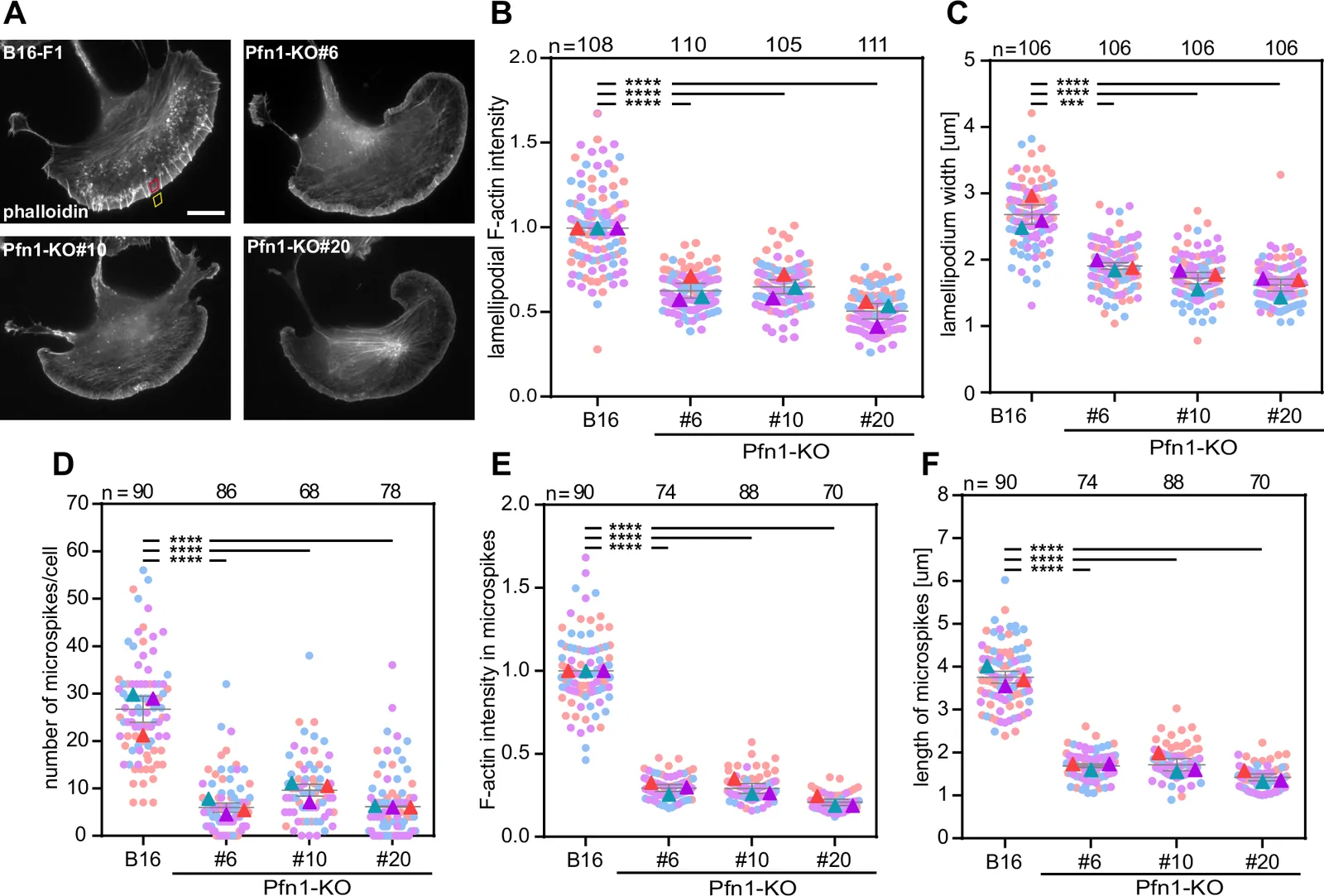
Characterization and quantification of lamellipodial features in Pfn1-KO cell lines. **A** Representative wildtype and Pfn1-deficient cells (derived from clones as indicated) growing on laminin and fixed and stained for the actin cytoskeleton using fluorescently-coupled phalloidin. The rhomb-shaped area in red marks an example region used to measure the lamellipodial actin network intensity as shown in B. Areas lacking microspike bundles were measured, as these were analyzed separately. For obtaining the data displayed in B, each area in red was subtracted by a close-by area outside the cell (yellow rhomb) representing extracellular background fluorescence. Scale bar in top left is valid for all panels: 10 µm. **B** Quantitation of lamellipodial actin filament (F-actin) intensities in cell lines as indicated and measured as illustrated in subpanel (**A**). The arithmetic means of fluorescence background-subtracted B16-F1 wildtype (B16) data were normalized to 1 for each of three independent experiments, and shown as so called superplots^81^, displaying measurements from individual cells (see “Methods”) and their arithmetic means as color-coded dots and triangles, respectively. Gray lines and error bars display means and standard errors of means (SEMs), *n* = numbers of measured cells. **C** Quantitation of lamellipodial widths, i.e., average orthogonal distances measured from the protruding edge toward the lamellipodial rear in the cell interior. Data assessed in principle and displayed as superplots as described in B, with the exception that absolute numbers (in µm) are shown. **D** Quantitation of microspike numbers embedded into lamellipodia per cell, and displayed as absolute numbers as described in (**C**). **E** Quantitation of F-actin intensities throughout microspikes, with data assessed and displayed analogous to (**B**). (**F**) Microspike lengths quantified, assessed and displayed as described for data in (**C**). For all superplot data in subpanels (**B**–**F**), arithmetic means of replicates from each individual KO-line were statistically compared to WT using ANOVA with Dunnett’s post hoc test; **p ≤ 0.01; ***p ≤ 0.005; ****p ≤ 0.001.

To explore profilin’s role as both potential antagonist of Arp2/3- and promoter of formin-mediated actin assembly^33^, we studied the morphology and dynamics of lamellipodia—the best-characterized Arp2/3-dependent structure^10^. We initially assessed lamellipodial behavior in all three Pfn1-KO lines using phase-contrast and fluorescence video microscopy of cells expressing EGFP-tagged actin. Given the proposed requirement for both Arp2/3- and Ena/VASP-mediated actin assembly at lamellipodial edges^34^, protrusion and dynamics of these structures appeared largely normal (Supplementary Movie 4). Phalloidin staining showed at best slightly altered lamellipodial actin networks, except for a striking reduction in embedded microspike bundles (Fig. 2A). However, quantitation revealed clear reductions of lamellipodial F-actin content and width (Fig. 2B, C), along with a dramatic loss of microspike numbers (Fig. 2D). The remaining microspikes were drastically shorter and dimmer (Fig. 2E, F), consistent with the overall reduction of lamellipodial width (Fig. 2C). These observations confirm the differential impact of profilin loss on actin structures—strongly affecting protrusive bundles such as filopodia and microspikes, while more modestly impairing Arp2/3-dependent networks. Notably, the morphological changes were accompanied by reduced lamellipodial protrusion rates (Supplementary Fig. 6A, B), slower network assembly (Supplementary Fig. 6C, D, Supplementary Movie 5) and a modest decline in cell migration (Supplementary Figs. 6E, 7, and Supplementary Movie 2).

### Pfn1 loss reduces but does not eliminate Arp2/3 complex incorporation at the cell periphery

**Fig. 3:**
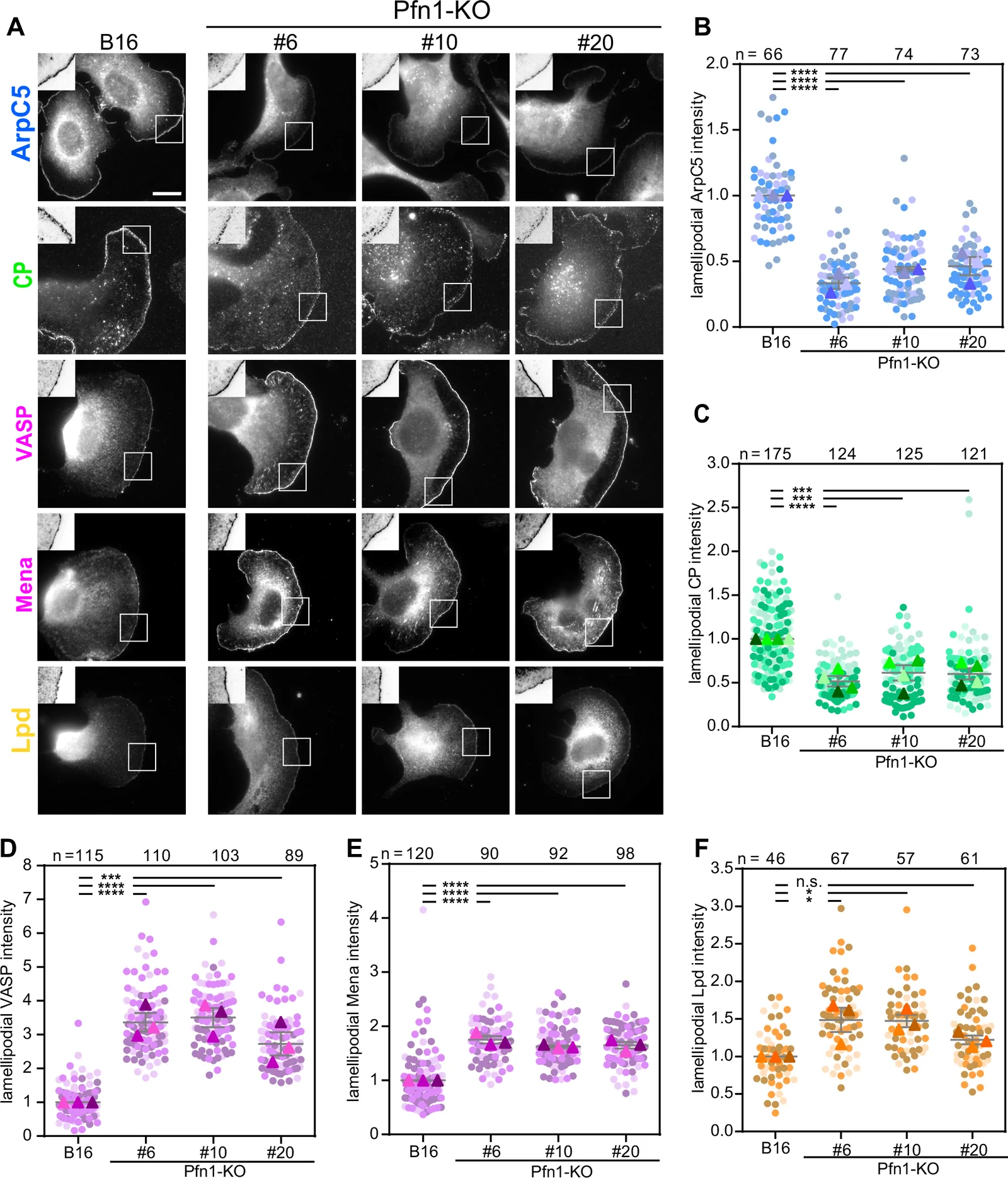
Consequences of profilin loss of function on lamellipodial accumulation of key actin assembly regulators. **A** Representative images of B16-F1 melanoma cells seeded on laminin and stained for Arp2/3 complex (ArpC5, blue), capping protein (CP, green), Ena/VASP family members (VASP and Mena, pink), lamellipodin (Lpd, yellow). White squares depict insets displayed as magnified and inverted images at top left corners of each individual panel. Note respective changes in lamellipodial intensities that can be appreciated as black lines in inverted insets for each lamellipodial regulator. **B**–**F** Quantitations of lamellipodial fluorescence intensities of respective components, color-coded as described in (**A**) and displayed as superplots with independent experiments shown in distinct shadings of respective colors. Gray lines and error bars display means and standard errors of means (SEMs). Intensities measured of all components were normalized to the arithmetic mean of the WT population for each individual experiment (colored rectangles), *n* = measured cells from at least three independent experiments (four in **C**). For all superplot data shown in (**B**–**F**), arithmetic means of replicates from each individual KO-line were statistically compared to WT using ANOVA with Dunnett’s post hoc test; n.s. not significant; *p ≤ 0.05; ***p ≤ 0.005; ****p ≤ 0.001.

We next assessed how the near complete loss of profilin activity, along with reduced lamellipodial network polymerization, affects the distribution of key actin assembly factors. Profilin has been proposed to bias actin monomer usage toward formin- over Arp2/3 complex-mediated assembly^44^, suggesting that in its absence, remaining monomers might preferentially fuel Arp2/3-dependent polymerization. Contrary to this model of functional antagonism and despite diminished formin activity, we observed a strong reduction in Arp2/3 complex incorporation into the lamellipodia of Pfn1-KO cells (Fig. 3A, B), to less than 50% of WT cells. This reduction exceeded the decline in total lamellipodial F-actin content (Fig. 2B). Similar results were obtained for the Arp2/3 complex agonist capping protein (CP, Fig. 3A, C), despite in vitro evidence suggesting competition between profilin and CP for barbed end binding^45^.

These findings contrast with a previous report in Pfn1-KO CAD cells, which described complete loss of Arp2/3 incorporation at the cell periphery^34^. However, that claim is inconsistent with both our prior studies on Arp2/3 complex subunit knockouts^46,47^ (see also below) and the remaining lamellipodial structures and features observed in Pfn1-deficient cells here. To confirm Arp2/3 complex involvement in lamellipodial protrusion of Pfn1-deficient cells, we conducted two complementary experiments. First, transient expression of EGFP-tagged ArpC5B in Pfn1-KO clone #20 revealed reduced but specific incorporation of the Arp2/3 complex into peripheral protrusions (Supplementary Movie 6). Second, we disrupted the essential Arp2 subunit (encoded by a single gene) in the Pfn1-KO background. As control, we initially disrupted the Arp2 gene in B16-F1 WT cells and found a complete elimination of lamellipodia as well as strong suppression of random migration in three independently analyzed clones (Supplementary Fig. 8A–D), as expected^46–49^. However, if the lamellipodia in Pfn1-KO cells were Arp2/3-independent—as proposed for CAD cells^34^—their structure should persist. Instead, the two independent clones of double-KO (DKO) cells analyzed (Pfn1+Arp2 DKO #15, #20) exhibited an actin cytoskeleton dominated by peripheral bundles at concave cell edges, again lacking any lamellipodia-like protrusive networks, unlike cells lacking Pfn1 alone (Supplementary Fig. 8E, F). With both formin- and Arp2/3 activities impaired, these cells showed a near-complete loss of migration compared to either WT or Pfn1-KO cells (Supplementary Fig. 8G, H, Supplementary Movie 7). In conclusion, although Arp2/3 complex activity is markedly reduced in Pfn1-deficient B16-F1 melanoma cells, their lamellipodia remain strictly dependent on its function.

### Pfn1 loss induces Ena/VASP accumulation, critical for lamellipodial protrusion and migration upon profilin suppression

Unlike Arp2/3 complex and CP, Ena/VASP family actin polymerases were strongly enriched at lamellipodial edges in Pfn1-deficient cells (Fig. 3A). This accumulation was observed in all three Pfn1-KO clones for both VASP (Fig. 3D) and Mena (Fig. 3E). Lamellipodin (Lpd), a prominent Ena/VASP interactor^50,51^, was also significantly increased at these sites in Pfn1-deficient cells, albeit with some variability between clones (Fig. 3A, F) and although previously established not to be essential for Ena/VASP targeting to lamellipodia^52,53^. VASP is known to accumulate at lamellipodial edges in proportion to protrusion rates^54,55^. However, in Pfn1-KO cells, VASP enrichment persisted despite reduced protrusion and actin polymerization. This uncoupling suggests a compensatory mechanism for reduced Arp2/3 activity (see above), potentially aided by CP downregulation. While a similar Ena/VASP accumulation was observed in Pfn1-KO CAD cells, those authors argued, by contrast, the assembly factors were non-functional without Pfn1 (see ref. ^34^). In addition, our earlier work showed that genetic Ena/VASP removal enhances Arp2/3 and CP accumulation in lamellipodia^56^, though the underlying mechanism remained unresolved.

**Fig. 4:**
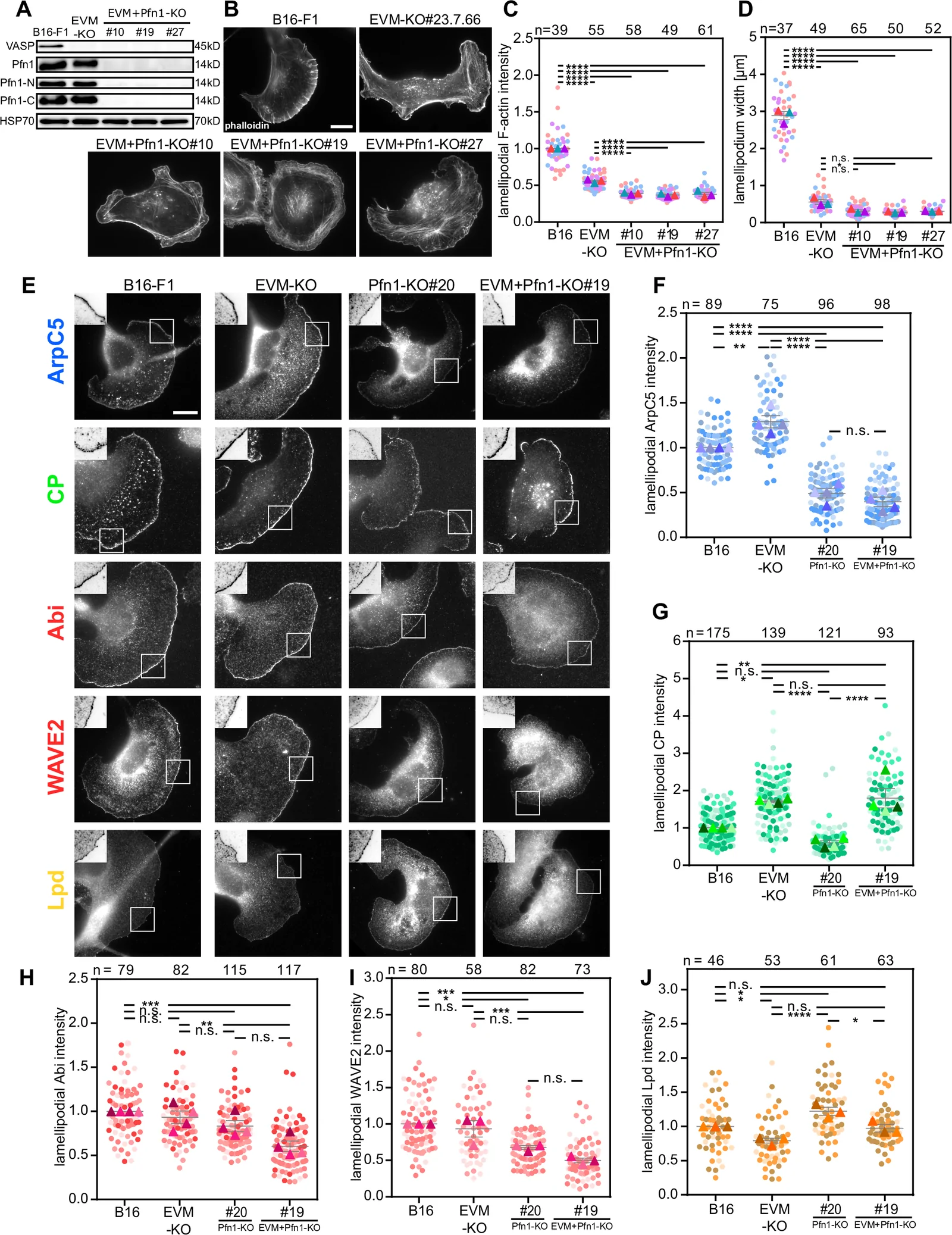
Profilin 1 removal in Ena/VASP-deficient cells compromises lamellipodia protrusion with simultaneous, differential impact on distinct core actin machinery components. **A** Western blotting of WT (B16-F1) as control and Ena/VASP-deficient cells before (EVM-KO) and after clonal isolation of cells treated with a Pfn1-specific guide construct. Quadruple-KO (EVM+Pfn1-KO) clones #10, #19 and #27 were selected for further analysis, and confirmed for the absence of Pfn-1 using antibodies raised against the N- or C-terminus or full-length protein. Confirmatory VASP staining was employed for all lines and HSP70 as loading control. **B** Representative cell images of the actin cytoskeleton of B16-F1, EVM-KO and EVM+Pfn1-KO cell lines, as indicated. Note the incremental decrease of lamellipodial dimension (width) from WT to EVM-KO and EVM+Pfn1-KOs. The scale bar in the B16-F1 panel applies to all subpanels and equals 10 µm. **C** Quantitation of lamellipodial F-actin intensities for the cell lines, shown in (**A**, **B**). Data displayed as superplots as described for Fig. 2B. **D** Quantitation of lamellipodial widths in distinct cell lines, data as in Fig. 2C. Gray lines and error bars in (**C**, **D**) display means and standard errors of means (SEMs). EVM- and quadruple-KO datasets (i.e., the arithmetic means of replicates) in **C**, **D** were statistically compared with B16 WT or EVM-KO, as indicated, using ANOVA with Tukey’s post hoc test; n.s. not significant; *p ≤ 0.05; ****p ≤ 0.001. **E** Representative cell stainings of WT (B16-F1), EVM-KO and selected clones each for Pfn1-KO (clone #20) and EVM+Pfn1-KO (clone #19) for core actin machinery components as indicated. Color codes as described for Fig. 3A, except that Ena/VASP family members were replaced by WRC subunits (Abi and WAVE2, red). **F**–**J** Quantitations of actin core machinery components, all assessed and displayed and color-coded as shown in (**E**) and described corresponding data in Fig. 3. Gray lines and error bars display means and standard errors of means (SEMs), and *n* = measured cells from three (**I**, **J**) or four (**F**, **G**, **H**) independent experiments. As above, all datasets were compared with each other in all combinations using ANOVA with Tukey’s post hoc test; n.s., not significant, *p ≤ 0.05; **p ≤ 0.01; ***p ≤ 0.005; ****p ≤ 0.001.

To clarify these relationships and test the functionality of Ena/VASP proteins in the absence of Pfn1, we generated cells lacking Ena/VASP and Pfn1 activities in combination. Using Ena/VASP triple-KO clone #23.7.66 (EVM-KO)^56^, we disrupted Pfn1 to create three independent EVM+Pfn1 quadruple-KO lines (EVM+Pfn1-KO, clones #10, #19 and #27, Fig. 4A, B). Interestingly, these cells could still form lamellipodia (Fig. 4B), but these were notably smaller and had further reduced width and actin filament mass compared to EVM-KO alone (Fig. 4C, D). Protrusion rates declined progressively from WT to EVM-KO and Pfn1-KO, and were most severely impaired in the combined knockout (Supplementary Fig. 10A, B). Lamellipodial actin assembly rates were more strongly reduced by Pfn1 than Ena/VASP loss of function, with the double knockout exhibiting an intermediate phenotype though, possibly due to impaired adhesion and elevated retrograde flow as compared to Pfn1-KO alone (Supplementary Fig. 10C, D). Cell migration was also most compromised in the EVM+Pfn1-KOs compared to either single KO (Supplementary Fig. 10E, F, for migration tracks see Supplementary Fig. 11, Supplementary Movie 8). Collectively, these results demonstrate that the Pfn1 loss-induced accumulation of Ena/VASP proteins is functionally important, substantially supporting lamellipodial protrusion and migration upon suppression of profilin.

### Combined EVM and Pfn1 loss exerts differential effects on Arp2/3 complex *versus* CP

As shown, Pfn1 deletion caused a strong reduction in Arp2/3 complex and CP levels in the lamellipodium (Fig. 3). In contrast, Ena/VASP loss had the opposite effect, enhancing their accumulation^56^. The newly generated EVM+Pfn1 quadruple-KO clones provided a unique opportunity to dissect whether Arp2/3 complex and CP accumulation are mechanistically linked or differentially regulated. Specifically, we asked whether Ena/VASP removal could override the Pfn1-KO-induced suppression of Arp2/3 complex, or if Pfn1 was dominant in determining Arp2/3 incorporation. Strikingly, Arp2/3 complex accumulation remained suppressed in the quadruple-KO cells, regardless of Ena/VASP status (Fig. 4E, F). Thus, the Ena/VASP-deficiency-induced increase in Arp2/3 complex seen previously^56^ is entirely dependent on the presence of Pfn1. These data establish that Pfn1 plays a dominant role in enabling Arp2/3 complex recruitment to lamellipodia, independent of Ena/VASP proteins, highlighting its unexpected influence beyond formin-mediated actin assembly.

In contrast, analysis of CP revealed an opposite trend. Despite Pfn1 loss, CP accumulation remained elevated in EVM+Pfn1 quadruple-KOs (Fig. 4E, G), consistent with previous observations in EVM-deficient cells and in stark contrast to the reduction seen in Pfn1-KO alone. This indicates that the increased Ena/VASP accumulation in Pfn1-KO cells is responsible for suppressing CP, and that this suppression is reversible upon Ena/VASP removal. To our knowledge, this represents the first experimental separation of Arp2/3 complex and CP regulation at the lamellipodium, demonstrating that their incorporation into actin networks is governed by distinct and independently controlled mechanisms.

We also examined the WRC components Abi and WAVE under these same genetic conditions. Changes in their accumulation did not mirror those of Arp2/3 complex. Specifically, the increase in Arp2/3 complex in EVM-KO cells (Fig. 4F) occurred without a corresponding rise in Abi or WAVE intensity, which remained unchanged (Fig. 4E, H, I), as also reported previously^56^. Similarly, Pfn1 loss led to a strong reduction of Arp2/3 complex without major changes in WRC accumulation (Fig. 4F, H, I). Although Abi and WAVE levels were apparently reduced in the EVM+Pfn1 quadruple-KO cells as compared to Pfn1-KO alone, this was not statistically significant and did not translate into further differences in Arp2/3 complex incorporation (Fig. 4F). These results suggest that while WRC is essential for Arp2/3 complex targeting and activation at lamellipodial tips^37,57^, the observed changes in Arp2/3 complex incorporation are not driven by distinct WRC levels. Instead, they are modulated by specific core regulators, including Pfn1 and Ena/VASP, which control actin assembly at the leading edge.

### Lpd opposes profilin in regulating lamellipodial accumulation of Ena/VASP *versus* CP

**Fig. 5:**
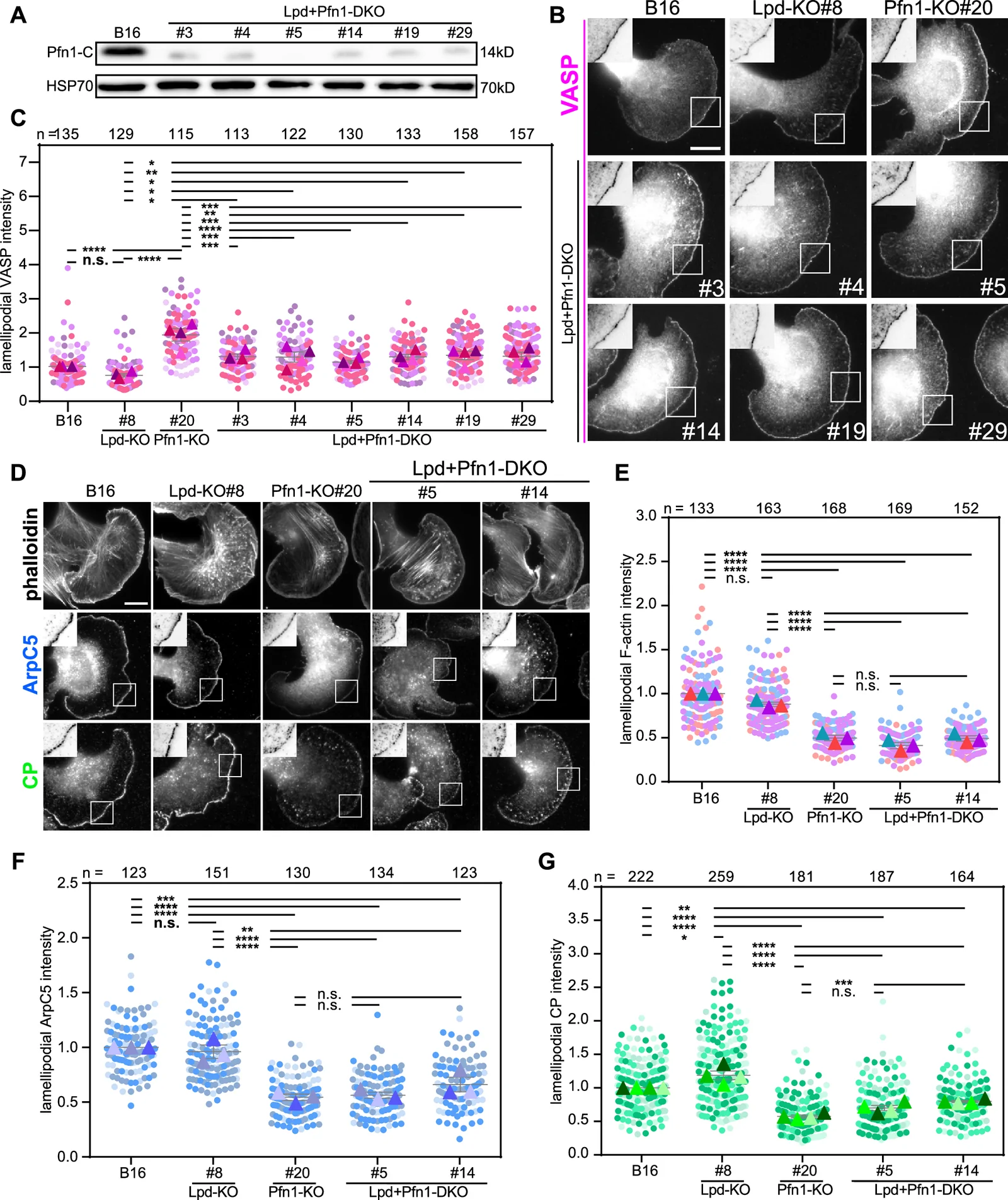
Suppression of profilin expression in the absence of lamellipodin generates intermediate phenotypes for VASP up- and CP downregulation in the lamellipodium. **A** Western blot displaying Pfn1-staining in cell extracts from lamellipodin (Lpd)-deficient lines isolated upon additional CRISPR/Cas9 targeting of the *Pfn1* gene, termed Lpd+Pfn1 double knockout (Lpd+Pfn1-DKO), as compared to B16-F1 control. Note that Pfn1 expression was reduced, but is still detectable in most (five out of six) DKO-clones. DKO cell lines thus constituted Lpd-deficient cells with very low Pfn1 expression. **B** Immunolabeling using VASP-specific antibodies in B16-F1 wildtype (B16), Lpd-KO (clone #8), Pfn1-KO (clone #20) and Lpd1+Pfn1-DKO clones, as indicated (for clone numbers see right bottom corners). White squares depict insets displayed as magnified and inverted images at top left corners of each individual panel. Scale bar: 10 µm. **C** Quantitation of lamellipodial VASP intensities performed in a fashion identical to Fig. 3, but for cell lines as indicated. **D** Comparative stainings for the actin cytoskeleton (phalloidin), Arp2/3 complex (ArpC5, blue) and capping protein (CP, green) in B16-F1 wildtype (B16), Lpd-KO#8, Pfn1-KO#20 and two representative Lpd+Pfn1-DKO (clones #5 and #14), as indicated. White squares in ArpC5 and CP panels depict regions inverted and magnified in top left corners as above. **E**–**G** Quantitation of lamellipodial F-actin, Arp2/3 complex (ArpC5, blue) and capping protein (CP, green) in distinct cell lines as shown in (**D**). Gray lines and error bars in (**C**, **E**–**G**) display means and standard errors of means (SEMs). For statistics in (**C**), selected datasets (i.e., arithmetic means of replicates) were analyzed using ANOVA followed by Bonferroni-corrected pairwise comparisons due to the multitude of samples in case of Lpd+Pfn1-DKOs. All six Lpd+Pfn1 DKO clones were statistically significantly different to Pfn1-KO#20, and the same when comparing them to Lpd-KO#8, except for DKO#5 (n.s.). For statistics in (**E**–**G**), arithmetic means of replicates were compared with each other in all combinations using ANOVA with Tukey’s post hoc test (with comparison of the two DKOs with each other not displayed due to space restrictions); n.s., not significant, *p ≤ 0.05; **p ≤ 0.01; ***p ≤ 0.005; ****p ≤ 0.001.

Quantification of Lpd at the tips of EVM-KO cell lamellipodia confirmed a modest, but statistically significant decrease compared to WT (Fig. 4E, J)^53^. This is still surprising considering the prevailing view of Lpd as an upstream recruitment factor of Ena/VASP^51^. Our data instead support a model where Lpd and Ena/VASP mutually stabilize each other in promoting protrusion, rather than Lpd acting solely upstream—consistent with multiple KO studies failing to confirm the original recruitment model (this study and refs. ^52,53^). Moreover, the increased Lpd signal observed upon Pfn1 loss (see above) was diminished in the absence of both Ena/VASP and Pfn1 (Fig. 4E, J), suggesting opposing effects of Ena/VASP and profilin on Lpd accumulation. To test this further, we suppressed Pfn1 in Lpd-KO cells and examined the impact on Ena/VASP and CP accumulation. Although complete Pfn1 elimination was not possible in the Lpd-KO background, we reduced its expression to ~20% of WT in six independent clones of Lpd-KO#8 (Fig. 5A). All clones were analyzed for VASP accumulation, in comparison to Pfn1-KO and Lpd-KO cells (Fig. 5B, C).

Strikingly, VASP levels in the double-deficient (DKO) clones were intermediate—reduced and increased compared to individual Pfn1- and Lpd-KOs, respectively, and slightly higher than WT—suggesting that Lpd loss to large extent offsets the Pfn1-KO-induced increase in Ena/VASP. This pattern held across all six clones, independent of residual Pfn1 levels (Fig. 5A). Two representative double-KO clones (#5 and #14) were selected for further analyses. In these cells, despite strongly altered VASP levels (Fig. 5C), lamellipodial F-actin or Arp2/3 complex intensities were not markedly affected on average as compared to Pfn1-KO alone (Fig. 5D–F).

In contrast, CP accumulation mostly showed statistically significant changes. Lpd-KO alone increased CP at lamellipodia (Fig. 5D, G), coinciding with reduced VASP (Fig. 5B, C). Conversely, the Pfn1-KO-induced decrease in CP—shown to depend on Ena/VASP (Fig. 4E, G)—was partially reversed in the absence of Lpd, clearly trending upwards in both DKO-clones as compared to Pfn1-KO alone and statistically significant at least for Lpd+Pfn1-DKO #14. CP levels in double-deficient cells thus fell between those of WT and Pfn1-KO cells, confirming the mutually antagonistic relationship between CP and Ena/VASP: lower Ena/VASP correlates with higher CP, and vice versa. Besides these opposing effects on VASP and CP, actin and Arp2/3 complex levels in Lpd+Pfn1 double-deficient lamellipodia appeared more balanced relative to Pfn1-KO cells (Fig. 5D–F).

To independently validate the antagonism between Ena/VASP and CP, we analyzed lamellipodial VASP and Mena accumulations in a CP-deficient cell line^20^. While these cells exhibit chaotic protrusions, lamellipodia are still present^20,21^, which was accompanied by robust VASP and Mena accumulation at their tips (Supplementary Fig. 9A–C). Remarkably, Ena/VASP accumulation in CapZβ-KO cells was even more pronounced than in Pfn1-deficient cells (clone #20), which retain reduced—but not absent—lamellipodial CP (Fig. 3A, C; Supplementary Fig. 9A–C).

These data underscore the reciprocal regulation of Ena/VASP and CP in lamellipodia and set the stage for further mechanistic insight via mathematical modeling (see below).

### Pfn1 loss-induced, differential changes in lamellipodial Arp2/3 complex and VASP accumulation can be rescued by transient Pfn1 expression

**Fig. 6:**
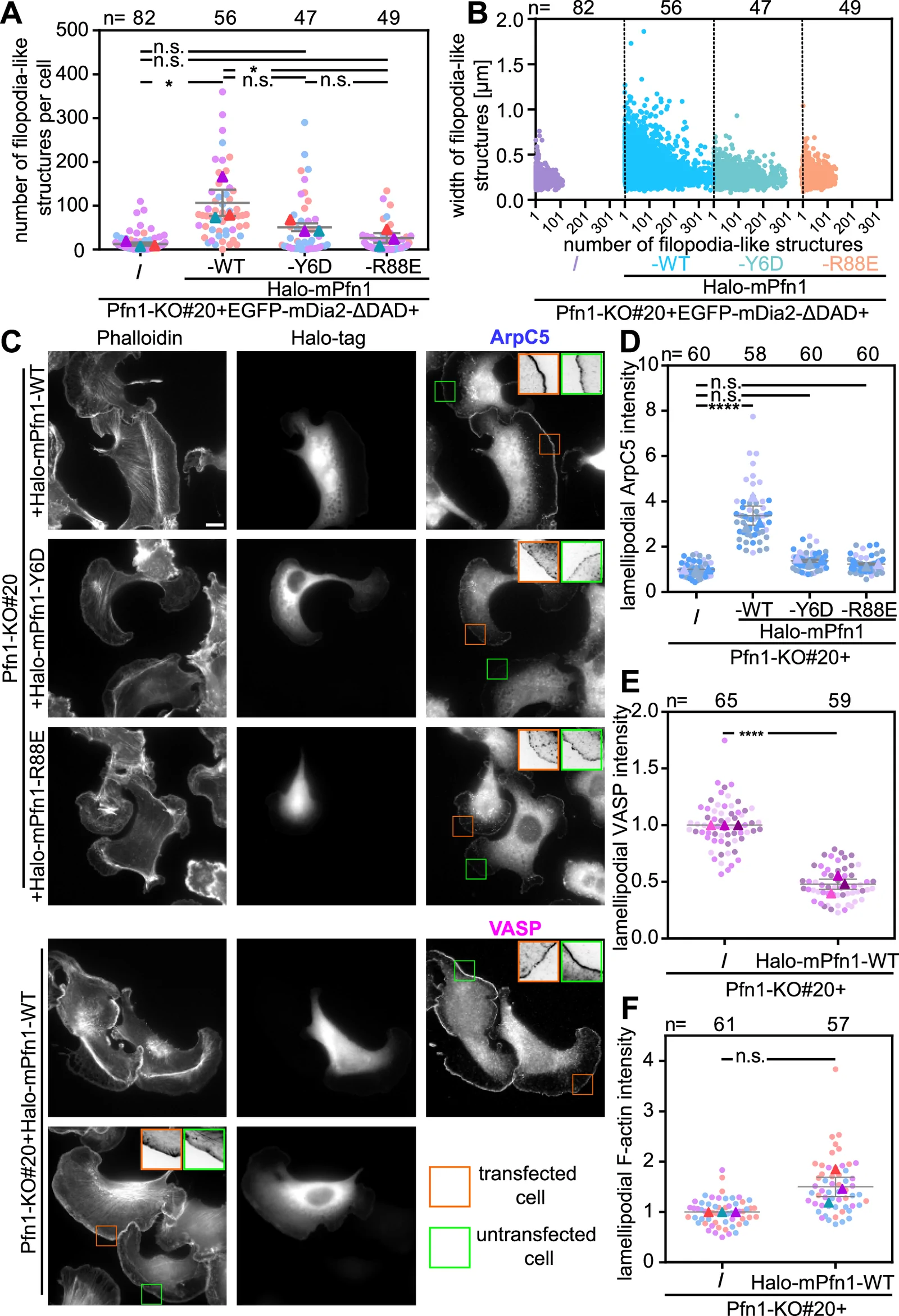
Rescue of filopodial and lamellipodial actin assembly phenotypes by transient transfection with mPfn1. **A** Numbers of filopodia-like structures formed in live Pfn1-KO #20 cells without and with transient co-expression of constitutively active mDia2 (mDia2-ΔDAD) and Halo-tagged, murine Pfn1 (Halo-mPfn1) wildtype (WT) or polyproline (Y6D)- or actin binding (R88E)-deficient variants. Data were quantified from movies of 47 to 82 cells, as indicated. Gray lines and error bars are means and standard errors of means (SEMs), respectively. For statistics, all datasets were compared with each other using ANOVA with Tukey’s post hoc test; n.s., not significant, *p ≤ 0.05. **B** Width of club-shaped, individual, mDia2-induced filopodia known to correlate with the extent of actin filament packing in these structures^61,82^ plotted against their number per cell for each aforementioned, experimental condition, as indicated (color-code). **C** Representative images of fields of Pfn1-KO #20 cells with (orange squares) or without (green squares) expression of mPfn1 variants (see Halo-tag channel), as indicated, and analyzed for the lamellipodial accumulation of ArpC5 (blue), VASP (pink) or actin filaments (bottom). Orange and green squares indicate the cellular positions of magnified, inverted insets shown at the top right of each panel. Scale bar: 10 µm. **D** Quantitation of ArpC5 accumulation in rescued cells as compared to non-transfectants analyzed in independent, non-transfected samples processed in parallel. Gray lines and error bars display arithmetic means and standard errors of means (SEMs). For statistics, non-transfectants as controls were compared with each rescue condition using ANOVA with Dunnett’s posthoc test; n.s., not significant, ****p ≤ 0.001. Quantitation of lamellipodial VASP (**E**) and F-actin (**F**) intensities in Pfn1-KO #20 cells with or without rescue expression with Halo-mPfn1-WT. Data are displayed as described for (**D**), with gray lines and error bars constituting means and standard errors of means (SEMs), respectively. For statistics in (**E**, **F**), normally-distributed datasets were analyzed using two-sample *t*-tests; n.s., not significant, ***p ≤ 0.005.

The lamellipodial phenotypes described upon Pfn1 loss of function were obtained from clonal cell lines permanently growing upon disruption of all existent Pfn1 alleles. To exclude the possibility that phenotypes were caused by compensatory changes in gene expression and instead confirm that they were indeed caused by the absence of Pfn1 function, we aimed to rescue phenotypes by transient transfection of Pfn1-variants, Halo-tagged with a 10 aa-linker previously established by biochemical experiments not to interfere with its activities on actin assembly^58^. We generated a murine version of Halo-tagged, wildtype Pfn1 (mPfn1-WT, see “Methods”) and first tested its activity in filopodia formation upon co-expression in Pfn1-KO #20 with constitutively active mDia2. Cellular profilin activities are well established to require its interactions with both actin and polyproline ligands, such as in formins, previously reported to be abrogated by single point mutations^41,59,60^. For suppressing polyproline- and actin-binding, we here employed Y6D and R88E-mutants of murine Pfn1, respectively^58^. Notably, co-transfection with mPfn1-WT, but much less so aforementioned mutants, increased the number of filopodia-like structures formed per cell (Fig. 6A, Supplementary Movie 9). As the width of mDia2-induced filopodia-like structures was previously correlated with actin filament numbers nucleated at their tips^61^, we also plotted tip width against numbers, illustrating a dramatic increase in Pfn1-deficient cells re-expressing, tagged mPfn1-WT, but much less so for polyproline- or actin binding-deficient mutants (Fig. 6B). More specifically, a much wider distribution of widths of individual, filopodia-like structures was observed in Pfn1-KO #20 cells upon co-expression of EGFP-mDia2-ΔDAD with mPfn1-WT (blue in Fig. 6B) *versus* mPfn1 variants abrogated in polyproline binding (green) or actin binding (orange) or without rescue construct (purple in Fig. 6B). These data set the stage for probing potential rescue of changes in lamellipodial Arp2/3 and VASP accumulation. Strikingly, transient mPfn1-WT re-expression dramatically increased lamellipodial Arp2/3 complex accumulation only in transfected cells of the same field of view, which was again not seen for the mutants (Fig. 6C, D). The opposite was seen for VASP, which was reduced to half of intensities upon mPfn1-WT rescue as compared to non-transfected, neighboring cells (Fig. 6C, E). Consistently, lamellipodial F-actin was also clearly increased (Fig. 6C, F), albeit not quite in a statistically significant fashion (*p* ≤ 0.059) and to full extent considering WT cell levels (Fig. 2B), for reasons that remain to be determined. Importantly, the fact that these phenotypes were largely rescued by transient mPfn1-WT re-expression demonstrates that they are reversible on a short timescale and therefore unlikely to reflect long-term, compensatory gene-expression changes in the clonal knockout lines. Therefore, these data clearly establish that lamellipodial phenotypes as exemplified here by differential Arp2/3 complex and Ena/VASP accumulations are largely caused by the sole absence of functional Pfn1.

### Modeling reveals competitive membrane recruitment of Ena/VASP and CP as a key control mechanism of lamellipodial protrusion

**Fig. 7:**
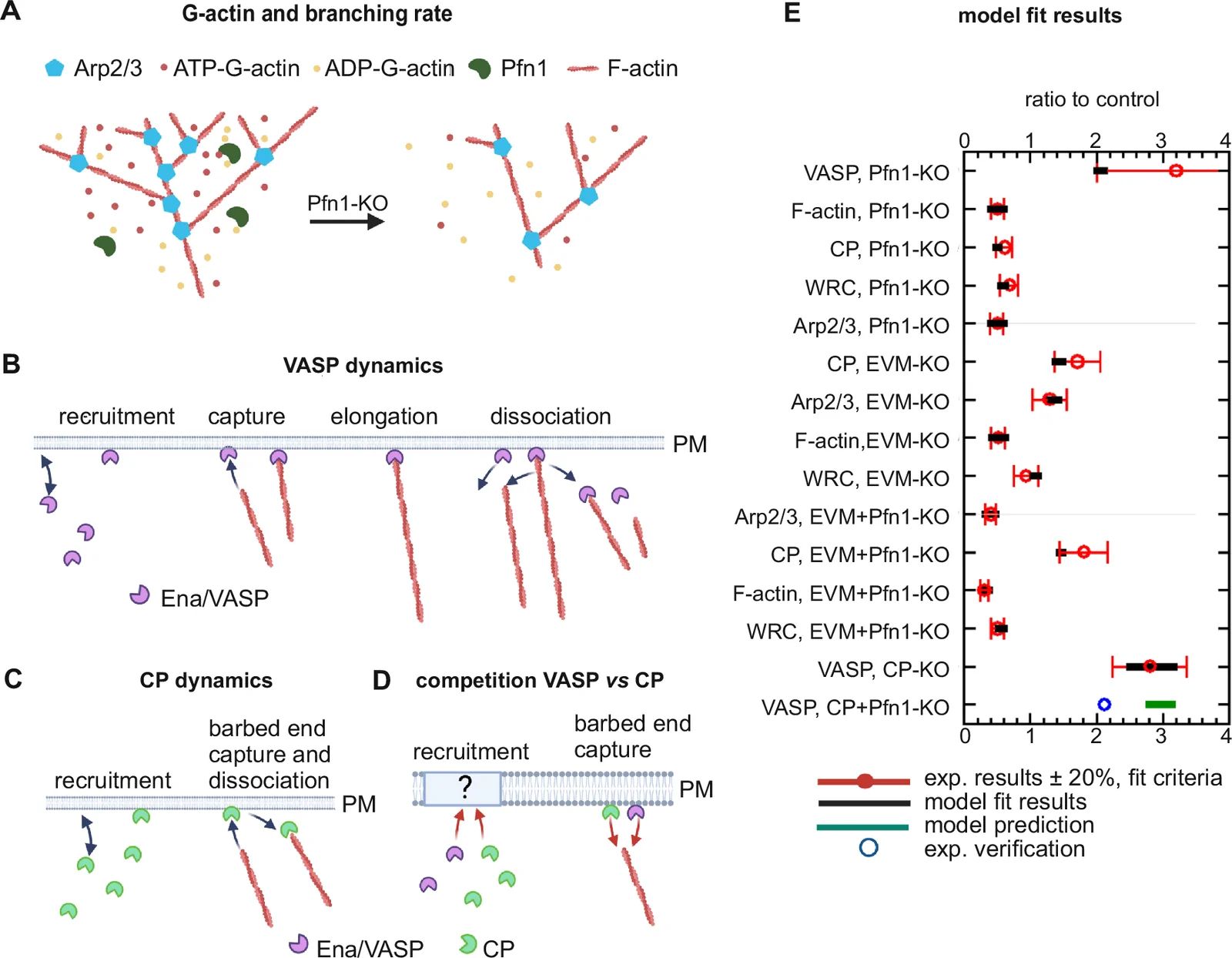
Essential assumptions defining the mathematical model and modeling results. **A** Summary of assumed consequences on Arp2/3-dependent actin networks of profilin suppression (Pfn1-KO). Molecules are not drawn to scale, and Pfn1 exchange activity (ADP to ATP-actin) is symbolized by binding to both actin monomers. Note reduced actin monomer, polymer as well as branch concentrations in Pfn1-KO. **B** Different stages in the lifecycle of Ena/VASP family members as assumed in the model. Individual, functional units of cytosolic VASP (or Evl or Mena; tetramerization not indicated) can go through distinct stages, with elongation being followed by dissociation of barbed end-bound VASP as dominant process in the model (black arrow pointing to the right), but other forms of dissociation are also possible (black arrows to the left). Note that the model does not specify molecular mechanisms of Ena/VASP recruitment^14^ **C** Summary of assumptions on CP dynamics. Note the absence of a filament elongation phase and the immediate removal from the membrane of the barbed end as direct consequence of capture by CP. **D** In the model, Ena/VASP and CP experience two separable types of competition, (i) for barbed ends (right) and (ii) for recruitment to the plasma membrane (PM). In spite of being essential for the model to reproduce the experimental observations, the molecular nature of the latter competition mechanism remains to be established (question mark). **E** Fit results (black) expressed in fold WT (ratio to control) for the 14 fit criteria (red) in distinct KO cell populations. We considered a criterion as met if the model output was in the range indicated by the red lines corresponding to ±20% of the experimentally determined, arithmetic mean, except for “VASP, Pfn1-KO” that required a larger acceptance range (also see Supplementary Fig. 12, Fig. M4). The fit results are 2218 parameter value sets each meeting the criteria. They were found by sampling 5 10^9^ parameter value sets (for details see Supplementary Fig. 12). The green line is a model prediction based on the fits to the 14 criteria, and the blue symbol constitutes its experimental confirmation in CP+Pfn1 KO, specifically CapZβ + Pfn1DKO clone #11 (Supplementary Fig. 14).

To mechanistically integrate the molecular interactions underlying lamellipodial actin remodeling, we developed a mathematical model incorporating key players studied experimentally: actin, Arp2/3 complex, WRC, CP, and Ena/VASP (Fig. 7A–E, Supplementary Figs. 12 and 13). The model describes dynamic interactions at the leading edge membrane, including key player binding, their modes of recruitment and impact on actin polymerization activity. The rate constants of these interactions, the cytosolic concentrations of involved molecules and measured actin assembly rates in different KO cell lines are all parameters in the model (Supplementary Tables 2 and 4 in Supplementary Fig. 12). Parameters were constrained by experimental measurements resulting in 14 criteria derived from fluorescence intensity data (Supplementary Fig. 12, Supplementary Table 3). These criteria constituted fluorescence intensity ratios of aforementioned players between different knockout conditions and control. Fitting to those criteria generated >2200 parameter value sets all meeting the criteria within given acceptance ranges (Fig. 7E).

Pfn1 knockout was modeled as a reduction in branching activity^62,63^, based on the experimentally observed decrease in total actin expression (~40% of WT, Fig. 1G), polymerizable G-actin, and Arp2/3 complex incorporation (Fig. 3A, B). As profilin catalyzes nucleotide exchange on G-actin, its loss was assumed to reduce ATP-G-actin to ~20% of WT (Supplementary Fig. 12). This decrease in polymerizable G-actin entailed fewer branch points and decreased lamellipodial F-actin density (Fig. 7A, E, Supplementary Fig. 13). WRC levels were also predicted to decline, consistent with reduced involvement in branching (Fig. 7E).

Critically, the model captured a key experimental observation: increased Ena/VASP (EVM) accumulation at the leading edge membrane despite reduced F-actin (and thus barbed end) density in Pfn1-KO cells (Figs. 2B, 3A, D, E, 5B–C, E). This indicates that EVM recruitment is not limited by barbed end availability and requires a recruitment process for EVM to the leading edge membrane independent of barbed ends (Fig. 7D). Similarly, CP also still accumulated in Ena/VASP-KO cells despite reduced filament density (Fig. 4C, E, G)^56^, supporting the notion that both EVM and CP are recruited independently of barbed ends to the protruding cell front (Fig. 7B, C).

The modeling results further revealed that Ena/VASP and CP must compete for shared recruitment sites at the membrane—beyond just barbed ends—as only this assumption reconciled their reciprocal regulation in the KO data (Fig. 7D). Upon Pfn1 loss, the model reproduced increased occupancy of these sites by Ena/VASP, leading to reduced CP accumulation, both caused by differential rate constants of binding to these sites. This competitive recruitment model matched experimental results across multiple KO combinations (Fig. 5), providing a mechanistic explanation for the strict antagonism observed in situ.

Moreover, additional model fitting showed that further reductions in CP levels (beyond the 20% experimental threshold) would drive even higher Ena/VASP accumulation (Supplementary Fig. 12, Figs. M4–M6), reinforcing this antagonistic relationship. Importantly, this framework deviates from classical models that assume CP and Ena/VASP to compete solely for barbed ends^64^. Instead, our findings establish membrane recruitment site competition between these two factors as a previously underappreciated regulatory layer in protrusion control (Fig. 7D, question mark). The model thus not only captures known dependencies (e.g., profilin’s influence on polymerizable actin and branching), but also proposes a testable, mechanistic basis for the observed Ena/VASP–CP antagonism (Fig. 4E, G, Supplementary Fig. 9A–C).

To further explore the predictive power of our model, we sought to probe the impact on a specific criterion, in this case VASP intensity, in a hitherto unstudied condition. We chose to combine Pfn1-KO with CP-KO for this, both of which displayed increased VASP intensities as individual KOs (Fig. 7E), to ask whether their simultaneous removal would have an additive effect on lamellipodial VASP accumulation. We predicted the outcome of the CP+Pfn1-double (D)KO using the model fitted to the 14 criteria mentioned above (Fig. 7E). This revealed a strong increase in VASP, albeit not exceeding the levels observed in the individual KOs. The blue symbol in Fig. 7E shows the result of the experimental verification (CP+Pfn1-DKO#11), which was not part of the fit criteria (for generation and preliminary characterization of two independent double-KO clones, #11 and #12, see Supplementary Fig. 14). Similar to the prediction, experimentally determined VASP levels at lamellipodial edges of the CP+Pfn1-DKOs were again increased as compared to WT, but a little lower than the levels found in individual KOs (Fig. 7E, Supplementary Figs. 9 and 14). These data thus confirmed the absence of additive VASP accumulation upon combining genetic CP and Pfn1 disruption, as predicted by the model.

## Discussion

Dynamic actin filament remodeling, such as in the lamellipodium, involves several widely conserved factors, whose function remain incompletely understood despite extensive study. This is particularly true for core regulators like profilin (Pfn), Ena/VASP family polymerases and heterodimeric capping protein (CP). Here, we investigate their complex interrelationships within Arp2/3 complex-dependent lamellipodial protrusions. Historically, Arp2/3 complex-mediated actin nucleation and branching were considered antagonistic to formin-dependent elongation, which relies on Pfn. Thus, Arp2/3-generated branched networks and formin-driven linear filaments were viewed as antagonizing structures competing for a limited pool of polymerizable actin^33^. However, our findings, along with previous studies, challenge this oversimplified view, besides the fact that Arp2/3 complex is now also recognized to generate linear filaments downstream of SPIN90, not just branches^34,65^.

Using cell lines with minimal Pfn expression, we confirm its essential role in formin-dependent actin structures, such as filopodia triggered by mDia2. Surprisingly however, Pfn also enhances Arp2/3 complex activity in lamellipodia, promoting actin branching. Pfn depletion reduces, but does not eliminate, Arp2/3 complex incorporation into lamellipodia, likely due to a severe drop in polymerizable actin, partly driven by a ~60% decrease in total actin levels. Suppression of actin expression might be mediated by reduced serum response factor signaling due to increased, cytosolic myocardin-related transcription factor sequestration^66^, but this hypothesis requires experimental verification.

The transformation of lamellipodia in the virtual absence of Pfn includes a prominent accumulation of Ena/VASP family polymerases and a concomitant suppression of its antagonist, CP. Disrupting both Ena/VASP and Pfn1 reveals that Ena/VASP controls CP but not Arp2/3 incorporation, since the latter remains low even when Ena/VASP is removed in Pfn1-KO cells. Thus, while VASP antagonizes CP, Pfn promotes Arp2/3 complex activity, contrary to previous assumptions^31,32^.

These findings highlight that each of these factors do not solely influence each other indirectly through tuning actin assembly dynamics, but through direct reciprocal effects. For instance, Pfn1 depletion-induced VASP accumulation is partly reversed by suppressing its interactor Lpd. This in turn alleviates the CP reduction seen with Pfn1 loss alone, all supporting a direct mutually antagonistic relationship between Ena/VASP and CP in lamellipodia.

Our mathematical modeling robustly recapitulates these observations in Pfn1- or combined Pfn1- and Ena/VASP-KO cell lines. The model shows that our results are compatible with current ideas on the role of most of the model components but provides further information with regard to VASP and CP: The antagonism in VASP *versus* CP accumulation is best explained by an additional, competitive membrane recruitment mechanism rather than their sole direct competition for barbed ends. Furthermore, obtained modeling solutions represented broad parameter ranges, illustrating the robustness of our framework in describing actin network remodeling (Fig. 7, Supplementary Fig. 12).

The model also provides quantitative insights into the relationships between involved factors and/or their interactions (see scaled variables in Figs. M3 and M6 in Supplementary Fig. 12). One key prediction is the relatively rare interaction between WRC and Arp2/3 complex (only ~1% of free WRC molecules at the membrane bound to Arp2/3 in the stationary model), similar to the low percentage of WRC/Arp2/3-complexes capturing a filament. Despite being crucial for lamellipodia assembly^46,48,49^, this indicates that Arp2/3-mediated branching of filaments may appear far less frequently than their elongation. Moreover, filament-bound WRC molecules are comparable to or slightly more abundant than free WRCs, suggesting a significant role for the complex in barbed end elongation as distributive polymerase^67^. Yet, VASP-bound filaments outnumber WRC-bound ones by up to tenfold, emphasizing the dominance of molecular players solely engaged in elongation. Interestingly, free VASP at the membrane is even more abundant than filament-bound VASP, matching CP levels and explaining why unbound filaments are scarce (Figs. M3 and M6 in Supplementary Fig. 12).

All these data suggest that lamellipodial actin filament generation and elongation are tightly coordinated across its core regulators. However, the molecular basis of the Ena/VASP and Arp2/3 complex antagonism remains unclear. Since WRC biases Arp2/3 complex activation towards the membrane, Ena/VASP-bound filaments may be suboptimal substrates for branching.

Additionally, the precise mechanism underlying Ena/VASP and CP competition for leading edge targeting remains unknown, though our experiments and modeling suggest it must be largely independent of barbed end binding. Future studies will thus be required to reveal the molecular identities and regulation of these shared membrane recruitment sites. Clearly, no single factor established to bind both, CP and Ena/VASP, is currently known. Notably however, our previous BioID data indicated that Ena/VASP and CP can exist in close proximity (<10 nm)^53^, suggesting indirect interactions within larger protein complexes potentially mediating their mutually antagonistic behavior. Promising candidate constituents of such complexes certainly include established direct interactors of either one of the two, in particular those harboring potential interaction surfaces for binders of the respective other partner, like e.g., the cortactin and CP binding, so called CD2-associated protein (CD2AP) with its multiple, polyproline-binding SH3-domains or its homolog CIN85 (see^16,68^). How such candidates relate to the bona-fide leading edge-enriched Ena/VASP interactors Lpd and WRC^50,69^ or the CP-target CARMIL^70,71^ remains to be determined. Future work will aim to resolve these outstanding questions.

## Methods

### Cell culture and transfections

B16-F1 mouse melanoma cells (purchased from American Type Culture Collection, Manassas, VA, CRL-6323) were cultivated according to standard tissue culture conditions. In brief, cells were grown at 37 °C in an atmosphere containing 7.5% CO_2_ in Dulbecco’s modified Eagle medium (DMEM) containing 4.5 g/L glucose (Life Technologies, Thermo Fisher Scientific, Germany) and 10% fetal calf serum (FCS; Gibco, Paisley, UK), 2 mM glutamine and 1% penicillin-streptomycin (Thermo Fisher Scientific). Wildtype B16-F1 cells were split every other day at densities at least 1:10 and 1:20, and distinct knockout cell lines described below adapted for splitting dilutions according to their respective growth capabilities. Previously published CRISPR-KO cell lines employed for analyses and/or used as parental lines for additional gene disruptions were EVM-KO (clone #23.7.66)^56^ and Lpd-KO (clone #8)^52^.

For transfections of cells with respective DNA vectors, JetPrime transfection reagent (Polyplus Transfection, Illkirch, France) was used according to the manufacturer’s instructions. For microscopy experiments, B16-F1 cells were seeded onto glass coverslips pre-coated for 1 h at room temperature with 25 ng/μL laminin (L-2020; Sigma-Aldrich, Germany) in coating buffer (50 mM Tris-HCl, pH 7.4, and 150 mM NaCl) if not stated otherwise.

*Dictyostelium discoideum* AX2-214 wildtype (WT) and PfnI/II-deficient cells^40^ were cultivated at 21 °C in HL5-C medium containing glucose (HLC0102, Formedium) supplemented with 50 µg/mL penicillin and 40 µg/ml streptomycin (Carl Roth) on polystyrene-coated petri dishes. For homologous recombination, cells were transfected by electroporation as described below.

### DNA constructs

For rescue experiments shown in Fig. 6 and Supplementary Movie 9, Halo-Tagged Pfn1-variants harboring a 10aa-linker were engineered precisely as described for CMV promotor-controlled, human Pfn1-constructs characterized and published previously, but for human Pfn1 sequences replaced with respective murine versions. Specifically, murine Pfn1-WT (mPfn1-WT, NCBI accession number: NM_011072.4) and plasmid backbone were amplified using Q5® High-Fidelity DNA Polymerase (NEB, M0491S) and primers 5′-AGGATCTGTACTTTCAGAGCTCCGGACTCAGATCTCGAGCTCAAG-3′ (forward) and 5′-GGTGGCGACCGGTAGCGC-3′ (reverse) from template Addgene #54940 (https://www.addgene.org/54940/), and Halo-tag using primers 5′-TAGCGCTACCGGTCGCCACCATGGCAGAAATCGGTACTG-3′ (forward) and 5′-GCTCTGAAAGTACAGATCC-3′ (reverse) from template CMV Halo-10-HsPfn1^58^, followed by the fusion of these amplicons with NEBuilder® HiFi DNA Assembly Cloning Kit.

Point mutants deficient in poly-proline (Y6D) or actin binding (R88E) were subsequently generated using Q5® Site-Directed Mutagenesis Kit (NEB, E0554S) and forward primers 5′-GTGGAACGCC**GAT**ATCGACAGCC-3′ (with reverse 5′-CCGGCCATGGAAGCTTGA-3′ for amplification) and 5′-AATGGATCTT**GAA**ACCAAGAGCACCG-3′ (with reverse 5′-GTAAATTCCCCGTCTTGCAG-3′), respectively.

Further constructs for live cell fluorescence imaging of actin cytoskeleton dynamics were EGFP-tagged, human β-actin (PT3265-5, Clontech Inc., Mountain View, CA, USA), mDia2-FL and -ΔDAD^61^, ArpC5B^72^ (more recently also called ArpC5L) as well as FMNL3-ΔDAD and -A275E^73^.

### Gene disruptions in B16-F1 melanoma and *D. discoideum*

Gene disruptions by CRISPR/Cas9-mediated genome editing in B16-F1 melanoma followed by single cell cloning and expansion were performed essentially as described before^73^. Specifically, disruption of the gene locus encoding profilin 1 (*Pfn1*) in each cell line (B16-F1 wildtype, EVM-KO#23.7.66, Lpd-KO#8, CapZβ-KO #10 or Pfn2-KO#41, for generation of the latter see below) was done by targeting exon 1 and using guide-sequence 5′-TGTCAGGACGCGGCCATCGT-3′, cloned into CRISPR expression vector pSpCas9(BB)-2A-Puro (Addgene 48138). Successful knockout clones were initially screened by Western blotting followed by Sanger sequencing of cloned genomic DNA amplicons (for Pfn1 KO-lines #6, #10 and #20) or by TIDE-sequencing in case of EVM+Pfn1-DKOs, Lpd+Pfn1-DKOs and CapZβ + Pfn1-DKOs. For genomic DNA extractions prior to sequencing, cells were pelleted and incubated at 55 °C overnight in lysis buffer (100 mM Tris-HCl pH 8.5, 5 mM EDTA, 0.2% SDS, 200 mM NaCl) containing 40 μg/mL proteinase K, followed by nucleic acid extraction by phenol/chloroform precipitation. The targeted locus was PCR-amplified with forward and reverse primers 5′-ATCTGAAGCTTAGCTAACAGAGCCGCGTTC-3′ and 5′-ATCTGCTCGAGAGGGTCTAAGGCCGGTCAC-3′, respectively. In case of Pfn1 single KOs, amplicons were purified with a NucleoSpin Gel and a PCR clean-up kit following manufacturer’s instructions (Macherey&Nagel), inserted into a Zero Blunt TOPO vector using a commercial Zero Blunt TOPO Cloning Kit (Invitrogen) and transformed into competent bacteria. Upon inoculation and growth of single bacterial colonies, derived plasmid DNAs were purified using a NucleoSpin Plasmid kit (Macherey&Nagel) and sequenced by Eurofin Genomics (Ebersberg, Germany) using sequencing primer 5′-CAGGAAACAGCTATGAC-3′. Knockout lines obtained upon targeting of the *Pfn1*-locus in EVM-KO, Lpd-KO or CapZβ-KO cells were sequenced by TIDE-sequencing^74^. Clones with mutations exclusively causing frameshifts on all detected alleles were selected for further characterization.

The attempt to disrupt Pfn1 in the Pfn2-KO background (clone #41) in Supplementary Fig. 3C–E was not followed by sequencing, as no reduction of Pfn1 expression upon subcloning could be detected by Western Blotting (Supplementary Fig. 3E). The stable Pfn2-KO (clone #41) used for this experiment was originally generated as follows: Exon 1 of the *Pfn2* gene was targeted using guide-sequence 5′-GTCTGGGCAGCCACGGCCGG-3′, again inserted into pSpCas9(BB)-2A-Puro vector (Addgene 48139) and transfected into B16-F1 wildtype cells for stable KO generation essentially as described for the *Pfn1* locus above, except that Lipofectamine® 2000 Transfection Reagent (Invitrogen) was used following manufacturer’s instructions. Upon clone selection by Western blotting, PCR fragments from the amplified target region of each clone were inserted into pcDNA3 vector, followed by Sanger sequencing of individual colonies. Clone #41 turned out to lack any wildtype allele and was thus selected for further experiments.

The gene locus giving rise to expression of the Arp2 subunit of Arp2/3 complex (*Actr2*) was targeted in either WT B16-F1 or Pfn1-KO (clone #20) using 5′-TCCTTCCAGTTTGTGAAGTG-3′ as guide sequence. Upon editing and selection of promising clones by Western blotting, the selected region within exon 2 was PCR-amplified with oligonucleotides 5′-TAAAAGCCAGGGTAAGCAAGGC-3′ and 5′-TTCTGACCCTCCCTTTGCTTTT-3′ as forward and reverse primers, respectively, and analyzed by TIDE-sequencing.

Disruption of the *proC* gene in *D. discoideum* as shown in Supplementary Fig. 4A, B was performed as follows: The targeting vector was constructed by amplification of a PstI/BamHI 425-base pair 5′-fragment and a SalI/HindIII 553-base pair 3′-fragment flanking the *proC* gene. Amplified fragments were subsequently inserted into the corresponding restriction enzyme sites of plasmid pLPBLP containing a blasticidin S resistance (Bsr) cassette^75^. The resulting vector was cleaved with BamHI and SalI, and used to disrupt the *proC* gene encoding PfnIII. Primer sequences for the 5′ fragment were 5′-CGCGGATCCATCTGATTGATTTATATTG-3′ and 5′-GATCTGCAGCTTGCCAAGTCATTATTTC-3′, and for the 3′ fragment 5′-GAGAAGCTTCCATATGTATAATATCTCCTTC-3′ and 5′-TATGTCGACGTTTTCGAAATTTTCTATAATCTTG-3′. The fidelity of the targeting vector was verified by sequencing. The PfnIII-targeting construct was then transfected into WT or PfnI/II-KO cells by electroporation using the Xcell Gene Pulser (Bio-Rad) following preset protocol 4-2-6. Transfected cells were selected 24 h after transfection with 10 μg/ml blasticidin (Invivogen) for 7 days and then maintained in growth medium in the absence of blasticidin. Null-mutants were identified by diagnostic PCRs of genomic DNA as described previously^75^. The primers used for analysis were AU: 5′-GTTTCGATCTGGTTGTGAATGGAATGG-3′, AD: 5′-ACATATAAAAATTCTCGCGTTAAATACG-3′, AD2: 5′-CAAAGGTGATACCCAATCAACTG-3′ and Bsr: 5′-CAAACAGTTACTCGTCCTATATACG-3′.

### Immunoblotting and quantitation of Pfn2 or actin expression levels

For preparation of whole cell extracts for immunoblotting, cells were washed once with PBS followed by treatment with lysis buffer (2% SDS, 10% glycerol, 63 mM Tris-HCl pH 6.8) and sonicated to shear genomic DNA. Upon addition of β-mercaptoethanol and bromophenol blue to obtain Laemmli buffer, all lysates were heated for 15 min at 95 °C and stored at −20 °C before usage. To determine expression levels of respective proteins or confirm CRISPR/Cas9-mediated gene disruptions, proteins were separated by SDS-PAGE and transferred onto PVDF membranes for immunoblotting following standard procedures. Primary antibodies were as follows: homemade, polyclonal anti-profilin1 raised against the full length protein (Pfn1, 1:1000, kindly provided by Prof. Dr. Walter Witke, University of Bonn, validated by CRISPR-KO)^76^; polyclonal anti-Pfn1 raised against the N-terminus (Pfn1-N, P7749, 1:3000, Sigma-Aldrich, validated by CRISPR-KO); polyclonal anti-Pfn1 raised against the C-terminus (Pfn1-C, P7624, 1:3000, Sigma-Aldrich, validated by CRISPR-KO); monoclonal anti-Pfn2 (sc-100955, 1:200, Santa Cruz Animal Health, validated by CRISPR-KO); homemade; polyclonal anti-VASP (5500-A, 1:2000, validated by CRISPR-KO)^56^; rabbit monoclonal anti-Arp2 (ab129018, 1:2000, Abcam, validated by CRISPR-KO); monoclonal anti-GAPDH (MAB374, 1:10000, MERCK); monoclonal anti-HSP70 (also known as HSPA8/HSC70, sc-7298, 1:10000, Santa Cruz Animal Health). HRP-conjugated, secondary antibodies used were goat anti-mouse IgG (H + L) (111-035-062, 1:10000, Dianova) or goat-anti-rabbit IgG (H + L) (111-035-045, 1:10000, Dianova). Chemiluminescence obtained upon incubation with ECL Prime Western Blotting Detection Reagent (GE Healthcare) was recorded using an ECL Chemocam imager (Intas, Germany).

For determination of Pfn2 expression levels, quantitative Western blots were performed essentially as described^38^. Briefly, serial dilutions of recombinant profilin2 were run alongside TCA-precipitated lysates from B16-F1 wild-type and profilin1 knockout cells on 15% SDS-PAGE gels. Proteins were transferred to PVDF membranes (Merck) using Tris-Glycine buffer. Membranes were blocked with 5% milk in TBS-T (TBS + 0.2% Tween20) for 1 h at RT, then incubated with anti-profilin2 antibody (1:200, #sc-100955, Santa Cruz). Infrared-labeled donkey anti-mouse secondary antibodies (1:10,000, #925–32212, #926–68073, LI-COR) were used for detection on a LI-COR Odyssey CLx. Fluorescence intensities were analyzed using ImageJ. Equal-sized rectangular areas were selected for each protein band. Intensity profiles were generated using the “Plot Lanes” function, and background signal was subtracted by adjacent background regions. Signal intensities, represented as the area under the intensity profiles, were integrated using the tracing tool. A standard curve was generated by plotting integrated intensities against reference profilin2 protein amounts and fitting a linear function. This standard curve was used to calculate profilin2 mass per cell. The concentration of profilin2 was then calculated as follows:

1 $${c}_{{{{\rm{Profilin}}}}2}=\,\frac{{m}_{{{{\rm{Profilin}}}}2}}{{{{{\rm{Mw}}}}}_{{{{\rm{Profilin}}}}2}\cdot 0.5\,\cdot \,{V}_{{{{\rm{B}}}}16-{{{\rm{F}}}}10}}$$

with m_pro2_ being the mass of profilin2 per cell, Mw_pro2_ being the molecular weight of mouse profilin2 (15,032.21 g/mol) and V_B16-F10_ being the total cell volume of B16-F10 cells (∼1500 µm^3^, see Supplementary Fig. 1 in ref. ^38^), which we assumed to be similar to B16-F1. We factored in only half of the total cellular volume because profilin is largely excluded from organelles that occupy roughly 50% of the cell volume.

For quantification of global actin levels, proteins in total cell lysates were separated by SDS-PAGE and blotted onto nitrocellulose. The blots were then either probed with a rabbit polyclonal pan anti-actin antibody^21^ (1:1000) or an anti-β-tubulin antibody (MAB1864, Merck Millipore, 1:500) overnight. Primary antibodies in immunoblots were visualized by enhanced chemiluminescence (ECL) using peroxidase-coupled anti-rabbit IgG (111-035-003, Dianova, 1:10,000) or anti-mouse IgG (115-035-062, Dianova, 1:10,000) using the ChemiDoc MP Imaging System driven by Image Lab software (Biorad, Hercules, CA, USA). After background subtraction of 16-bit images, band intensities of actin were normalized to respective tubulin signals, and relative protein levels calculated in Pfn1-KO mutants compared with B16-F1 control (WT).

For determination of F-/G-actin ratios in B16-F1 WT and Pfn1-deficient cells, nearly confluent cultures, corresponding to about 5 10^6^ cells, were first rinsed twice with ice-cold PBS. Cells from each plate were detached from the plate with a cell scraper using 300 µL of cold lysis buffer containing 20 mM Hepes, pH 7.2, 100 mM NaCl, 10 mM KCl, 2 mM MgCl_2_, 5 mM KPO_4_, 5 mM EGTA, 5 mM ATP, 2 mM DTT, 5% sucrose, 0.5% Triton X-100, 0.5% NP-40, 5 mM benzamidine (434760, Sigma-Aldrich), 0.1 mM AEBSF hydrochloride (A1421, Applichem) and 0.75 µM phalloidin (2141, Sigma-Aldrich). The crude lysates were then transferred into 1.5 mL reaction tubes and placed onto a rotary shaker for further homogenization at 4 °C. After 30 min, 200 µL samples were centrifuged at 150,000 *g* for 1 h using an Optima Max-XP tabletop ultracentrifuge (Beckman Instruments, Palo Alto, CA, USA). Subsequently, the samples of the supernatant and the pellet fractions were brought to 300 µL with SDS sample buffer, and aliquots subjected to SDS-PAGE followed by immunoblotting. The blots were then probed with aforementioned rabbit polyclonal pan anti-actin antibody and visualized by ECL using peroxidase-coupled anti-rabbit IgG as described above. After background subtraction of 16-bit images, actin intensities were determined by band densitometry using ImageJ software, and ratios of actin levels in pellet and supernatant fractions calculated in Pfn1-KO mutants *versus* B16-F1 controls.

### Phalloidin staining, immunolabeling and quantitation of lamellipodial features or frequency of filopodia formation

For immunolabeling or phalloidin staining, cells were seeded onto glass coverslips coated with laminin as described above and allowed to spread overnight. Both were done using standard procedures essentially as described in detail before^69^.

For phalloidin staining, cells were fixed for 20 min with a mixture of pre-warmed (37 °C) paraformaldehyde (PFA, 4% in PBS) and 0.25% glutaraldehyde, followed by permeabilization in 0.05% Triton-X 100/PBS for 40 s. Fixed and permeabilized samples were then extensively washed and subjected to phalloidin-staining (Atto 488-labeled, AD488-81 or Atto 550-labeled, AD 550-8, stock diluted 1:50 into PBS, ATTO-TEC GmbH, Siegen, Germany). DNA was visualized with 40,6-diamidino-2-phenylindole (DAPI) (Sigma-Aldrich, 1:1000). This was followed by embedding and image acquisition. For the EGFP-mDia2-ΔDAD-expressing cells shown in and quantified for Supplementary Fig. 1, fixations were done with pre-warmed paraformaldehyde (PFA, 4% in PBS), followed by permeabilization in 0.05% Triton-X 100/PBS for 40 s and counterstaining with Atto 594-phalloidin (AD 594-81, ATTO-TEC GmbH, Siegen, Germany, stock-diluted 1:100 into PBS).

For immunolabeling, cells were treated in an identical fashion, except that fixation was done with 4% PFA alone and that the staining procedure was initiated with 5% horse serum dissolved in 1% BSA/PBS followed by primary and secondary antibodies diluted in 1% BSA/PBS, except for primary undiluted supernatants. Additional exceptions were anti-VASP and anti-CP staining, for which a mixture of 4% PFA and picric acid (added to app. 0.7‰) and 3% glyoxal at pH 5.0 (ref. ^77^.) were used, respectively. Primary antibodies were: Anti-Arp2/3 complex (anti-ArpC5, clone 323H3, homemade, undiluted supernatant, validated by CRISPR-KO)^78^; polyclonal anti-Mena, -VASP and -WAVE2 used at 1:100, 1:400 and 1:1000 dilutions, respectively, validated by specific subcellular localization and for VASP and Mena, CRISPR-KO^56^; anti-Lpd (3917, 1:200, validated by siRNA)^79^; monoclonal anti-CP α1/α2 (mAb B5 12.3, 1:4, Developmental Hybridoma Bank, University of Iowa, IA, USA, validated by specific subcellular localization); monoclonal anti-Abi (W8.3, hybridoma supernatant, 1:20, validated by siRNA)^80^. Secondary antibodies were Alexa Fluor^TM^ 594-conjugated Goat anti-Mouse IgG (H + L) (A11032, ThermoFisher Scientific) and Goat anti-Rabbit IgG (H + L) (A11037, ThermoFisher Scientific) used at concentrations according to manufacturer’s instructions. Image acquisition using epifluorescence microscopy was routinely done on an inverted microscope (Axiovert 100TV or Axio Observer, Carl Zeiss, Jena) equipped with 1.3NA 40x or 1.4NA 63x and 100x objectives, widefield illumination (HXP120, Zeiss or DG4, Sutter Instrument) and a Coolsnap-HQ2 camera (Photometrics) controlled by Metamorph (Molecular Devices) or VisiView® software (Visitron Systems GmbH, Puchheim, Germany). Images shown in Supplementary Fig. 5 were captured with a Zeiss confocal LSM 980 with Airyscan 2 equipped with a Plan-Apochromat 63×/1.46 NA oil DIC or C-Apochromat 40×/1.20 W Korr objective using 405 and 561-nm laser lines. Emitted light was detected in the wavelength ranges of 410 to 473 and 576 to 610 nm, respectively.

Pfn1-KO cells transfected overnight with Halo-tagged Pfn1 rescue constructs as shown in Fig. 6 were seeded onto laminin-coated coverslips as before and next day, stained with 1 µM HaloTag TMR (HY-D2270, MedChemExpress, South Brunswick Township, New Jersey, USA) for 15 min, followed by three washes and a 1 h-incubation in full growth medium. Subsequently, cells were fixed and stained for ArpC5 and VASP staining protocols as described above, except that the actin cytoskeleton was counter-stained with Atto 647-labeled phalloidin (AD 647-71, ATTO-TEC GmbH, Siegen, Germany, 1:400). Subsequent imaging was done on a Ti2 Eclipse inverted microscope (Nikon, Düsseldorf, Germany) in widefield mode, using a PCO edge 4.2LT sCMOS camera (Excelitas PCO GmbH, Kelheim, Germany) and an Apo TIRF 100× oil/NA 1.45 objective (Nikon). Fluorophores were excited using 470 nm, 550 nm, and 635 nm LED excitation (pE4000, CoolLED Ltd, Andover, UK) in combination with filter sets as follows: GFP/Atto488 (Ex 480/40, DM 505, Em 545/50), Cy3/TMR (Ex 532/40, DM 512, Em 590/50), Cy5/Atto647 (Ex 637.5/25, DM 625, Em peak 670); abbreviations, Ex: Excitation, DM: Dichroic Mirror, Em: Emission. All hardware components were controlled by NIS-Elements software (Nikon).

Quantitation of lamellipodial F-actin or other lamellipodial factors were done as follows: For F-actin, average pixel intensities of three randomly chosen lamellipodial regions from phalloidin-stained samples lacking microspikes were measured followed by subtraction of a nearby, extracellular region defined as background (for representative regions, see Fig. 1J). The arithmetic mean of these three regions per cell corresponded to a single data-point in measurements displayed as superplot data, as e.g., in Fig. 2B. All other lamellipodial components were measured in an analogous fashion, but considering their specific localizations, i.e., localization throughout the network (in case of F-actin or Arp2/3 complex) *versus* lamellipodial tip components (e.g., VASP, WRC subunits, Lpd). Microspike intensities were quantified in an analogous fashion, but now measuring respective fluorescence intensities throughout microspike bundles.

Lamellipodial width was quantified from phalloidin-stained images with MetaMorph by drawing a line from the lamellipodium tip to the distal edge of the lamella, followed by measuring line lengths. Again, three randomly chosen, lamellipodial sections were measured, averaged and expressed as single data point/cell in μm. Microspike lengths were determined and displayed in an analogous fashion.

In order to assess the frequency of filopodia formation upon expression of constitutively active FMNL3 variants in fixed cells shown in Supplementary Fig. 1 or spontaneous filopodia formation in distinct conditions in Supplementary Fig. 2, cells were manually categorized to harbor or lack filopodia (yes/no) at their cell peripheries based on the morphological categorizations illustrated by exemplary images shown in Supplementary Figs. 1B and 2D. Cells had to harbor at least one, clearly protruding filopodium-like structure in order be classified as “yes”.

### Random cell migration, high magnification video microscopy and fluorescence recovery after photobleaching (FRAP)

Random cell migration assays were performed precisely as described before^69^, and data analyzed in ImageJ (https://imagej.net/ij/) using the Manual Tracking Plugin as well as the Chemotaxis and Migration Tool (Ibidi).

High magnification time-lapse phase-contrast and fluorescence video microscopy was done on an Axio Observer (Carl Zeiss, Jena) equipped with a DG4 (Sutter Instrument) for fluorescence and a VIS-LED for phase-contrast illumination, a 1.4NA 100x Ph3 oil immersion objective and a back-illuminated, cooled, charge-coupled-device camera (CoolSnap HQ2, Photometrics), all driven by VisiView® Software (Visitron Systems GmbH, Puchheim, Germany). Kymography followed by protrusion rate determination were done as described before^37^, and data displayed as superplots. For FRAP experiments, bleaching was done on the same system with a 405 nm diode laser at 60 mW output power to ensure rapid, near-complete bleaching of EGFP-actin in the lamellipodium. Fluorescence recovery of the lamellipodial network was then recorded at 0.5 Hertz and an exposure time of 300 ms. Rates of lamellipodial network assembly *versus* protrusion and rearward flow were manually read out aided again by kymography.

Characterization of mPfn1 rescue constructs by high magnification video microscopy and filopodial analyses shown in Fig. 6A, B and Supplementary Movie 9 were done as follows. Following co-transfection of Halo-tagged constructs with EGFP-mDia2-ΔDAD and seeding onto laminin-coated, 3.5 cm glass bottom dishes (µ-dish 35 mm, high Glass Bottom, Ibidi) for at least 3 h, followed by staining with 100 nM HaloTag TMR for 15 min, three washes in warm medium and incubation for another hour in warm growth medium prior to live-cell imaging. Live cell imaging data were recorded with a Nikon Ti2-E microscope, equipped with a stage-top incubator from Okolab (H301, Ottaviano, Italy), a pE-4000 Cool LED light source and excitation and emission filters for GFP HQ and TRITC. Images were acquired using an CFI P-Apo 100x Lambda oil Ph3 1.45 NA objective (Nikon) with exposure times ranging from 50 to 200 ms and a pco.edge 4.2 sCMOS camera (Excelitas Technologies, Alzenau, Germany), controlled with NIS-Elements (Nikon, Duesseldorf, Germany). Movies were recorded with 5 s intervals for 2.5–10 min.

Analysis of rescued filopodia formation were performed on live cells showing both, detectable EGFP-mDia2-ΔDAD and Halo-mPFN1 variant expression. Amounts and widths of filopodia-like structures were counted and measured manually with Fiji ImageJ (https://imagej.net, Bethesda, USA). Peripheral structures were defined as “filopodia-like” based on two criteria: (i) given their appearance resembled filopodia in phase contrast (thin, dynamic, finger-like protrusions) and (ii) these structures were tipped by EGFP-mDia2-ΔDAD signal (Supplementary Movie 9). Movies employed for quantitations of filopodia numbers and widths were recorded on three independent experimental days, with 47–82 individual, analyzed cells per condition and a total number of manually assessed filopodia-like structures ranging between 1029 and 5337.

### Image processing, statistics and reproducibility, and illustrations

Adjustments for brightness and contrast were performed using Metamorph software (Molecular Devices) or ImageJ. Image and data analysis was mostly done with Metamorph, ImageJ or Fiji, and Figures were assembled with GraphPad Prism 8 and Inkscape. All quantitative datasets were routinely repeated three or more independent times, as can be discerned in each case from displayed data, and shown as arithmetic means ± SEMs (error bars), unless indicated otherwise. Non-quantified data, such as blots were representative of at least two (Figs. 1A, 4A, and Supplementary Fig. 14A) or three independent experiments (Figs. 1C, 5A, and Supplementary Figs. 8A, 8E). All statistical tests constituted two-sided multiple or pair-wise comparisons, with specific tests indicated in each case. No data were excluded from the analyses, unless cells were not fully covered within the field of view or for longer periods of time-lapse microscopy and thus non-interpretable according to specific parameters. Aside from exceptions, investigators were not routinely blinded to allocation during experiments and outcome assessment, in most cases because obtained phenotypes were immediately recognized by the assessing scientist. Statistics were done using SigmaPlot 12.0 (Systat Software, Erkrath, Germany) or Origin 2021 (OriginLab), and presented as provided in respective Figure legends. The scheme in Fig. 7 was created in BioRender. Stradal, T. (2026) https://BioRender.com/dkvr9bp. Supplementary Fig. 13 was created in BioRender. Stradal, T. (2026) https://BioRender.com/9ggtwcm, and Fig. M1 (Supplementary Fig. 12) was created in BioRender. Stradal, T. (2026) https://BioRender.com/dp7bidc.

### Mathematical modeling

The model formulates our hypotheses as a system of differential equations subsequently fitted to criteria defined by experiments, as summarized in Fig. 7 and described in detail in Supplementary Fig. 12.

### Reporting summary

Further information on research design is available in the Nature Portfolio Reporting Summary linked to this article.

## Supplementary information

Supplementary Information

Description of Additional Supplementary Files

Supplementary Movie 1

Supplementary Movie 2

Supplementary Movie 3

Supplementary Movie 4

Supplementary Movie 5

Supplementary Movie 6

Supplementary Movie 7

Supplementary Movie 8

Supplementary Movie 9

Reporting Summary

Transparent Peer Review file

## Source data

Source Data

## Data Availability

Source data are provided with this paper, including all precise *p*-values of each statistical comparison mentioned in the figure legends. Source data are organized into six distinct excel-sheets, with file 1 covering the majority of data, and 2–6 displaying raw data for Fig. 6B, Supplementary Figs. 7, 8D, 8H and 11, respectively. All other raw data, including those of images and movies used for quantifications will be provided by Dr. Klemens Rottner (TU/HZI Braunschweig) to any interested individual without restrictions (k.rottner@tu-braunschweig.de or kro@helmholtz-hzi.de).

## Code availability

Custom codes written in C and developed for description of the stationary states of the model in Supplementary Fig. 12 is available through Zenodo open repository (https://zenodo.org), termed ‘prokomodel’, https://doi.org/10.5281/zenodo.22082048; for further information, please contact Dr. Martin Falcke, MDC Berlin, Germany (martin.falcke@mdc-berlin.de).
