# Supplementary material for: Profilin promotes lamellipodium protrusion by tuning the antagonistic activities of capping protein and VASP
Source: Nat Commun. 2026 Sep 16;17:9887. doi: 10.1038/s41467-026-77694-8 (PMC13582894; doi:10.1038/s41467-026-77694-8)
Supplement: Supplementary file 1 [file 41467_2026_77694_MOESM12_ESM.pdf]

Corresponding author(s): Martin Falcke and Klemens Rottner

Last updated by author(s): 07/25/2026

Reporting Summary

Nature Portfolio wishes to improve the reproducibility of the work that we publish. This form provides structure for consistency and transparency in reporting. For further information on Nature Portfolio policies, see our Editorial Policies and the Editorial Policy Checklist.

Statistics

For all statistical analyses, confirm that the following items are present in the figure legend, table legend, main text, or Methods section.

n/a	Confirmed	
☐	☒	The exact sample size (*n*) for each experimental group/condition, given as a discrete number and unit of measurement
☐	☒	A statement on whether measurements were taken from distinct samples or whether the same sample was measured repeatedly
☐	☒	The statistical test(s) used AND whether they are one- or two-sided<br>*Only common tests should be described solely by name; describe more complex techniques in the Methods section.*
☒	☐	A description of all covariates tested
☐	☒	A description of any assumptions or corrections, such as tests of normality and adjustment for multiple comparisons
☐	☒	A full description of the statistical parameters including central tendency (e.g. means) or other basic estimates (e.g. regression coefficient) AND variation (e.g. standard deviation) or associated estimates of uncertainty (e.g. confidence intervals)
☐	☒	For null hypothesis testing, the test statistic (e.g. *F*, *t*, *r*) with confidence intervals, effect sizes, degrees of freedom and *P* value noted<br>*Give P values as exact values whenever suitable.*
☒	☐	For Bayesian analysis, information on the choice of priors and Markov chain Monte Carlo settings
☒	☐	For hierarchical and complex designs, identification of the appropriate level for tests and full reporting of outcomes
☒	☐	Estimates of effect sizes (e.g. Cohen's *d*, Pearson's *r*), indicating how they were calculated

Our web collection on statistics for biologists contains articles on many of the points above.

Software and code

Policy information about availability of computer code

Data collection	The stationary states of the model described in Supplementary Figure 12 have been determined using custom code in C, which is available through zenodo open repository (https://zenodo.org), termed ‘prokomodel’, DOI 10.5281/zenodo.22082048.<br>Microscopy images were collected using VisiView® Software (Visitron Systems GmbH, Puchheim, Germany), NIS-Elements (Nikon, Duesseldorf, Germany) or Zeiss ZEN software (Carl Zeiss, Jena, Germany).
Data analysis	Analyses of microscopy images: Metamorph (Molecular Devices), ImageJ or Fiji.<br>Figures were assembled with GraphPad Prism 8 and Inkscape.<br>Statistics were done using SigmaPlot 12.0 (Systat Software, Erkrath, Germany) or Origin 2021 (OriginLab).<br>The schemes were drawn using BioRender (www.biorender.com).

For manuscripts utilizing custom algorithms or software that are central to the research but not yet described in published literature, software must be made available to editors and reviewers. We strongly encourage code deposition in a community repository (e.g. GitHub). See the Nature Portfolio guidelines for submitting code & software for further information.

Data

Policy information about availability of data

All manuscripts must include a data availability statement. This statement should provide the following information, where applicable:

- Accession codes, unique identifiers, or web links for publicly available datasets
- A description of any restrictions on data availability
- For clinical datasets or third party data, please ensure that the statement adheres to our policy

All raw data of all all experimental datasets are available from Klemens Rottner (kro@helmholtz-hzi.de) upon request.

Research involving human participants, their data, or biological material

Policy information about studies with human participants or human data. See also policy information about sex, gender (identity/presentation), and sexual orientation and race, ethnicity and racism.

Reporting on sex and gender	N/A
Reporting on race, ethnicity, or other socially relevant groupings	N/A
Population characteristics	N/A
Recruitment	N/A
Ethics oversight	N/A

Note that full information on the approval of the study protocol must also be provided in the manuscript.

Field-specific reporting

Please select the one below that is the best fit for your research. If you are not sure, read the appropriate sections before making your selection.

☒ Life sciences ☐ Behavioural & social sciences ☐ Ecological, evolutionary & environmental sciences

For a reference copy of the document with all sections, see nature.com/documents/nr-reporting-summary-flat.pdf

Life sciences study design

All studies must disclose on these points even when the disclosure is negative.

Sample size	Sample size was determined empirically and dependent experimental capacity. For instance, sample sizes in case of fixed cell analyses were distinct from difficult to acquire high resolution video microscopy data such as FRAP. No statistical tests to predetermine sample sizes were employed, but instead, established practices considered to be manageable in reasonably time frames in the field were used. In all experiments, the chosen number of cells was sufficiently large to yield mean values for each experimental replicate that were very similar.
Data exclusions	No data were excluded from analyses in order to avoid any intended or unadvertent bias, unless data were obtained upon apparent, experimental mistake.
Replication	All biochemical and cell biological measurements were repeated multiple times, at least thrice but frequently even more often, as indicated in all Figure legends, to ensure the same conclusions hold true between repeats. All attempts, excluding those with human mistakes, were successful.
Randomization	For assessment of lamellipodia or filopodia formation, cells were randomly selected from large data sets. All cells that passed quality control were analyzed equally with no sub-sampling and thus, there was no requirement for randomization. The same was true for analyzing filopodia frequencies or morphological or dynamic parameters of both filoppodia and lamellipodia from high magnification video microscopy data. In case of low magnification random cell migration, all cells that were observable within given fields of view for sufficient amounts of time were analyzed, so again, no sub-sampling was undertaken.
Blinding	The experimental origin of cell images was blinded to the investigator assessing the efficiency of lamellipodia or filopodia formation whenever practically possible, but this was frequently useless due to the severity of phenotypes, so the absence of active formin-induced filopodia formation upon profilin loss of function or the lack lamellipodia in Arp2-KO cells cannot seriously or efficiently be blinded to the investigator quantifying respective phenotypes.

Reporting for specific materials, systems and methods

We require information from authors about some types of materials, experimental systems and methods used in many studies. Here, indicate whether each material, system or method listed is relevant to your study. If you are not sure if a list item applies to your research, read the appropriate section before selecting a response.

Materials & experimental systems

n/a	Involved in the study
☐	☒ Antibodies
☐	☒ Eukaryotic cell lines
☒	☐ Palaeontology and archaeology
☒	☐ Animals and other organisms
☒	☐ Clinical data
☒	☐ Dual use research of concern
☒	☐ Plants

Methods

n/a	Involved in the study
☒	☐ ChIP-seq
☒	☐ Flow cytometry
☒	☐ MRI-based neuroimaging

Antibodies

Antibodies used	Primary antibodies for Western Blotting:<br>polyclonal anti-profilin1 raised against the full length protein (Pfn1, 1:1000, PMID: 9463375);<br>polyclonal anti-Pfn1 raised against the N-terminus (Pfn1-N, P7749, 1:3000, Sigma-Aldrich);<br>polyclonal anti-Pfn1 raised against the C-terminus (Pfn1-C, P7624, 1:3000, Sigma-Aldrich);<br>monoclonal anti-Pfn2 (sc-100955, 1:200, Santa Cruz Animal Health);<br>polyclonal anti-VASP (5500-A, 1:2000, PMID: 32391788);<br>rabbit monoclonal anti-Arp2 (ab129018, 1:2000, Abcam);<br>monoclonal anti-GAPDH (MAB374, 1:10000, MERCK);<br>monoclonal anti-HSP70 (also known as HSPA8/HSC70, sc-7298, 1:10000, Santa Cruz Animal Health)<br><br>Primary antibodies for immunolabeling:<br>Anti-Arp2/3 complex (anti-ArpC5, clone 323H3, undiluted supernatant, PMID: 12194823);<br>polyclonal anti-Mena (1:100, PMID: 32391788);<br>polyclonal anti-VASP (1:400, PMID: 32391788);<br>polyclonal anti-WAVE2 (1:1000, PMID: 32391788);<br>polyclonal anti-Lpd (3917, 1:200, PMID: 15469845);<br>monoclonal anti-capping protein alpha1/alpha2 (mAb B5 12.3, 1:4, Developmental Hybridoma Bank, University of Iowa, IA, USA);<br>monoclonal anti-Abi (W8.3, hybridoma supernatant, 1:20, PMID: 15048123)
Validation	Primary antibodies used in Western Blotting and specific for the actin binding protreins Pfn1, Pfn2, VASP and Arp2 were either validated in cited publications previously, or as desribed here, employing CRISPR/Cas9-mediated, specific knockouts for Pfn1, Pfn2, Ena/VASP family members and Arp2.<br>Primary antibodies used for IF were routinely validated by a combination of both, assessing the specificity of their subcellular staining pattern and the absence of specific staining in respective KO cell lines whereever possible, including Ena/VASP family members, Lpd or ArpC5. Details have been specified in the Methods of the main manuscript text.

Eukaryotic cell lines

Policy information about cell lines and Sex and Gender in Research

Cell line source(s)	B16-F1 mouse melanoma wildtype cells were purchased from American Type Culture Collection (ATCC, Manassas, VA, Cat.Nr. CRL-6323, sex: male). All other KO-lines used in this study were either generated here, as described (including Pfn1-KO, Pfn2-KO, Arp2-KO, Lpd-KO, Arp2+Pfn1-KO, Ena/VASP+Pfn1-KO, CPbeta+Pfn1-KO) or published previously (PMIDs: 32391788 for Ena/VASP-KOs, 32094266 for Lpd-KO, 34504078 for CPbeta-KO).<br>Dictyostelium discoideum AX2-214 wild type (WT) cells can be derived from Dictybase (http://dictybase.org/StockCenter/StockCenter.html), and PFNI/II double-KO cells were as described (PMID: 7954798).
Authentication	B16-F1 wildtype cells have been authenticated by local authorities to be mycoplasma-free, of murine origin and to lack any unexpected sequences, such as commonly used antibiotic resistance genes or viral sequences.<br>All cell lines in the lab including derived, single-clone KO lines are routinely subjected to regular mycoplasma-screenings, and experiments are performed on mycoplasma-free cells only.<br>All KO cell lines are authenticated to constitute bona-fide knockouts by confirming the loss of respective gene product by Western blotting and the identity of disrupted alleles by specific gene locus sequencing.<br>Dictyostelium WT and Pfn1/2-KOs have been authenticated as described (PMIDs 26167517 and 7954798)
Mycoplasma contamination	All cell lines in the lab including derived, single-clone KO lines are routinely subjected to regular mycoplasma-screenings, and only cell populations confirmed to be mycoplasma-free have been used for further experiments.

Commonly misidentified lines (See ICLAC register)	No commonly misidentified cell lines have been used in this study.

Plants

Seed stocks	N/A
Novel plant genotypes	N/A
Authentication	N/A
