# Supplementary material for: Profilin promotes lamellipodium protrusion by tuning the antagonistic activities of capping protein and VASP
Source: Nat Commun. 2026 Sep 16;17:9887. doi: 10.1038/s41467-026-77694-8 (PMC13582894; doi:10.1038/s41467-026-77694-8)
Supplement: Supplementary file 2 [file 41467_2026_77694_MOESM13_ESM.pdf]

nature portfolio

Peer Review File

Profilin promotes lamellipodium protrusion by tuning the antagonistic activities of CP and VASP

Corresponding Author: Professor Klemens Rottner

This file contains all reviewer reports in order by version, followed by all author rebuttals in order by version.

Version 0:

Reviewer comments:

Reviewer #1

(Remarks to the Author)
In the manuscript "Profilin promotes lamellipodium protrusion by tuning the antagonistic activities of CP and VASP," Tang et al. present a comprehensive investigation of the interplay between profilin, Ena/VASP, capping protein (CP), and the Arp2/3 complex in regulating lamellipodial actin dynamics. By combining CRISPR/Cas9-mediated knockouts in B16-F1 melanoma cells with quantitative microscopy, the authors address conflicting reports in the literature regarding profilin's function. Their results position profilin as a master regulator of lamellipodia formation, demonstrating that it both promotes Arp2/3 complex activity and modulates the antagonism between Ena/VASP and CP. The study also proposes a mechanistic model based on recruitment competition at lamellipodial edges.

This work makes a significant and original contribution by addressing a long-standing debate about profilin's role. The experimental quality is generally excellent, and the findings will be of broad interest to researchers studying the cytoskeleton and cell migration.

However, several points require clarification before publication, as detailed below. Most issues can be addressed through textual revisions, though I also suggest some additional experiments that would strengthen the paper but are not essential for acceptance.

Major points:

The data support a mutually antagonistic relationship between Ena/VASP and CP in lamellipodia: Ena/VASP loss increases CP levels at the lamellipodium, and Ena/VASP deletion can reverse the CP suppression caused by Pfn1-KO. According to this model, Ena/VASP enrichment should inversely correlate with CP levels. The authors demonstrate this using CP-deficient cells, which show robust accumulation of VASP and Mena at lamellipodial tips. However, profilin levels at the leading edge of these cells remain unknown. In vitro studies indicate competition between profilin and CP for barbed end binding (Pernier et al. 2016), raising the possibility that CP-deficient cells may have elevated Pfn1 levels, which could account for the enhanced Ena/VASP accumulation. If profilin level data in CP-KO cells are unavailable, the direct competition between Ena/VASP and CP could be validated by creating a Pfn1-KO/CP-KO double knockout. According to the proposed model, this should further enhance Ena/VASP accumulation.

While knockouts are powerful tools for studying protein function, they have limitations: gene loss may produce unintended effects on other genes and pathways, and provides limited insight into molecular mechanisms. In Pfn1-KO cells, the authors observe reduced total actin but no change in the G-actin/F-actin ratio. This contrasts with observations in other cell lines; for instance, Skruber et al. showed that Pfn1-KO in CAD cells increases the monomeric-to-polymerized actin ratio. Given that B16-F1 cells show no such change, it is important to dissect profilin's molecular role more precisely, particularly regarding altered accumulation of VASP, CP, and Arp2/3 at lamellipodial edges. Rescue experiments would be valuable: reintroducing either wild-type profilin1 or ligand-binding-deficient mutants (described in Bae et al. 2010 and Ding et al. 2009) into Pfn1-KO B16-F1 cells would distinguish profilin's actin-binding function from its ability to interact with polyproline domain-containing proteins. Alternatively, expressing VASP with a mutated polyproline domain that prevents profilin interaction (as in Gau et al. 2019) would directly test profilin's role in VASP accumulation at the lamellipodium.

The mathematical model proposes that Ena/VASP-CP antagonism results primarily from competition for shared membrane

recruitment sites, but the nature of these sites is not discussed. Could the authors provide more speculation about their identity? What are the candidate recruitment platforms? Lamellipodin? Carmil? Other factors?

Minor points:

The paper presents a Pfn1/Arp2 double knockout. To better distinguish the individual contributions of Pfn1 and Arp2 to the double knockout phenotype, data from the single Arp2-KO cell line should be included.

The model assumes WRC recruitment to the lamellipodial membrane occurs solely through diffusion, without considering active recruitment mechanisms. Please comment on this assumption.

Figure S1C lacks scale bars. Additionally, scale bar values are missing from the legends of Figures 2 and S1.

Figure S5 is not referenced in the text. Please add a reference.

In Figure 1E, the grey dots are extremely difficult to see. Please change their color for better visibility.

The Materials and Methods section references the volume of B16-F1 cells for calculating Pfn1 concentration in different cell lines, but this value is neither provided nor sourced from a reference or described methodologically.

Reviewer #2

(Remarks to the Author)
This manuscript by Tang et. al, investigates the coordination of profilin, Arp2/3, capping protein, and ena/VASP proteins in the lamellipodium, more specifically how disruption of profilin expression alters lamellimodia in B16-F1 mouse melanoma cell line. Using a combination of stable knockdowns of different components they develop a hierarchy of relationships involved in remodeling the branched actin network in the lamellipodium. For example, they fund that reduction of profilin 1 reduced actin expression, but did not alter the ratio of F:G actin. However, this resulted in reduced F-actin in lamellipodia, less and shorter microscpikes. This entailed reduced Arp2/3 and CP incorporation into lamellipodia F-actin, and increased ena/VASP and lpd incorporation. However, lamellipodia still depended upon arp2/3 function. In an Ena/VASP and Pfn1 knockout, Arp2/3 was still reduced, but capping protein increased, revealing that arp2/3 depends on pfn1 for recruitment, whereas cp competes with e/v/. overall, the manuscript does a nice job of parsing roles of fundamental proteins in the actin network, that appear to differ from other cell types (i.e. CAD cells). The use of multiple clones is quite rigorous, but there are issues with statistics that reduce the rigor of the study. Overall, each figure repeats the same assays in different knockdown conditions, but would benefit from more examination of actin polymerization and depolymerization more directly.

Statistics are in appropriate for multiple comparisons, cannot perform multiple t-tests, ANOVA with posthoc testing is required, otherwise there are too many type I errors. Are you comparing means of replicates, or pooling all observations, this should be clear in results. Superplots suggest that you are just pooling the means of each replicate. For example in 4G, I think if you compare the means from the 4 experiments, unclear if would have significant change.

Either show the data for FMNL3 or remove the statement. Data not shown is a behavior of the past. The condition that promotes filopodia (plating on laminin) is worth mentioning in results section, for clarity.
Comments about SRF and MRTF should likely be in discussion, not results.

I think the study would benefit with more close examination of actin turnover (polymerization/depolymerization), free barbed ends, retrograde flow measurements.

What is recruiting Ena/VASP and CP if not barbed ends? Particularly in the model? How is membrane binding included in the model. How the model improves understanding of the mechanism is quite unclear as written.

Version 1:

Reviewer comments:

Reviewer #1

(Remarks to the Author)
The authors have satisfactorily addressed my comments.

Reviewer #2

(Remarks to the Author)
Thank you to the Authors for addressing my concerns and comments in your revised manuscript. I believe that you should add the clarification about superplots (and comparing means not individual cell data across replicates) in your methods section. This is a strength of your approach that should influence hearts and minds in the field! Congratulations on a nice informative study.

Open Access This Peer Review File is licensed under a Creative Commons Attribution 4.0 International License, which permits use, sharing, adaptation, distribution and reproduction in any medium or format, as long as you give appropriate credit to the original author(s) and the source, provide a link to the Creative Commons license, and indicate if changes were made.
In cases where reviewers are anonymous, credit should be given to 'Anonymous Referee' and the source.
The images or other third party material in this Peer Review File are included in the article's Creative Commons license, unless indicated otherwise in a credit line to the material. If material is not included in the article's Creative Commons license and your intended use is not permitted by statutory regulation or exceeds the permitted use, you will need to obtain permission directly from the copyright holder.
To view a copy of this license, visit https://creativecommons.org/licenses/by/4.0/

<u>Detailed point-by-point reply to reviewers' comments:</u> Manuscript **NCOMMS-25-58436**

For your convenience, original reviewer text sections are kept in *Italic*, and our respective author responses are provided in **bold**.

<u>Reviewer 1:</u>

In the manuscript "Profilin promotes lamellipodium protrusion by tuning the antagonistic activities of CP and VASP," Tang et al. present a comprehensive investigation of the interplay between profilin, Ena/VASP, capping protein (CP), and the Arp2/3 complex in regulating lamellipodial actin dynamics. By combining CRISPR/Cas9-mediated knockouts in B16-F1 melanoma cells with quantitative microscopy, the authors address conflicting reports in the literature regarding profilin's function. Their results position profilin as a master regulator of lamellipodia formation, demonstrating that it both promotes Arp2/3 complex activity and modulates the antagonism between Ena/VASP and CP. The study also proposes a mechanistic model based on recruitment competition at lamellipodial edges.

This work makes a significant and original contribution by addressing a long-standing debate about profilin's role. The experimental quality is generally excellent, and the findings will be of broad interest to researchers studying the cytoskeleton and cell migration.

However, several points require clarification before publication, as detailed below. Most issues can be addressed through textual revisions, though I also suggest some additional experiments that would strengthen the paper but are not essential for acceptance.

We thank the reviewer for their appreciation of our manuscript and derived conclusions, and for the thoughtful and interesting suggestions that helped us improve the manuscript. We have attempted to comprehensively provide the requested clarifications, including additional experiments, as detailed in the point-by-point reply below.

Major points:

The data support a mutually antagonistic relationship between Ena/VASP and CP in lamellipodia: Ena/VASP loss increases CP levels at the lamellipodium, and Ena/VASP deletion can reverse the CP suppression caused by Pfn1-KO. According to this model, Ena/VASP enrichment should inversely correlate with CP levels. The authors demonstrate this using CP-deficient cells, which show robust accumulation of VASP and Mena at lamellipodial tips. However, profilin levels at the leading edge of these cells remain unknown. In vitro studies indicate competition between profilin and CP for barbed end binding (Pernier et al. 2016), raising the possibility that CP-deficient cells may have elevated Pfn1 levels, which could account for the enhanced Ena/VASP accumulation. If profilin level data in CP-KO cells are unavailable, the direct competition between Ena/VASP and CP could be validated by creating a Pfn1-KO/CP-KO double knockout. According to the proposed model, this should further enhance Ena/VASP accumulation.

This is an interesting point and we agree with the reviewer that the cited study (Pernier at el., 2016, MPID: 26812019) adds to the complexity of interactions between core machinery components at lamellipodia tips. However, as mentioned by the reviewer, data on Pfn1 localization are not available, for technical reasons, as follows: First, EGFP-tagged variants have failed to reliably localize at sites of dynamic, Arp2/3 complex- and Ena/VASP-driven actin assembly (see e.g. Geese et al., 2000, PMID: 10725224) and no antibodies have been published to the best of our knowledge that would reliably report on Pfn1 accumulation at lamellipodial leading edges, likely because its interactions at these sites are too transient. We also would not expect that potentially enhanced Pfn1 in CP nulls should result in enhanced Ena/VASP accumulation, because we have seen the opposite in our experiments, i.e. enhanced leading edge VASP intensities upon elimination of Pfn1 expression.

Nevertheless, we appreciate the reviewer's suggestion to examine the consequences of combined CP and Pfn1 removal. Before performing these experiments, we used our mathematical model to predict how additional Pfn1 loss would affect leading edge VASP in CP KO cells. Interestingly, the model predicted no additive effect, and this prediction was subsequently confirmed in the new CP/Pfn1 double knockout cell lines (see novel Fig. S12).

We have revised the main text to describe these modelling efforts more fully and have updated former Fig. 5E, now Fig. 6E, following the addition of a new data figure– (see below). We have also added the generation of our new CP+Pfn1 DKO cell lines in Methods, main text and supplementary information (new Fig. S12).

We thank the reviewer for this insightful suggestion, which further supports the validity of our modeling framework and strengthens our overall conclusions.

While knockouts are powerful tools for studying protein function, they have limitations: gene loss may produce unintended effects on other genes and pathways, and provides limited insight into molecular mechanisms. In Pfn1-KO cells, the authors observe reduced total actin but no change in the G-actin/F-actin ratio. This contrasts with observations in other cell lines; for instance, Skruber et al. showed that Pfn1-KO in CAD cells increases the monomeric-to-polymerized actin ratio. Given that B16-F1 cells show no such change, it is important to dissect profilin's molecular role more precisely, particularly regarding altered accumulation of VASP, CP, and Arp2/3 at lamellipodial edges. Rescue experiments would be valuable: reintroducing either wild-type profilin1 or ligand-binding-deficient mutants (described in Bae et al. 2010 and Ding et al. 2009) into Pfn1-KO B16-F1 cells would distinguish profilin's actin-binding function from its ability to interact with polyproline domain-containing proteins. Alternatively, expressing VASP with a mutated polyproline domain that prevents profilin interaction (as in Gau et al. 2019) would directly test profilin's role in VASP accumulation at the lamellipodium.

We fully agree with the reviewer that rescue experiments are highly informative. To address this point, we teamed up with an expert in the field, Dr. Jessica Henty-Ridilla, who previously published tagged Pfn1 constructs that were carefully characterized biochemically. This was important because careful tagging, i.e. with optimized linkers has proven crucial to retain full profilin functionality (Pimm et al., PMID: 35666129).

We first validated the functionality of Halo-tagged, murine, wildtype Pfn1 in our murine, Pfn1-deficient cells, focusing on one of the most prominent Pfn1-loss phenotypes, namely the enhanced formation of mDia2-induced filopodia. In our view, this represents

the cleanest possible rescue experiment. Strikingly, short-term expression of wild-type Pfn1 for 1–2 days robustly rescued this phenotype, whereas polyproline- or actin-binding-deficient Pfn1 mutants were much less effective (see new Fig. 5A, B and new Supplementary Video 9).

Having established functionality of our wildtype rescue construct in rescuing mDia2-induced filopodia formation, we turned to rescuing lamellipodia phenotypes. Again, wildtype, but not the polyproline- or actin binding-deficient mutants rescued the suppression of lamellipodial Arp2/3 complex incorporation. These differences were readily apparent in direct side-by-side comparisons of rescue construct-transfected and non-transfected cells (see new Fig. 5C, D). In contrast, the Pfn1-KO-induced increase in VASP intensities at lamellipodial leading edges were reverted down to half (new Fig. 5C, E) and all these phenotypes were accompanied by at least a partial rescue of lamellipodial actin network intensities (Fig. 5C, F).

We again thank the reviewer for this constructive suggestion. However, we have refrained from adding data on the recruitment of delta-polyPRO VASP, because successful leading edge targeting of VASP lacking its poly-proline domain has been published previously (Loureiro et al., 2002, PMID: 12134088) and was recently confirmed in our lab using EVM triple KO cells (Damiano-Guercio et al., 2020, PMID: 32391788; Karger, Faix and Rottner, unpublished data).

The mathematical model proposes that Ena/VASP-CP antagonism results primarily from competition for shared membrane recruitment sites, but the nature of these sites is not discussed. Could the authors provide more speculation about their identity? What are the candidate recruitment platforms? Lamellipodin? Carmil? Other factors?

In our view, this competitive recruitment mechanism must involve CP- or Ena/VASP interactors probably hooking up to the respective other factor indirectly. Candidates certainly include CARMIL and other factors, as now discussed in additional detail on the pages 21 (bottom) and 22 (top).

Minor points:

The paper presents a Pfn1/Arp2 double knockout. To better distinguish the individual contributions of Pfn1 and Arp2 to the double knockout phenotype, data from the single Arp2-KO cell line should be included.

We have now also included data from single Arp2-KOs, using experiments that mirror those previously performed with Pfn1+Arp2 double-KO cells. Strikingly, lamellipodia were abolished in the absence of functional Arp2/3 complex, irrespective of Pfn1 expression. However, cell migration phenotypes were even more severe upon combined loss of Arp2 and Pfn1. All the new Arp2-KO data are shown in Fig. S7A-D.

The model assumes WRC recruitment to the lamellipodial membrane occurs solely through diffusion, without considering active recruitment mechanisms. Please comment on this assumption.

We have now clarified the corresponding statement in the model description as follows: "WRC arrives at the tip membrane by diffusion and then binds to the membrane. This is

described by the first term of the wm-dynamics below with the rate constant s_{10}" (please see revised Fig. S10).

As stated in the text, we are assuming that the bulk concentration of WRC W_{bulk} is constant. Accordingly, the corresponding term in the dynamics captures both diffusion and binding.

$$\text{binding - dissociation} = k^+F(W_{bulk})(W_T-[wm])-k^-[wm]$$
$$=(k^+F(W_{bulk})+k^-)\left(\frac{k^+F(W_{bulk})W_T}{k^+F(W_{bulk})+k^-}-[wm]\right)=s_{10}(W-[wm])$$
$$s_{10}=k^+F(W_{bulk})+k^-,\quad W=\frac{k^+F(W_{bulk})W_T}{k^+F(W_{bulk})+k^-}$$

We have also clarified that $F(W_{bulk})$ may describe active steps in the recruitment process. Experimental evidence supporting the assumptions that $F(W_{bulk})$ is linear is now mentioned and cited in the revised text accompanying Fig. S10.

Figure S1C lacks scale bars. Additionally, scale bar values are missing from the legends of Figures 2 and S1.

All this has now been corrected, thanks you!

Figure S5 is not referenced in the text. Please add a reference.

Done.

In Figure 1E, the grey dots are extremely difficult to see. Please change their color for better visibility.

Thanks for pointing this out. The grey dots have now been replaced by pink ones that are easier to discern.

The Materials and Methods section references the volume of B16-F1 cells for calculating Pfn1 concentration in different cell lines, but this value is neither provided nor sourced from a reference or described methodologically.

Both the precise cell volume used for the calculation of protein concentration and the original reference for its determination (i.e. Funk et al., 2019, Fig. S1, PMID: 31647411) have now been added to the Methods.

Reviewer #2 (Remarks to the Author):

This manuscript by Tang et. al, investigates the coordination of profilin, Arp2/3, capping protein, and ena/VASP proteins in the lamellipodium, more specifically how disruption of profilin expression alters lamellimodia in B16-F1 mouse melanoma cell line. Using a combination of

stable knockdowns of different components they develop a hierarchy of relationships involved in remodeling the branched actin network in the lamellipodium. For example, they fund that reduction of profilin 1 reduced actin expression, but did not alter the ratio of F:G actin. However, this resulted in reduced F-actin in lamellipodia, less and shorter microscpikes. This entailed reduced Arp2/3 and CP incorporation into lamellipodia F-actin, and increased ena/VASP and lpd incorporation. However, lamellipodia still depended upon arp2/3 function. In an Ena/VASP and Pfn1 knockout, Arp2/3 was still reduced, but capping protein increased, revealing that arp2/3 depends on pfn1 for recruitment, whereas cp competes with e/v/. overall, the manuscript does a nice job of parsing roles of fundamental proteins in the actin network, that appear to differ from other cell types (i.e. CAD cells). The use of multiple clones is quite rigorous, but there are issues with statistics that reduce the rigor of the study. Overall, each figure repeats the same assays in different knockdown conditions, but would benefit from more examination of actin polymerization and depolymerization more directly.

We would like to thank the reviewer for recognition of our efforts and constructive comments, and will respond to specific comments below.

Statistics are in appropriate for multiple comparisons, cannot perform multiple t-tests, ANOVA with posthoc testing is required, otherwise there are too many type I errors.

We thank the reviewer for this comment and agree, so to avoid any potential type I errors, we have now performed ANOVA with posthoc testing throughout the entire manuscript whenever more than two groups have been compared with each other. The precise test used has been provided in each, respective figure legend.

Are you comparing means of replicates, or pooling all observations, this should be clear in results. Superplots suggest that you are just pooling the means of each replicate. For example in 4G, I think if you compare the means from the 4 experiments, unclear if would have significant change.

Please note that, in all cases, our statistical comparisons are based on replicate means (N), not on pooled individual measurements (n). As the reviewer will appreciate, using pooled measurements would artificially inflate statistical power; given the large n, virtually all comparisons would become highly statistically significant.

Either show the data for FMNL3 or remove the statement. Data not shown is a behavior of the past.

Good point. The FMNL3-data are valid and thus now shown in new Fig. S1, using two variants of constitutively active FMNL3.

The condition that promotes filopodia (plating on laminin) is worth mentioning in results section, for clarity.

Done.

Comments about SRF and MRTF should likely be in discussion, not results.

We agree, and the statements have thus been moved from the Results to the Discussion in the revised manuscript version.

I think the study would benefit with more close examination of actin turnover (polymerization/depolymerization), free barbed ends, retrograde flow measurements.

We would like to politely point out that the reviewer might have missed our data on lamellipodial FRAP of cells expressing EGFP-actin in various conditions, which are included in the Supplement. These experiments allow us to determine the total actin assembly rates (i.e. actin network polymerization), as well as indirectly rearward flow, since actin assembly is the sum of protrusion and rearward flow (see Fig. S5C).

We found that all the lamellipodial phenotypes seen, for example in Pfn1 null clones coincided with reduced protrusion (Fig. S5A, B, Supplementary Video 3). This reduction in protrusion again correlated well with decreased lamellipodial actin assembly rates (see Fig. S5C and for quantitation Fig. S5D, Supplementary Video 5).

Analogous experiments were performed for the Ena/VASP+Pfn1 quadruple KO cells lines, see Fig. S8A, B (protrusion) and Fig. S8C, D (actin assembly rate). The only condition in which protrusion, actin network assembly and rearward flow diverged was in the quadruple-KO clone #19, which combined very low protrusion with a slight increase in actin assembly rate (Fig. S8D), which by definition can only be explained by increased rearward flow. We speculate that this may result from defects in lamellipodial adhesion in the additional absence of Ena/VASP family members, which are well established to additionally localize to nascent adhesions or focal complexes behind the leading edge (Rottner et al., 1999, PMID: 10559946; Rottner et al., 2001, PMID: 11598195). However, because this point is tangential to the main message of the manuscript, we did not elaborate on this in further detail.

Finally, we have not been able to determine free barbed end numbers in all of our conditions. The most reliable way to perform such experiments would involve the addition of polymerizable, tagged actin variants to ghosts, i.e. semi-alive cells shortly after gently opening up the membrane (Symons & Mitchison, 1991, PMID: 1860882; Chan et al., 1998, PMID: 9405304), making sure not to remove small filament stubs but allowing conditions for continued, polymerization of free barbed ends. Optimizing conditions in order to obtain fully reliable data from such an approach would be beyond the scope of the current manuscript.

Nevertheless, we indirectly estimated amounts of polymerizable filaments from both, quantifying lamellipodial F-actin from phalloidin stainings (see e.g. Fig. 4K) and the frequency of new filament generation by branching through quantitation of endogenous lamellipodial Arp2/3 complex levels (see Fig. 2). Together, these measurements provided the experimental basis for developing our model.

What is recruiting Ena/VASP and CP if not barbed ends? Particularly in the model? How is membrane binding included in the model. How the model improves understanding of the mechanism is quite unclear as written.

We have now revised the wording in the section '*Modeling reveals competitive membrane recruitment of Ena/VASP and CP as a key control mechanism of lamellipodial protrusion*' to clarify more explicitlyhow the modeling supports the conclusions of our study.

Specifically, we now state more clearly that modelling the Pfn1-KO condition as a reduction in branching rate was sufficient to reproduce the experimentally observed changes in lamellipodial VASP, F-actin, CP, WRC, and Arp2/3 complex accumulation.

We also emphasize that, according to the model, Ena/VASP and CP must compete for shared membrane recruitment sites in order to be able to recapitulate the experimental data. Finally, as described in our response to reviewer 1 above, we have now added a model prediction for the outcome of VASP accumulation in CP+Pfn1 double-KO cells, which was subsequently confirmed experimentally by data shown in new Fig. S12.

More generally, mathematical modelling provides a quantitative formulation of biological ideas and hypotheses. It allowed us to test if existing concepts are sufficient to explain new experimental results, or whether additional hypotheses are required. In our case, the modelling showed that many of our experimental results can be explained by established principles. At the same time, it identified competitive membrane recruitment of CP and Ena/VASP as an additional mechanism required to explain the full dataset.

The competitive recruitment is described by the dynamics for [vm] and [cm] in Fig. S10. [vm] and [cm] constitute Ena/VASP and CP bound to these membrane recruitment sites. There is a limited number m of these sites to which both molecules can bind. The number of free sites is thus m-[vm]-[cm]. Consequently, recruitment of one species reduces the number of available binding sites for the other. If [vm]=m, CP cannot bind any more at all and *vice versa*. As now discussed in greater detail in the revised Discussion, the molecular nature of this competitive recruitment mechanism remains to be determined.
