# Supplementary material for: Profilin promotes lamellipodium protrusion by tuning the antagonistic activities of capping protein and VASP
Source: Nat Commun. 2026 Sep 16;17:9887. doi: 10.1038/s41467-026-77694-8 (PMC13582894; doi:10.1038/s41467-026-77694-8)

SUPPLEMENTARY INFORMATION TO:

Profilin promotes lamellipodium protrusion by tuning the antagonistic activities of capping protein and VASP

Yubo Tang[1,2,§], Matthias Schaks[1,2,%], Xinqi Jiang[1,2], Marius Karger[1,2], Magdalena Mietkowska[1,2,&], Julian Kaltenhäuser[1,2], Jonas Scholz[3], Ruth Benavente-Naranjo[1,2], Sijian Hu[1,2], Sarah Körber[3,#], Zhilun Li[4], Christopher Lambert[1,2,5], Jessica L. Henty-Ridilla[6,7], Theresia E.B. Stradal[8], Roger Karlsson[4], Peter Bieling[9,10], Jan Faix[3], Martin Falcke[11,12*] and Klemens Rottner[1,2,13,*]

[1]Division of Molecular Cell Biology, Institute for Cell- and Neurobiology, Technische Universität Braunschweig, 38106 Braunschweig, Germany; [2]Molecular Cell Biology, Helmholtz Centre for Infection Research, 38124 Braunschweig, Germany; [3]Institute for Biophysical Chemistry, Hannover Medical School, 30625 Hannover, Germany; [4]Department of Molecular Biosciences, The Wenner-Gren Institute, Stockholm University, SE-106 91 Stockholm, Sweden; [5]Department of Microbial Drugs, Helmholtz Centre for Infection Research (HZI), 38124 Braunschweig, Germany; [6]Department of Biochemistry and Molecular Biology, SUNY Upstate Medical University, Syracuse, NY 13210, USA; [7]Department of Neuroscience and Physiology, SUNY Upstate Medical University, Syracuse, NY 13210, USA; [8]Department of Cell Biology, Helmholtz Centre for Infection Research, 38124 Braunschweig, Germany; [9]Department of Systemic Cell Biology, Max Planck Institute of Molecular Physiology, 44227 Dortmund, Germany; [10]Cellular Biophysics Section, Randall Centre for Cell and Molecular Biophysics, King's College London, London, UK; [11]Max Delbrück Center for Molecular Medicine in the Helmholtz Association, 13092 Berlin, Germany; [12]Department of Physics, Humboldt University, 12489 Berlin, Germany; [13]Braunschweig Integrated Centre of Systems Biology (BRICS), Braunschweig, Germany; [§]present address: Division of Life Sciences and Medicine, University of Science and Technology of China, 230026 Hefei, China; [%]present address: Soilytix GmbH, 20354 Hamburg, Germany; [&]present address: Nanoscale Infection Biology, Helmholtz Center for Infection Research HZI, 38124 Braunschweig, Germany; [#]present address: Faculty of Biological and Environmental Sciences and HiLIFE Institute of Biotechnology, University of Helsinki, FI-00014, Helsinki, Finland

[*]Authors for correspondence:

Klemens Rottner, Email: k.rottner@tu-braunschweig.de or kro@helmholtz-hzi.de

Martin Falcke, Email: martin.falcke@mdc-berlin.de

Supplementary Fig. 1

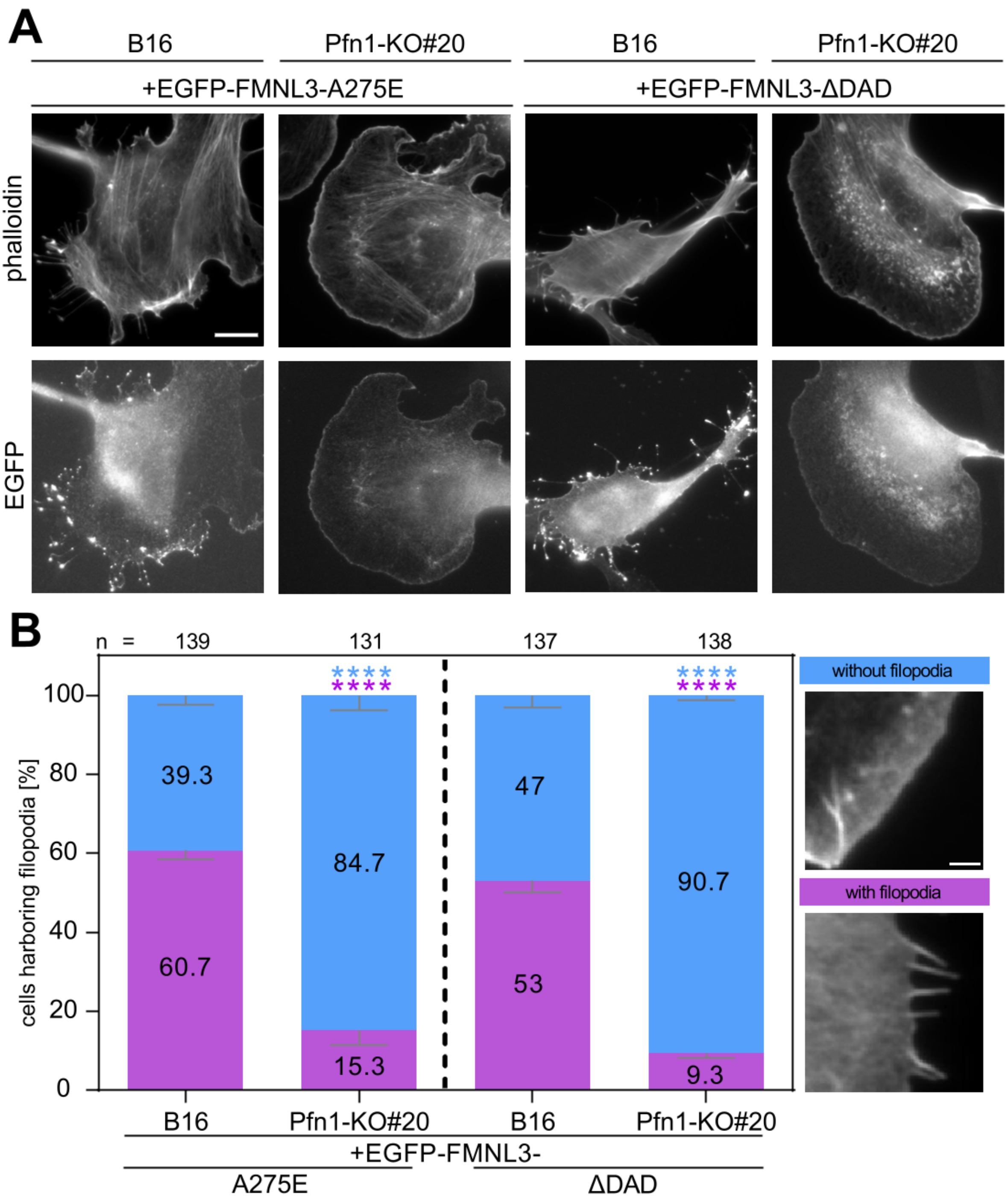

Supplementary Fig. 1. Induction of filopodia by constitutively active FMNL3 variants is strongly abrogated upon loss of Pfn1. (**A**) Representative images of B16-F1 WT or Pfn1-KO #20 cells transiently transfected with EGFP-FMNL3-A275E or EGFP-FMNL3-∆DAD fixed and counterstained with phalloidin, as indicated. Scale bar, 10 µm. (**B**) Quantitation of B16-F1 wildtype or Pfn1-KO #20 cells harboring filopodia (pink) or not (blue) manually categorized upon expression of respective, constitutively active FMNL3-variants, based on morphological features of the cell periphery and judged upon phalloidin staining as illustrated in inset images on the right. n=numbers of individual cells analyzed, scale bar, 2 µm. Color-coded results shown for each pair of independently-generated data for wildtype and Pfn1-KO cells upon expression of respective construct constitute arithmetic means and standard error of means (SEM) from 4 independent experiments. Statistics, two-sided, two-sample T-test, ****p≤0.001.

Supplementary Fig. 2

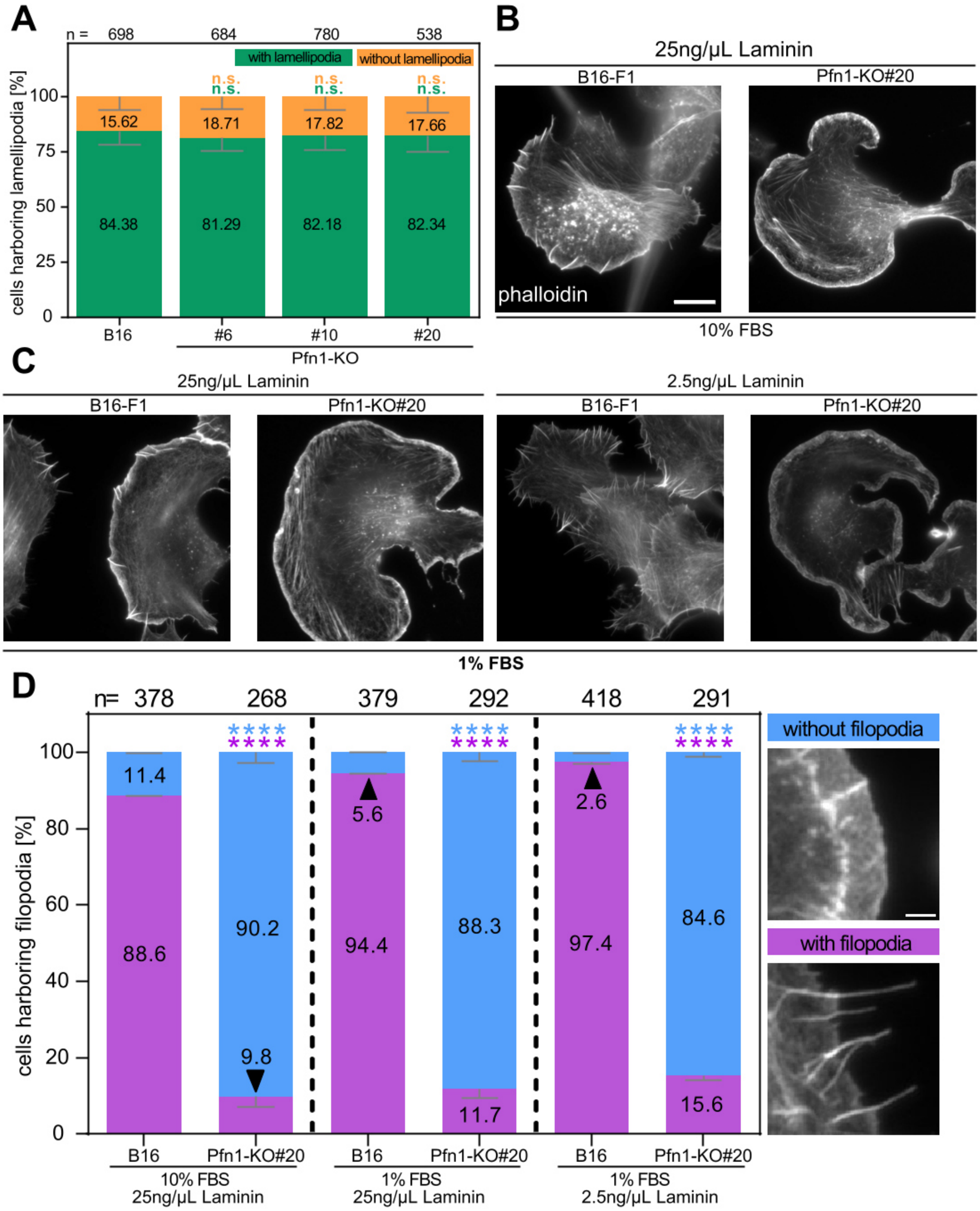

Supplementary Fig. 2. Profilin loss of function abrogates the frequency of spontaneous filopodia, but not lamellipodia formation. (**A**) Quantitation of the percentage of cells capable of forming lamellipodia, for color-code of cells with *versus* without lamellipodia see top right. Bars and numbers show arithmetic means and standard errors of means (SEMs), n=numbers of analyzed cells. Datasets from each individual KO-line were statistically compared to WT using ANOVA with Dunnett's post hoc test; n.s., not significant. (**B**) Representative images of the actin cytoskeleton of wildtype (B16-F1) and Pfn1-KO (clone #20) cells upon seeding on laminin (25 ng/µl) in full growth medium (10% FBS), which constitutes our standard conditions for migration and lamellipodia formation. Under these conditions, most (but not all) peripheral actin bundles appear as microspikes remaining embedded into lamellipodia in control cells, albeit largely missing, however, in Pfn1-KO cells (see independent quantitation of microspike numbers in Fig. 2D). (**C**) Images of WT (B16-F1) and Pfn1-KO (clone #20) cells growing in conditions suboptimal for lamellipodia formation (reduced FBS and/or laminin concentrations, as indicated) and thus known to enhance extent and frequency of microspike or filopodia formation. Left panels display cells in 1% FBS and common laminin (25 ng/µl) concentration and right panels 1% FBS and 2.5 ng/ml laminin concentration. Note explosive filopodia formation of filopodia and microspikes in the latter condition in wildtype, but not Pfn1-KO cells. Scale bar in B is also valid for C, 10 µm. (**D**) Quantitation of filopodia formation in all aforementioned conditions; for color-code of cells with *versus* without filopodia see panels on the right. Although we observed incremental increases in filopodia formation in low serum and laminin concentrations in both genotypes, filopodia formation was still drastically suppressed in all conditions in the absence of Pfn-1. Scale bar in right, illustrative panels, 2 µm. Statistically significant reduction of filopodia formation in the absence of Pfn1 in each case was confirmed by two sample T-test, ****$p \leq 0.001$.

Supplementary Fig. 3

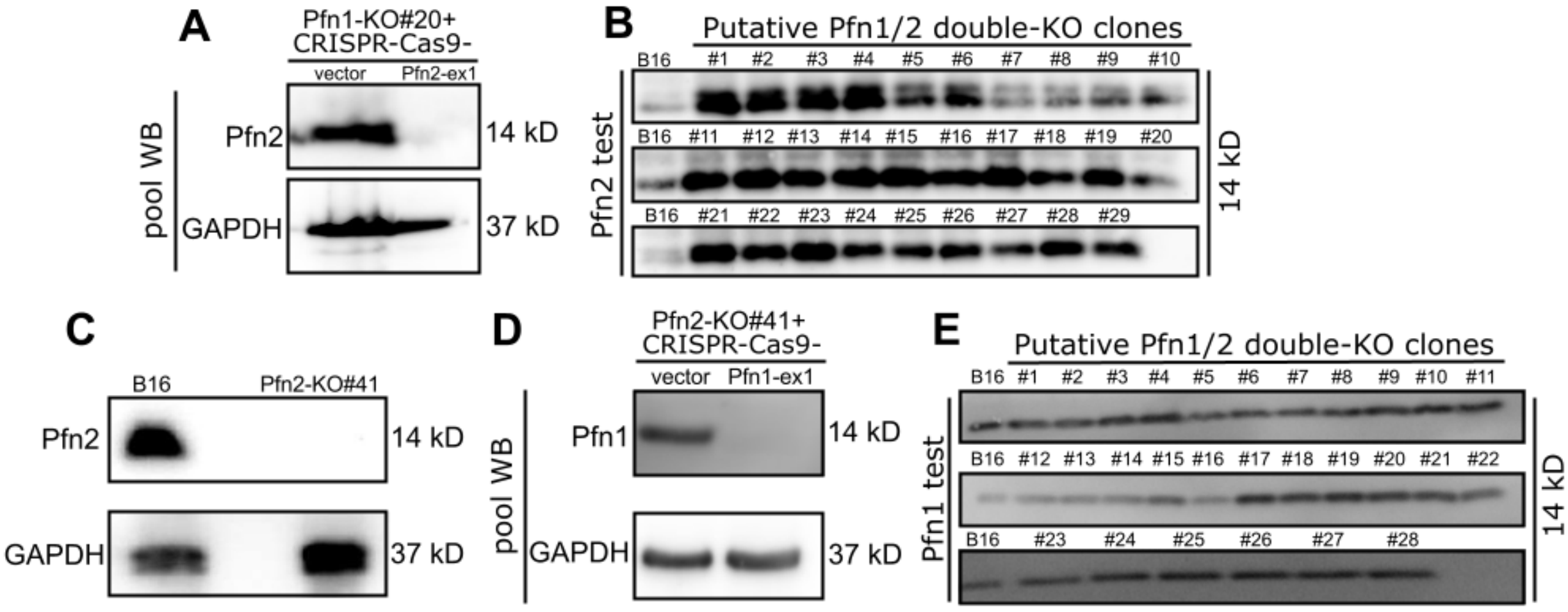

Supplementary Fig. 3. Consecutive genetic disruption of Pfn1 and -2 in mammalian cells does not lead to viable cell progeny. (**A**) Expression of Pfn2 in Pfn1-KO (clone #20) cells before and after acute disruption of the gene by CRISPR, using a Pfn2-specific guide sequence targeting exon 1 (Pfn2-ex1). Note the efficient suppression of Pfn2 expression app. 7 days after transient transfection with Pfn2-specific CRISPR-construct and selection for transfected cells with puromycin. (**B**) Screening Western blot with the aim to select for Pfn2-deficient cell line upon single cell cloning of the cell population shown in A (right). Note that out of 29 single-cell clones of Pfn1-KO#20 isolated and expanded upon acute Pfn2 disruption shown in A, not a single one of them lacked Pfn2 expression. (**C**) Western blot showing the absence of Pfn2 in a B16-F1 clone selected upon CRISPR/Cas9 targeting of Pfn2. (**D**) Expression of Pfn1 in Pfn2-KO (clone #41) cells before and after acute disruption of the gene by CRISPR, using the Pfn1-specific guide targeting exon 1 (Pfn1-ex1). Again, note the efficiency of suppression of Pfn1 expression app. 7 days after transient transfection with Pfn1-specific CRISPR-construct and selection for transfected cells with puromycin. (**E**) Screening Western blot to select for stably Pfn1-depleted lines in the Pfn2-KO background upon single cell cloning of the cell population shown in D (right). 28 clones were analyzed, but all showed Pfn1 expression, indicating that, similar to the results shown in subpanels A and B, clones devoid of both Pfn1 and -2 are not viable or capable of proper cell growth and thus lost during the subcloning procedure.

Supplementary Fig. 4

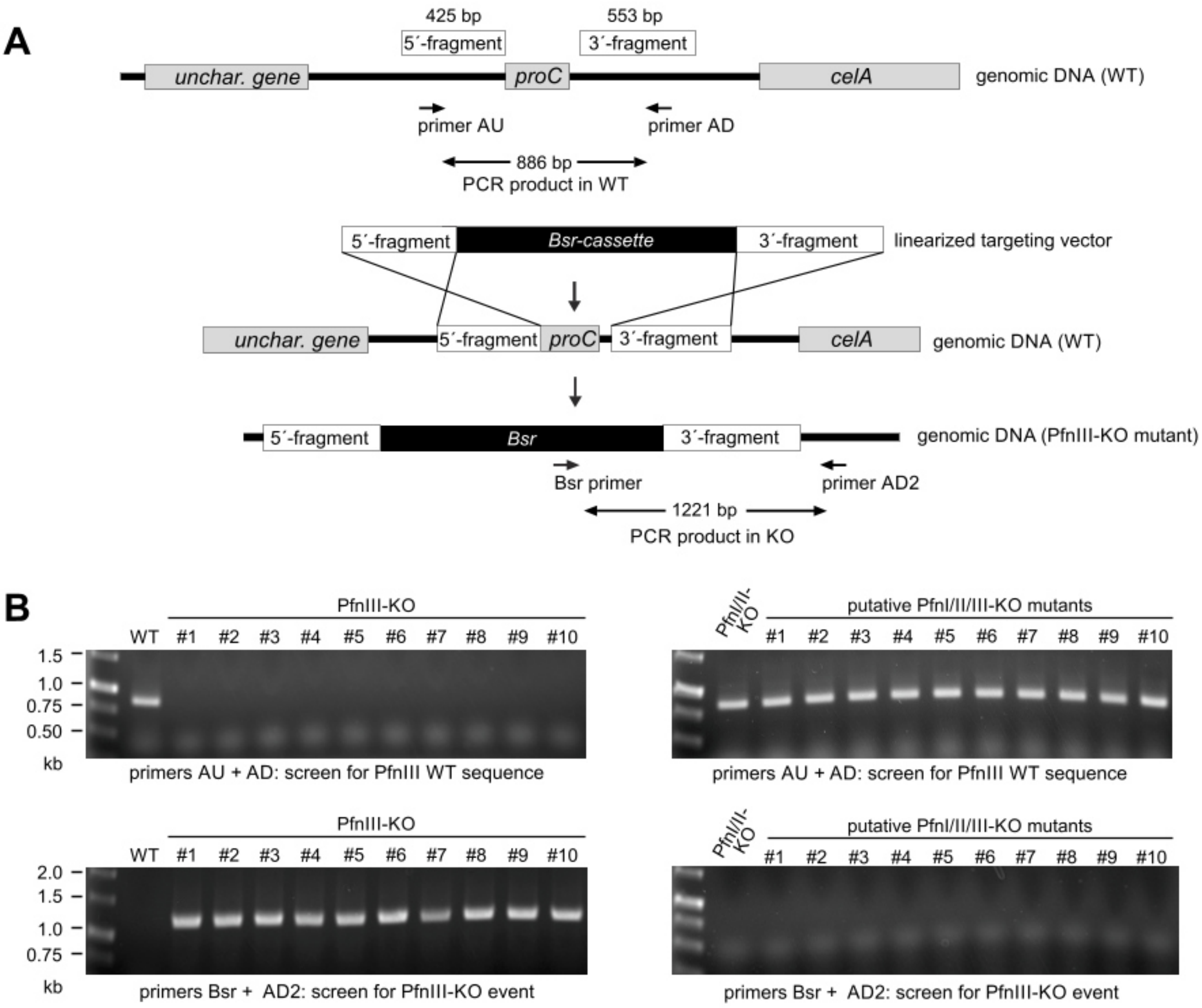

Supplementary Fig. 4. Consecutive genetic disruption of PfnI, -II and -III in *D. discoideum* cells does not give rise to viable cell progeny. (**A**, **B**) ProfilinIII (PfnIII) can be efficiently disrupted in *Dictyostelium* Ax2 WT cells, but not in PFNI/II-KO cells. (**A**) Schematic representation of the organization of the *proC* gene encoding PfnIII, construction of the targeting vector and generation of PfnIII-KO cells. The 5’ and 3’ non-coding sequences flanking the *proC* gene, as indicated, were inserted into pLPBLP containing the Bsr resistance cassette. The linear targeting vector was then used to disrupt the *proC* gene by homologous recombination in Ax2WT and PfnI/II-KO mutant cells. (**B**) Upon homologous recombination, Blasticidin-resistant cells were examined for the presence of an intact or disrupted PfnIII allele by PCR employing two different primer sets. Top panels: the primer combination AU and AD as shown in F specifically identifies the presence of an intact wild-type allele. Bottom panels: the

primer combination Bsr and AD2 as shown in F identifies insertion of the targeting vector into the *proC* gene and successful disruption of the gene. The high efficiency with which PfnIII can be eliminated in WT cells and the inability to inactivate PfnIII in the PfnI/II-KO mutant background strongly suggest an obligatory function of profilin in *D. discoideum* cells.

Supplementary Fig. 5

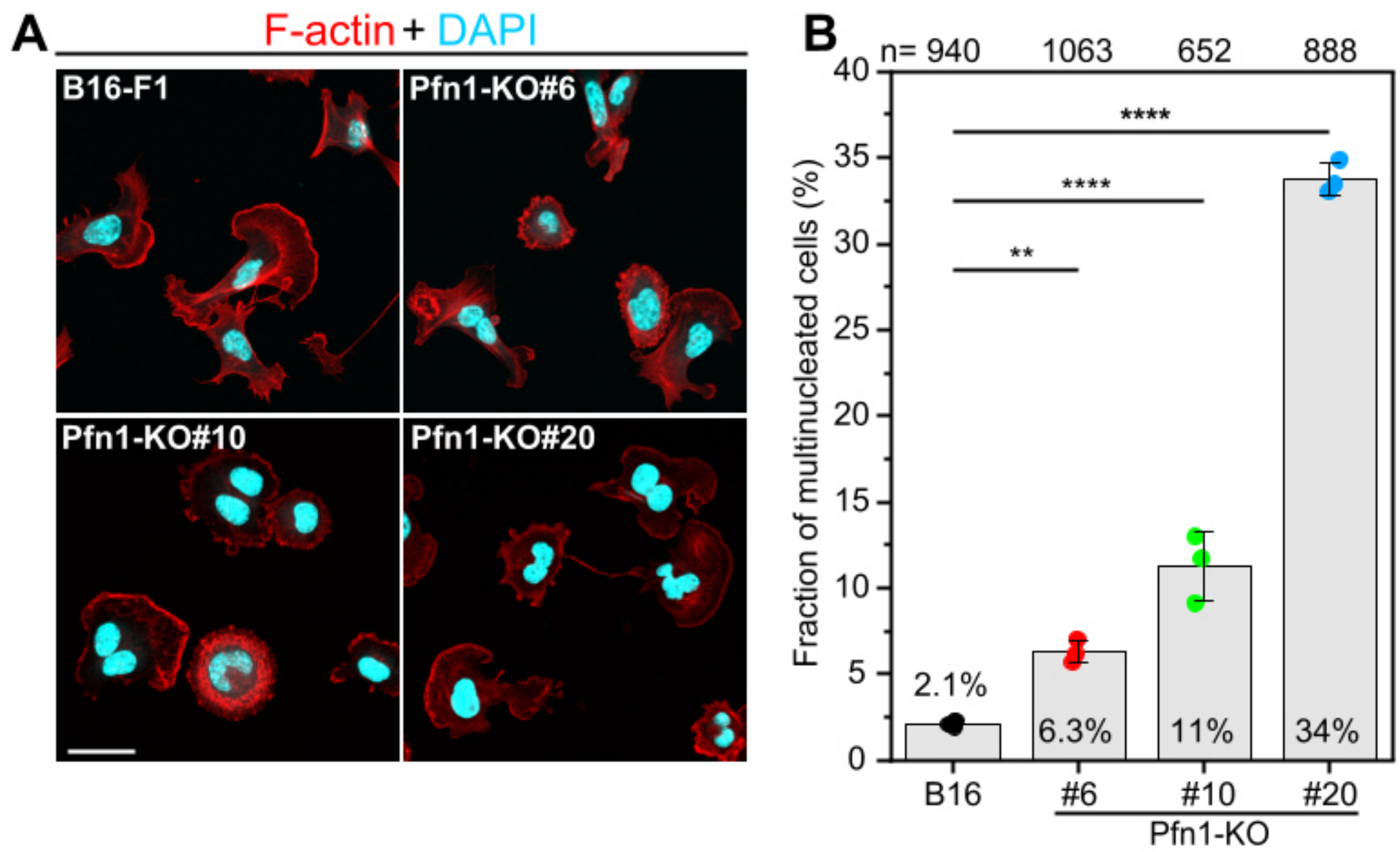

Supplementary Fig. 5. Pfn1-KO cell lines display varying degrees of multinucleation that correlate with remaining levels of Pfn2. (**A**) Representative images of WT (B16-F1) and Pfn1-KO cell lines (#6, #10 and #20), as indicated, fixed and double-stained with phalloidin (red) for the actin cytoskeleton and DAPI (turquois) for nuclear DNA. Scale bar, 20 µm. (**B**) Quantification of the fraction of multinucleated WT and mutant cells as shown in A. Note the incremental increase of multinucleation in Pfn1-KOs, negatively correlating with the residual amounts of Pfn2, and hence overall profilin activity present in these lines (compare Fig. 1E). Bars and error bars represent arithmetic means and standard deviations from three independent experiments (N, colored dots). n, number of cells analyzed. One-way ANOVA and Tukey Multiple Comparison test were used to reveal statistically significant differences between datasets. **$p \leq 0.01$; ****$p \leq 0.001$.

Supplementary Fig. 6

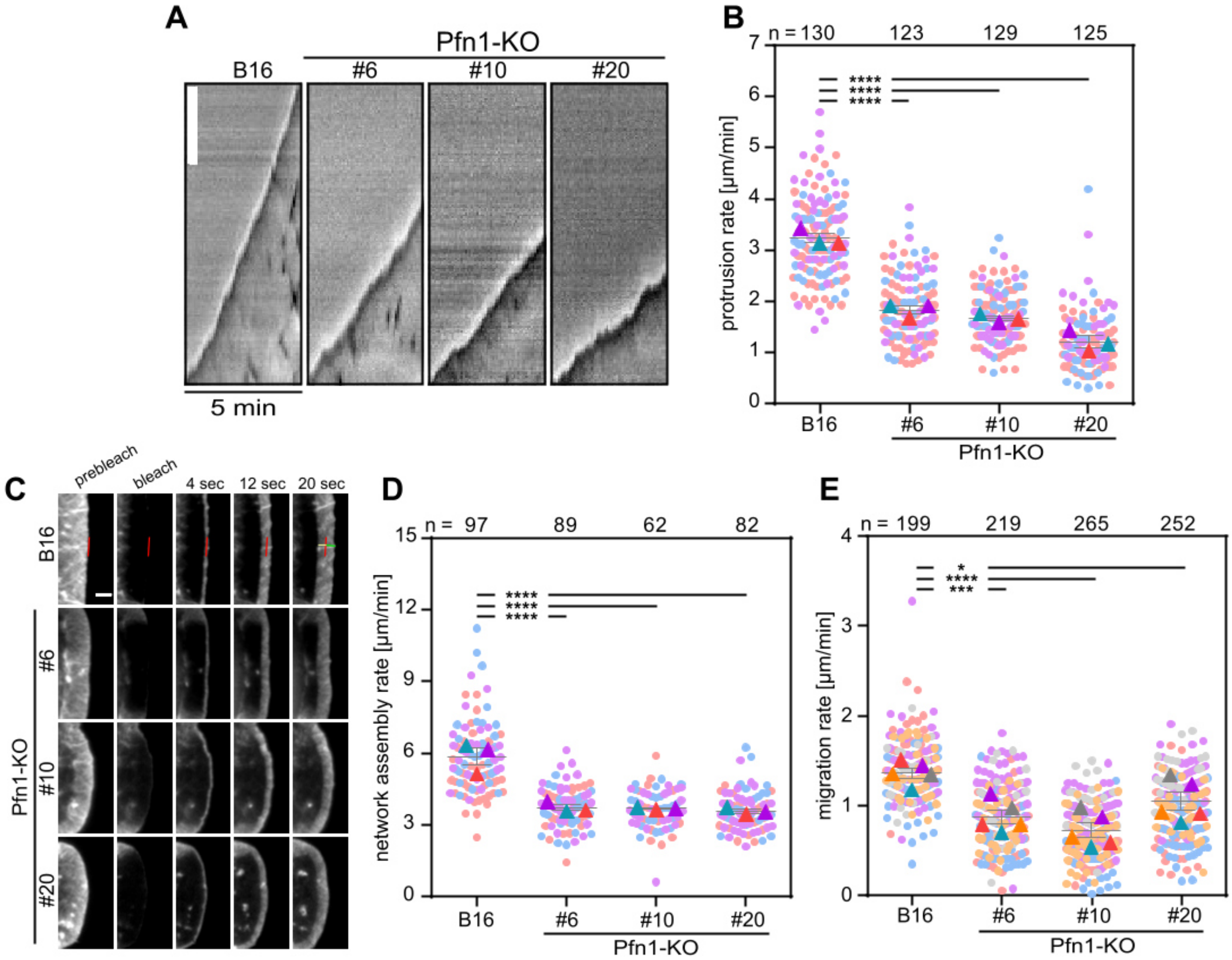

Supplementary Fig. 6. Pfn1-deficient cells display reduced lamellipodial protrusion, actin network assembly and overall random cell migration. (**A**) Kymographs (space-time plots) of representative lamellipodial edges of cells with genotypes as indicated and recorded by time-lapse phase contrast microscopy; time is as provided and white scale bar corresponds to 3 µm. (**B**) Quantitation of lamellipodial protrusion rates determined from kymographs of individual lamellipodia (shown in A) for the different cell types. The protrusion rates determined for each cell line are shown as superplots, displaying measurements from individual cells and their arithmetic means as color-coded dots and triangles, respectively. Grey lines and error bars display means and standard errors of the means (SEMs), n = numbers of measured cells. Arithmetic means of replicates from each individual KO-line

were statistically compared to WT (B16) using ANOVA with Dunnett's post hoc test; ****p≤0.001. (**C**) Fluorescence recovery after photobleaching (FRAP) analysis in lamellipodia of cells transiently expressing EGFP-tagged actin. Fluorescence recovery from the front following the bleach is taken as direct readout of lamellipodium network assembly rate. This equals the dimension of the lamellipodial actin network building up over time (20 seconds in this case, as indicated, see B16 panels) and constitutes the sum of protrusion distance (green line in front of the red line marking the lamellipodial edge in the prebleach frame) and rearward flow distance (yellow distance proximal to the prebleach red line). Scale bar, 2 µm. For the movie corresponding to these data, see Supplementary Movie 5. (**D**) Quantitation of network assembly rate assessed as representatively shown in C. Data are displayed as superplots in analogy to what's described for protrusion rates in B. Statistics as above using ANOVA with Dunnett's post hoc test; ****p≤0.001. (**E**) Quantitation of random migration rates of B16-F1 wildtype (B16) and Pfn1-KO cell lines as indicated. Migration rates were extracted from migration tracks (see below) over time periods of 10 hours. Data are displayed as superplot data as described above; five independent experiments in this case (distinct colors). For a compiled representative movie including all cell lines, see Supplementary Movie 2. For statistics see B; *p≤0.05; ***p≤0.005; ****p≤0.001.

Supplementary Fig. 7

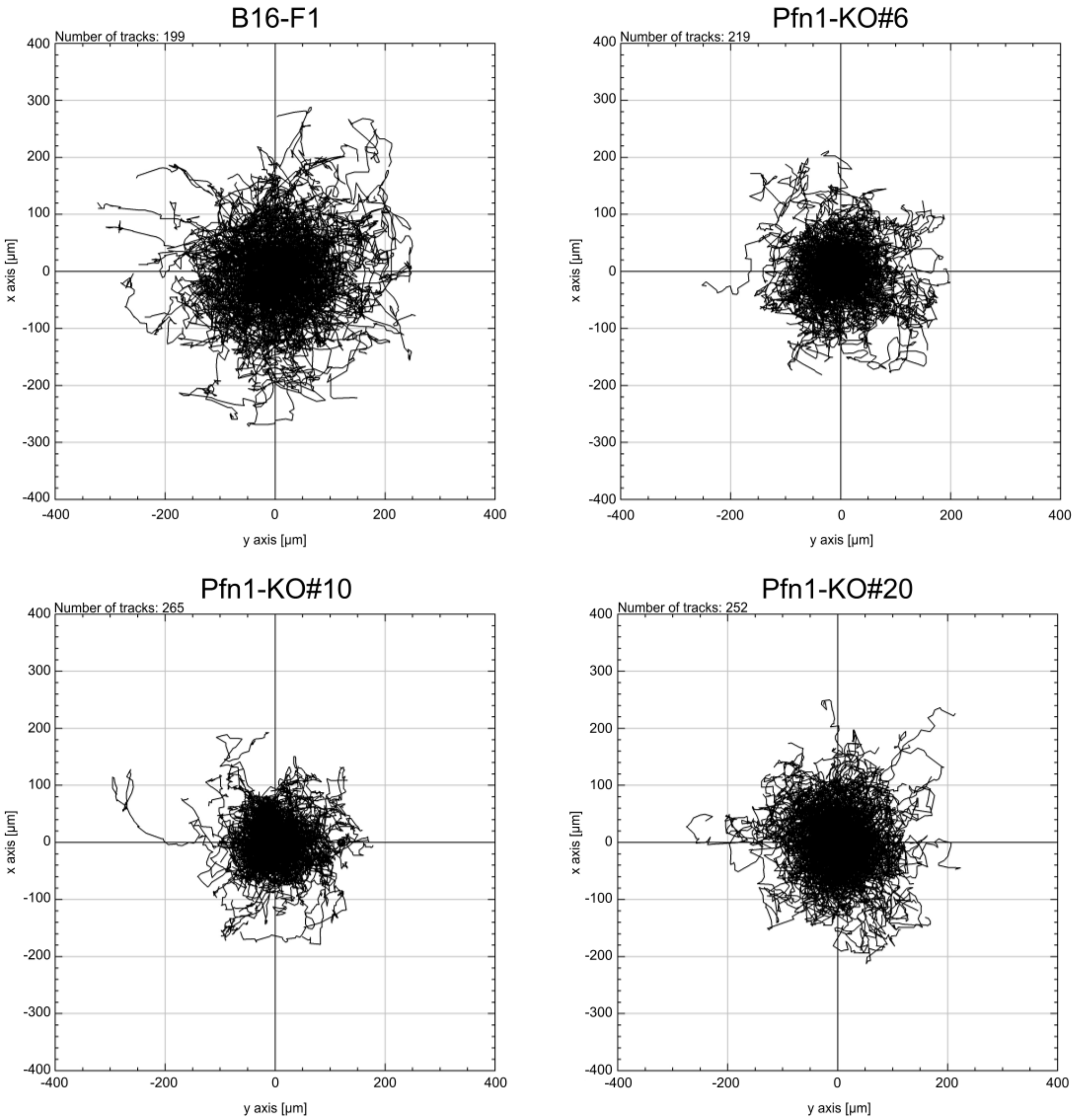

Supplementary Fig. 7. Pfn1-deficient B16-F1 melanoma cells migrate shorter distances than WT cells. Trajectory plots as obtained from random migration movies of distinct cell lines migrating on laminin and exemplarily shown in Supplementary Movie 2. Each line corresponds to an individually tracked cell, the starting point of which was positioned in the center of the coordinate system. Cells were tracked for a maximum of 10 hours. Note the modest size reduction of trajectory plots as compared to wildtype in lines lacking Pfn1, consistent with the reduced migration rates (Supplementary Fig. 6E) as well as reduced lamellipodial protrusion rate (Supplementary Fig. 6B). This fits the notion that

efficient migration of B16-F1 melanoma on laminin strongly correlates with the capability of lamellipodia formation[1, 2].

Supplementary Fig. 8

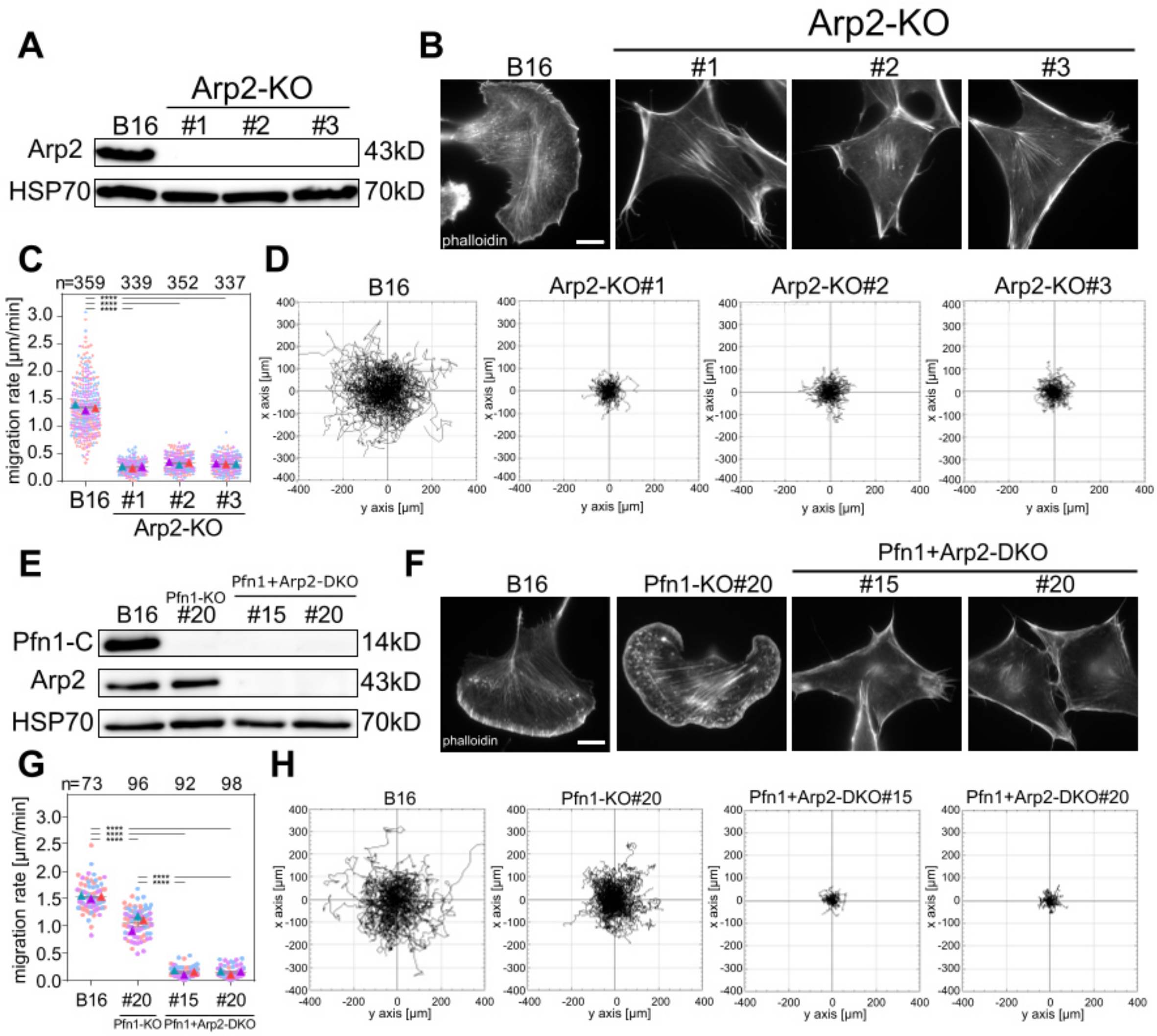

Supplementary Fig. 8. Both wildtype and Pfn1-deficient lamellipodia require Arp2/3 complex.

(**A**) Western blot showing Arp2 expression in B16-F1 wildtype and 3 selected clones following disruption of the single Arp2 gene (Arp2-KO #1, #2, #3). (**B**) Representative phalloidin images in cells with genotypes, as indicated. Scale bar, 10µm. Note that none of the Arp2-deficient cell lines was able to form any structure reminiscent of a lamellipodium, as expected[3, 4, 5]. (**C**, **D**) Random cell migration on laminin of B16-F1 cells lacking lamellipodia due to disruption of the obligatory Arp2 gene. Cells were

tracked from time-lapse movies for a maximum of 10 hours (see Methods). The trajectory plots in D display one representative experiment out of three quantified in C. As before, each track corresponds to an individual cell, the starting point of which was positioned in the center of the coordinate system. Statistics in C: Comparison of arithmetic means of replicates of each KO dataset with WT using ANOVA with Dunnett's post hoc test; ****p≤0.001. (**E**) Western blot showing the removal of Arp2 in Pfn1-KO (clone #20). Two independent Pfn1+Arp2 double-KO (DKO) clones were selected, as indicated, and subjected to further analyses. Note the complete absence of detectable Arp2 staining. (**F**) B16-F1 cells with genotype as indicated seeded and stained for the actin cytoskeleton. Note the complete abrogation of Pfn1-deficient lamellipodia in the absence of Arp2 as well as compromised intracellular actin filament accumulation. Scale bar, 10 µm. (**G**) Quantitation of random cell migration in B16-F1 wildtype (B16) as compared to the parental Pfn1-KO#20 and the same cells upon disruption of the Arp2 gene (clones as above). Note the virtual elimination of migration in cells lacking both Arp2/3 complex activity and Pfn1, the latter in essence translating into lost processive formin activity (see above). For statistics, essentially all experimental groups, i.e. their arithmetic means of replicates, were compared with each other using ANOVA with Tukey's posthoc test; ****p≤0.001. (**H**) Trajectory plots obtained from random migration movies, examples of which can be found in Supplementary Movie 7; everything else as described above for panel D, except for genotypes as indicated.

Supplementary Fig. 9

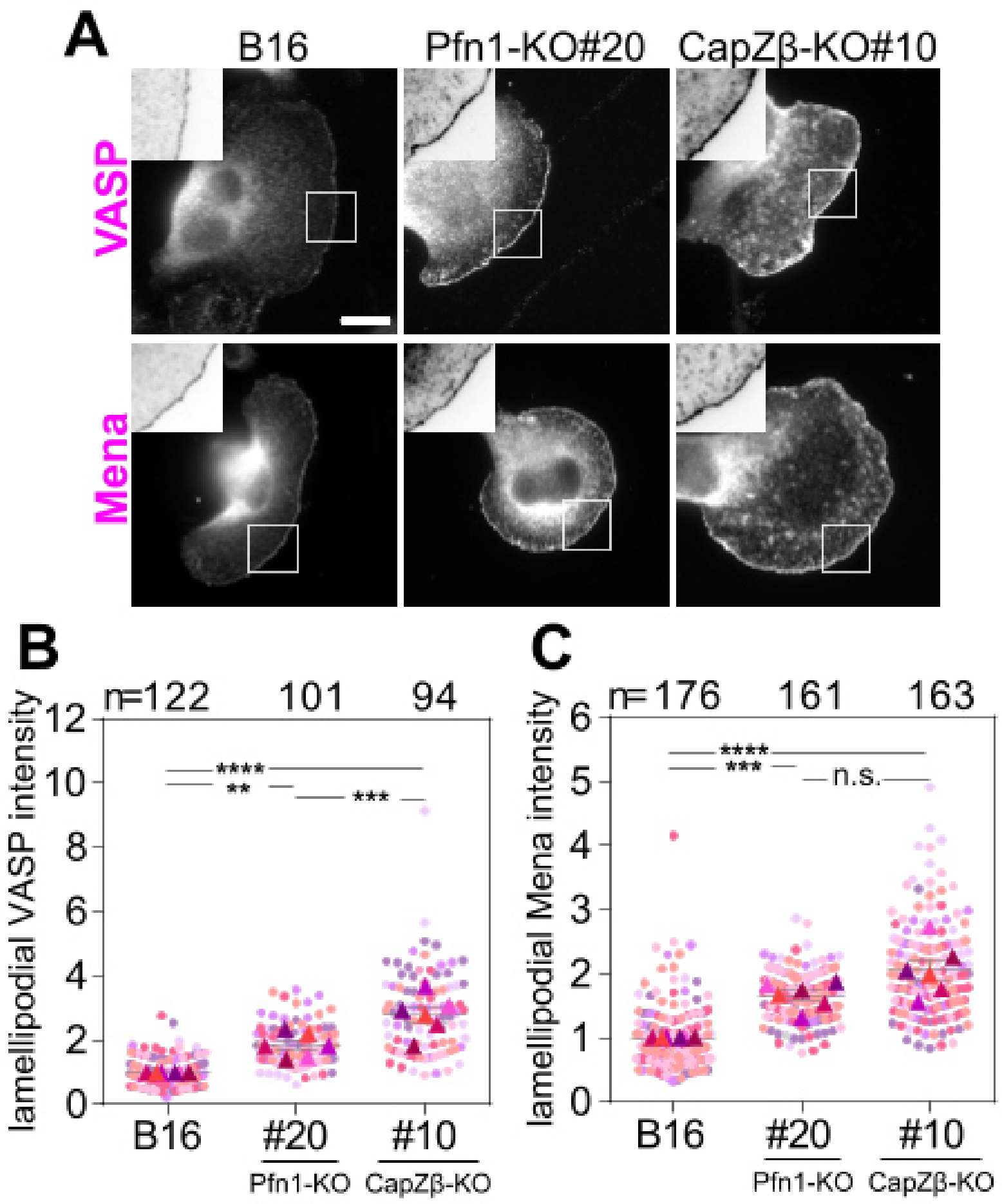

Supplementary Fig. 9. Pfn1-KO induced-upregulation of lamellipodial Ena/VASP family members is phenocopied in CP-KO. (**A** - **C**) Comparison and quantitation of Ena/VASP protein accumulation at the lamellipodium tip in B16-F1 wildtype (WT) *versus* Pfn1-KO (clone #20) and a B16-F1 cell line disrupted for heterodimeric CP (CapZβ-KO#10)[6]. Representative stainings of cells of respective genotype are shown in A, as indicated. White rectangles in subpanels in A correspond to insets magnified and inverted at the respective top left of each panel, illustrating the increase in accumulation at the lamellipodium edge in Pfn1- and even more so CP-KO cells. Scale bar, 10 µm valid for all subpanels. For quantitations in B and C, intensities measured for VASP and Mena were normalized to the arithmetic mean of the WT population in each individual experiment (colored rectangles), n=measured cells from at least six independent experiments in this case. The arithmetic means of replicates of all datasets in B and C were statistically compared with each other using ANOVA with Tukey's posthoc test; n.s. not significant; **p≤0.01; ***p≤0.005; ****p≤0.001.

Supplementary Fig. 10

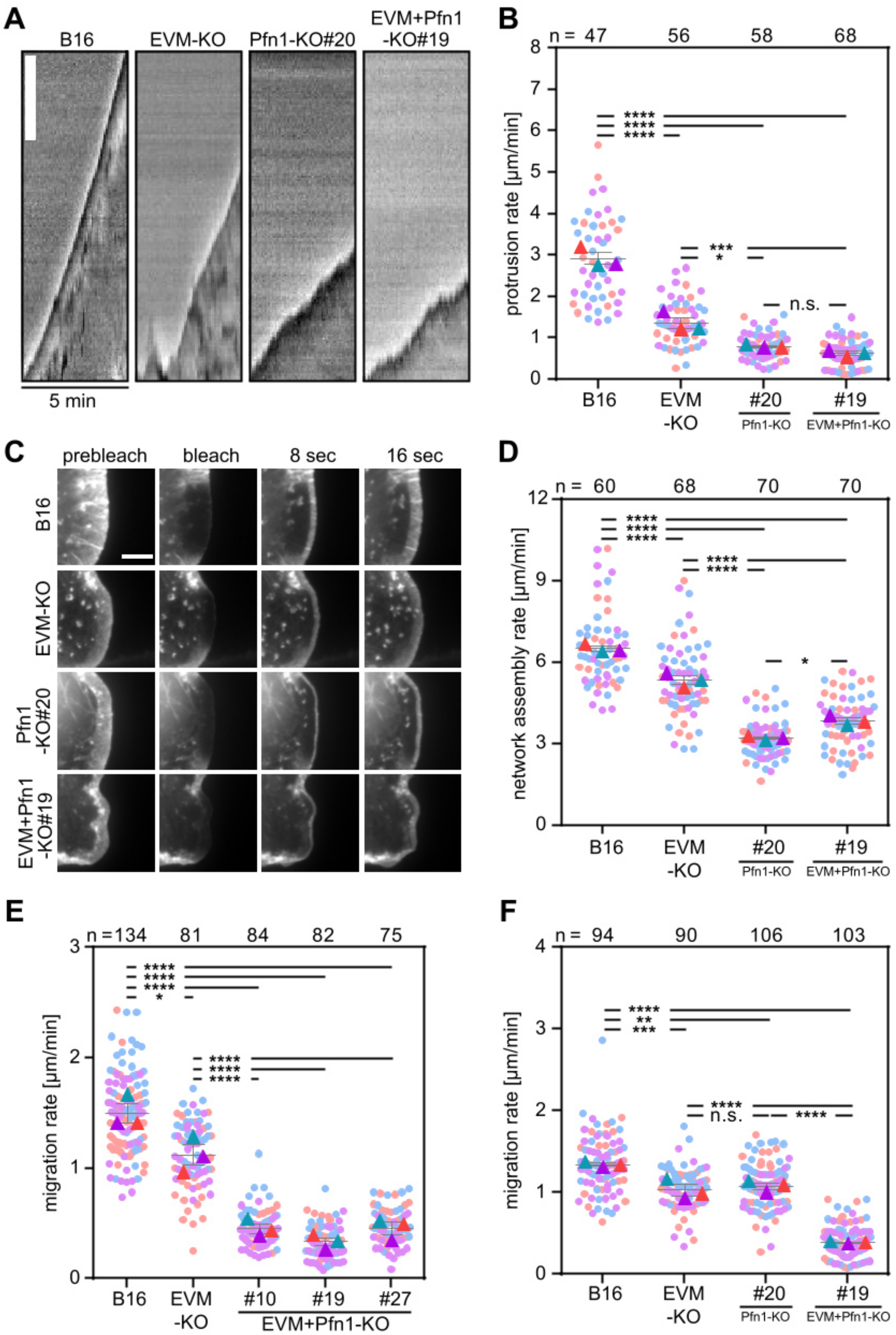

Supplementary Fig. 10. Combined Ena/VASP and profilin removal differentially affects lamellipodial protrusion, actin network assembly and overall random cell migration. (**A**) Side-by-side kymography of representative lamellipodial edges of EVM-KO (clone #23.7.66), Pfn1-KO (clone #20) and EVM+Pfn1 quadruple-KO (clone #19) from data acquired by time-lapse phase contrast microscopy; time is as provided and white scale bar corresponds to 3 µm. (**B**) Quantitation of lamellipodial protrusion rates determined from kymographs of individual lamellipodia (shown in A) for the different cell types. The protrusion rates determined for each cell line in three independent experiments are shown as superplots, displaying measurements from individual cells and their arithmetic means as color-coded dots and triangles, respectively. Grey lines and error bars display means and standard errors of the means (SEMs), n = numbers of measured cells. For statistics, the arithmetic means of replicates of all datasets were compared with each other in all combinations using ANOVA with Tukey's posthoc test; n.s. not significant; *p≤0.05; ***p≤0.005; ****p≤0.001. (**C**) Fluorescence recovery after photobleaching (FRAP) analysis in lamellipodia of cells with indicated genotype transiently expressing EGFP-tagged actin. As before, fluorescence recovery from the front following the bleach is taken as direct readout of lamellipodium network assembly rate. Scale bar, 5 µm. (**D**) Quantitation of network assembly rate assessed in cell lines as representatively shown in C. Data are displayed as superplots analogous to what's reported for protrusion rates in B. (**E**) Quantitation of random migration rates of B16-F1 wildtype (B16) *versus* EVM-KO (clone #23.7.66) *versus* the three selected EVM+Pfn1 quadruple-KO cell lines. Migration rates were extracted from tracks (see below) over time periods of 10 hours. Data are again displayed as superplot data; three independent experiments (distinct colors). For statistics in D and E, see BB; *p≤0.05; ****p≤0.001. (**F**) Side-by-side comparison of the impact on cell migration of EVM- *versus* Pfn1-removal or both combined using individual cell lines, as indicated. Data are displayed as described for E. For representative movies serving as raw data for the quantitation, see Supplementary Movie 8. Statistics: comparison of arithmetic means of replicates using ANOVA with Tukey's posthoc test; n.s. not significant; **p≤0.01; ***p≤0.005; ****p≤0.001.

Supplementary Fig. 11

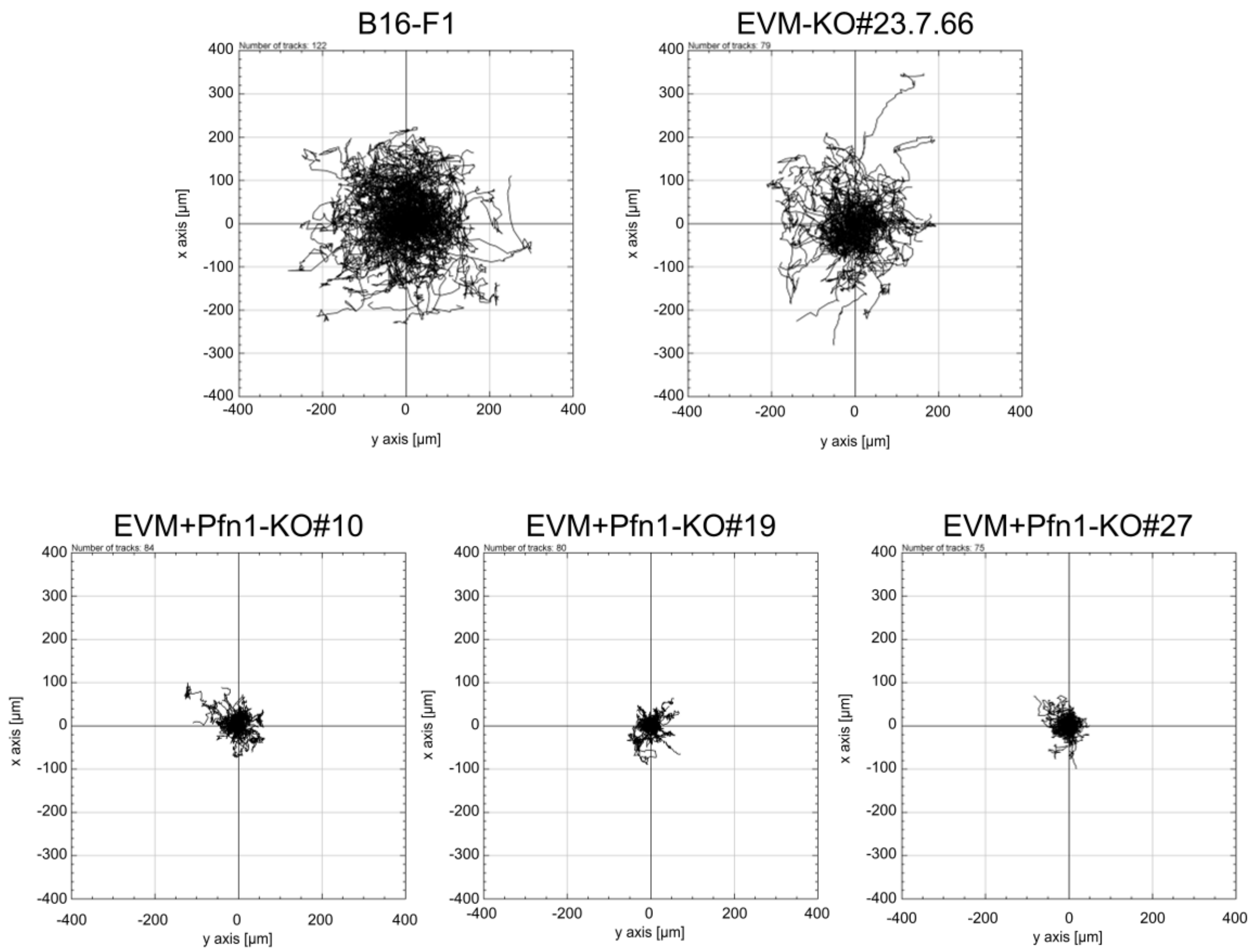

Supplementary Fig. 11. Profilin removal virtually abolishes cell migration in the absence of Ena/VASP family members. Random migration performance of B16-F1 cells lacking EVM alone *versus* both EVM and Pfn1, as indicated. Trajectory plots as obtained from random migration movies of distinct cell lines migrating on laminin. Black trajectories are individually tracked cells, the starting points of which were positioned in the center of the coordinate system. Cells were tracked for a maximum of 10 hours. Note the comparably moderate size reduction of trajectory plots as compared to wildtype in the cell line lacking EVM, consistent with reduced migration rates independently measured in Figs. S10E and S10F, but the dramatic compression of trajectory plot sizes in all clones (#10, #19, #27) lacking both EVM and Pfn1.

Supplementary Fig. 12

The mathematical model

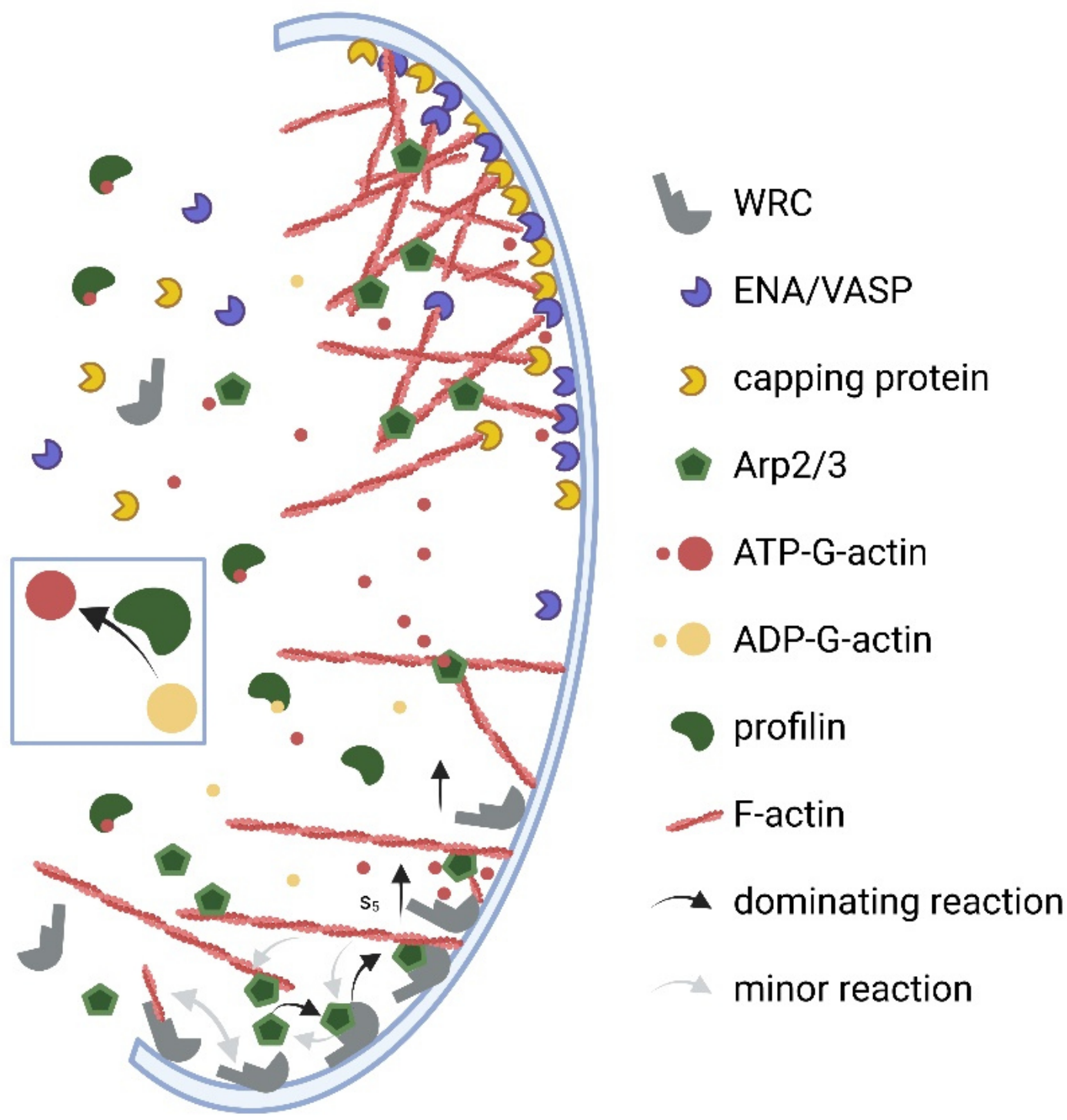

Figure M1. Reactions defining the model. WRC translocates from the cytosol to the membrane. It binds Arp2/3 complex and the barbed ends of actin filaments. The WRC-Arp2/3 complex binds to the side of filaments. The WRC-Arp2/3-filament complex forms a branch, the WRC-Arp2/3 bond dissociates without branch formation or the filament dissociates. VASP and capping protein (CP) bind to the membrane and compete with each other for binding sites on the membrane. Membrane-bound CP and membrane-bound VASP also compete with each other for filament barbed ends. CP bound to barbed ends quickly disappears from the tip membrane due to transport with retrograde flow. The VASP-filament complex can dissociate, stay, elongate the filament or be transported away with retrograde flow if elongation is too slow or stopped by some unspecified reason.

The model comprises the dynamic interactions of F-actin filaments and/or their barbed ends with WAVE regulatory complex (WRC), Arp2/3 complex, capping protein (CP), Ena/VASP family proteins (VASP) and the protrusion tip membrane. Fig. M1 illustrates the reactions defining the model. If we include a binding reaction, we also include the corresponding dissociation unless retrograde flow transports the bound molecule away. This applies to CP bound to filament tips and Arp2/3 bound to filament sides.

Suppl. Table 1. Variables of the model	
variable	description
wm	WRC
wa	WRC-Arp2/3 complex
wf	WRC-filament complex
waf	WRC-Arp2/3-filament complex
f	free barbed end
vf	VASP bound to barbed ends
vm	VASP bound to tip membrane
cm	CP bound to tip membrane

Model variables are listed in Supplementary Table 1. The variable wm is bare WRC bound to the tip membrane. WRC arrives at the tip membrane by diffusion and then binds to the membrane. This is described by the first term of the wm-dynamics below with the rate constant s_{10}. W is the concentration of wm on the tip membrane that is in equilibrium with the bulk concentration of WRC. The turnover kinetics of WRC measured by FRAP[1, 2] supports our choice for this binding term.

The two following terms describe binding (s_1) of cytosolic Arp2/3 and spontaneous dissociation (s_2) of Arp2/3 complex. A_{bulk} denotes the (cytosolic) pool of unbound Arp2/3 complexes. Terms 4 and 5 describe the free filament end binding (s_3) to WRC and its dissociation (s_4):

$$\frac{d[wm]}{dt}=s_{10}\left(W-[wm]\right)-s_1A_{bulk}[wm]+s_2[wa]+s_4[wf]-s_3[wm][f]+(1-A_r)s_{54}R[waf]. \qquad (M1)$$

The last term (s_{54}) arises from breaking bonds in the complex WRC-Arp2/3 bound to a filament (waf). Bonds within this complex are under force due to network assembly and retrograde flow. The faster the network assembly rate R, the sooner the tension on these bonds will rise. Therefore, we set the rate, with which bonds within this complex break, proportional to R. Simulations including a more detailed force balance and an exponential dependency on R provided results very similar to those with

a linear R-dependence. On this basis, we chose the simpler linear rate expression. If a bond of the complex WRC-Arp2/3 bound to a filament breaks, the fraction A_r breaks between filament and WRC-Arp2/3 and 1-A_r between WRC and Arp2/3. If none of the bonds break, a new filament is nucleated and is initially bound to WRC (variable wf). This happens with the rate proportional to s_5 in the wf-dynamics

$$\frac{d[wf]}{dt}=s_3[wm][f]-s_4[wf]+s_5[waf]-s_{40}[wf]-s_{18}R[wf]. \quad \text{(M2)}$$

WRC-bound filaments also arise from free ends binding to wm. Here and in all following equations, s_{40} is the rate constant of loss of filaments e.g. due to severing. The term with the rate $s_{18}R$ is proportional to the network assembly rate R and describes the loss of WRC-bound filaments from the tip membrane due to transport with retrograde flow. The model fit introduced below showed that both severing and transport contribute little to the dynamics of filament tips at the protrusion tip.

The dynamics of Arp2/3 complex-bound WRC, variable wa, comprises binding of free Arp2/3 (A_{bulk}) and its dissociation. The WRC-Arp2/3 complex may bind (s_{51}) to the sides of filaments with free ends f, WRC-bound filaments wf, VASP bound filaments vf or filaments from other populations f_e, like e.g. formin-bound ones (3. term). The dissociation of Arp2/3 complex-filament bonds enters by the last term:

$$\frac{d[wa]}{dt}=s_1A_{bulk}[wm]-s_2[wa]-s_{51}[wa]\left([f_e]+[wf]+[f]+[vf]\right)+A_rs_{54}R[waf]. \quad \text{(M3)}$$

The binding of the WRC-Arp2/3 complex (wa) to a filament entails either formation of a branch (s_5) or breakage of the WRC-Arp2/3-filament complex ($s_{54}R$):

$$\frac{d[waf]}{dt}=s_{51}[wa]\left([f_e]+[wf]+[f]+[vf]\right)-s_5[waf]-s_{40}[waf]-s_{54}R[waf]. \quad \text{(M4)}$$

The binding term and the break-term do not show up in the dynamics for filaments, since Arp2/3 complex binds to the sides of filaments belonging to the populations [f], [wf], [vf] or [f_e], without changing filament numbers in these populations. However, the term branch generation creates a new filament and thus shows up in the [wf]-dynamics.

The first and second terms in the dynamics of free barbed ends [f] describe dissociation from WRC and binding of free barbed ends to WRC. The next two terms represent the same for binding of VASP bound to the tip membrane (s_{70}, s_{71}). The term with the rate s_{82} describes capping by CP bound to the tip membrane cm. Dissociation of CP from filament tips does not contribute to the [f] dynamics, as we assume that capped barbed ends are immediately transported away with retrograde flow. The term with the rate $s_{38}R$ describes the loss of free filament ends from the tip membrane due to advection with retrograde flow:

$$\frac{d[f]}{dt}=s_4[wf]-s_3[wm][f]+s_{71}[vf]-s_{70}[vm][f]-s_{82}[cm][f]-s_{38}R[f]-s_{40}[f]. \quad \text{(M5)}$$

The experimental results comprised the interesting observation of an increase in leading edge VASP accumulation upon Pfn1 knockout, in spite of decreasing F-actin density. This implies the existence of VASP binding sites at the membrane (m) in addition to barbed ends. We denote those, occupied VASP-binding sites vm. A competition between VASP and CP at the leading edge of lamellipodia was previously proposed in the context of competition for barbed end binding. This competition is experimentally supported by the observation of increased CP accumulation in the absence of Ena/VASP[3], and the vice versa experiment (this study, Supplementary Fig. 9A-C). This means that VASP intensity at the lamellipodium tip goes down when CP goes up and vice versa. The existence of VASP binding sites at the tip membrane renders such a competition impossible unless VASP and CP compete not only for barbed ends, but also for these tip membrane binding sites m. Denoting the binding sites occupied with CP cm, the following dynamics realize such a competition:

$$\frac{d[vm]}{dt}=s_{60}V_{bulk}\left(m-[vm]-[cm]\right)-s_{61}[vm]-s_{70}[vm][f] \tag{M6}$$

$$\frac{d[cm]}{dt}=s_9C_{bulk}\left(m-[vm]-[cm]\right)-s_{91}[cm]-s_{82}[cm][f]. \tag{M7}$$

V_{bulk} and C_{bulk} denote the cytoplasmic concentrations of VASP and CP, respectively. We have chosen here the simplest possible dynamics recruiting VASP and CP to the membrane, since we lack detailed information on the process. Those dynamics are able to reproduce the experimental results in good approximation. However, the recruitment might be more complicated and might comprise additional steps. The term with s_{61} describes VASP dissociation back into the cytosol. The experimental data show an increase of VASP at the lamellipodium edge concomitant with a decrease of F-actin density (see above). We thus suggest that VASP binding to barbed ends causes a flux of VASP out of the vm-population. We assume on this basis that VASP dissociating from barbed ends does not return to the membrane binding sites, i.e. to vm, immediately, but instead diffuses back into the cytosol. The last term describes binding of membrane-bound VASP to barbed ends.

The dynamics of barbed ends bound to membrane-bound VASP also includes a transport term. The other terms in the vf-dynamics are free end binding and dissociation.

$$\frac{d[vf]}{dt}= s_{70}[vm][f]-s_{71}[vf]-s_{32}R[vf]-s_{40}[vf]. \tag{M8}$$

All variables are defined as numbers per cross section in case of filaments and concentrations on the tip membrane for the other variables. Experimental results are provided as concentration ratios relative to wildtype. We lack information on absolute concentrations. Consequently, we scale all dynamic model variables with W. This scaling does not affect concentration ratios. We are exclusively considering stationary states. Therefore, we cannot make any statements on absolute rate values and consequently scale time with s_{10}. Thus, we obtain dimensionless parameter values by the scaling:

$$\frac{d[wm]}{d\tau}=\left(1.0-[wm]\right)-k_1[wm]+k_2[wa]+k_4[wf]-K_3[wm][f]+\left(1-A_r\right)k_{54}R[waf]$$

$$\frac{d[wf]}{d\tau}=K_3[wm][f]+k_5[waf]-k_4[wf]-k_{40}[wf]-k_{18}R[wf]$$

$$\frac{d[waf]}{d\tau}=K_{51}[wa]\left([F_e]+[wf]+[f]+[vf]\right)-k_5[waf]-k_{40}[waf]-k_{54}R[waf]$$

$$\frac{d[wa]}{d\tau}=k_1A_{bulk}[wm]-k_2[wa]-K_{51}[wa]\left([F_e]+[wf]+[f]+[vf]\right)+A_rk_{54}R[waf]$$

$$\frac{d[f]}{d\tau}=k_4[wf]-K_3[wm][f]+k_{71}[vf]-K_{70}[vm][f]-K_{82}[cm][f]-k_{38}R[f]-k_{40}[f]$$

$$\frac{d[vf]}{d\tau}= k_{70}[vm][f]-k_{71}[vm]-k_{32}R[vm]-k_{40}[vm]$$

$$\frac{d[vm]}{d\tau}=k_{60}\left(M-[vm]-[cm]\right)-k_{61}[vm]-K_{70}[vm][f]$$

$$\frac{d[cm]}{d\tau}=k_9\left(M-[vm]-[cm]\right)-k_{91}[cm]-K_{82}[cm][f]$$

$$\tau=s_{10}t,\ \ k_i=\frac{s_i}{s_{10}},\text{ but } k_1=\frac{s_1A_{bulk}}{s_{10}},\ k_{60}=\frac{s_{60}V_{bulk}}{s_{10}},\ k_9=\frac{s_9C_{bulk}}{s_{10}} \tag{M9}$$

$$K_3=k_3W,\quad K_{51}=k_{15}W,\quad K_{70}=k_{70}W,\quad K_{82}=k_{82}W,\quad M=\frac{m}{W},\quad F_e=\frac{f_e}{W}.$$

Here, the names for the scaled dynamic variables have remained like the unscaled ones and the parameter names have been changed only for those affected by the scaling. The fit to the experimental results has been done with the scaled model, which is possible since all results are concentration ratios.

**Supplementary Table 2. Parameters of the model**		
parameter	description	scaled parameter
s_1	binding rate of Arp2/3 to WRC	$k_1=s_1 A_{bulk}/s_{10}$
A_{bulk}	Arp2/3 cytosolic concentration	
s_{10}	equilibration rate of total WRC on the membrane	
Rs_{18}	rate of WRC-filament complex loss due to retrograde flow	$k_{18}=s_{18}/s_{10}$
s_2	dissociation rate of Arp2/3 complex from WRC	$k_2=s_2/s_{10}$
s_3	binding rate of filaments to WRC	$K_3=Ws_3/s_{10}$
Rs_{32}	rate of VASP-bound barbed end loss due to retrograde flow	
Rs_{38}	rate of barbed end loss due to retrograde flow	
R	network assembly rate	
s_4	dissociation rate of filaments from WRC	$k_4=s_4/s_{10}$
s_{40}	rate of filament loss due to severing, bundling, and unspecified processes	$k_{40}=s_{40}/s_{10}$
s_5	rate of branch formation	$k_5=s_5/s_{10}$
s_{51}	binding rate of Arp2/3-WRC complexes to filaments	$K_{51}=Ws_{51}/s_{10}$
Rs_{54}	dissociation rate of WRC-Arp2/3-filament complexes without branch formation	$k_{54}=s_{54}/s_{10}$
$(1-A_r)\ Rs_{54}$	dissociation rate of Arp2/3-filament complexes and WRC without branch formation	
$A_r\ Rs_{54}$	dissociation rate of filaments and Arp2/3-WRC complexes without branch formation	
A_r	fraction of dissociation events of WRC-Arp2/3-filament complexes without branch formation occurring at the Arp2/3-filament bond	
s_{60}	rate of VASP binding to tip membrane	$k_{60}=s_{60} V_{bulk}/s_{10}$
s_{61}	rate of VASP dissociation from tip membrane	$k_{61}=s_{61}/s_{10}$
s_{70}	rate of membrane-bound VASP binding to barbed ends	$K_{70}=Ws_{70}/s_{10}$
s_{71}	rate of VASP dissociation from barbed ends	$k_{71}=s_{71}/s_{10}$
s_{82}	rate of membrane-bound CP binding to barbed ends	$K_{82}=Ws_{82}/s_{10}$
s_9	rate of CP binding to tip membrane	$k_9=s_9 C_{bulk}/s_{10}$
s_{91}	rate of CP dissociation from tip membrane	$k_{91}=s_{91}/s_{10}$
f_e	filaments bound to formins or other membrane proteins	$F_e=f_e/W$
V_{bulk}	cytosolic concentration VASP	
C_{bulk}	cytosolic concentration of CP	
m	concentration of binding sites for VASP and CP on the tip membrane	M=m/W
W	equilibrium concentration of WRC on the tip membrane	

Fitting experimental results

The experimental results constitute a set of measured ratios of densities of lamellipodial factors in various conditions close to or at protrusion tip membranes. Molecules which are being transported away with retrograde flow from the tip membrane contribute to the fluorescence intensity as long as they are close to the tip membrane. Therefore, we include them in the ratio calculation. The volume density of these molecules in the F-actin network is given by the rate with which they leave the tip membrane divided by the network assembly rate R. Since the lamellipodium is not of cuboid shape the rate of molecules leaving the membrane per cross sectional area comprises a geometrical factor g,

which we could determine exactly only with a spatially detailed model and detailed knowledge on membrane geometry. For example, the volume density of CP is gs_{82}[cm][f]/R. Multiplying with the height h_L of the lamellipodium and adopting our scaling leads to the area density $h_L s_{10} g W K_{82}$[cm][f]/R. The height of the lamellipodium is not constant, in the range of 0.1 µm and tapers towards the protrusion tip[4, 5]. Due to the lack of knowledge on the exact values of h_L, s_{10} and g, we approximate $h_L s_{10} g$ by 1 µm min^{-1}, which implies a value of s_{10} of order 20 min^{-1}, if g is of order 1. We define the scaled velocity $R_s=R/h_L s_{10} g$.

Filament tips transported with retrograde flow contribute on average one half of their density to the F-actin density, since the filament does not contribute between barbed end and protrusion tip membrane to F-actin density. Thus, we obtain for total amounts of the scaled concentrations

$$\begin{aligned}
&\text{total WRC} && =[wm]+[wf]\left(1+\frac{k_{18}R}{R_s}\right)+[wa]+[waf] \\
&\text{total Arp2/3} && =[wa]+[waf]\left(1+\frac{k_5}{R_s}\right) \\
&\text{total filament density} && =F_e+[f]\left(1+\frac{k_{38}R}{2R_s}\right)+[wf]\left(1+\frac{k_{18}R}{2R_s}\right)+[vf]\left(1+\frac{k_{32}R}{2R_s}\right)+\frac{k_{82}[cm][f]}{2R_s} \qquad \text{(M10)} \\
&\text{total VASP} && =[vf]\left(1+\frac{k_{32}R}{R_s}\right)+[vm] \\
&\text{total capping protein} && =[cm]+\frac{K_{82}[cm][f]}{R_s}.
\end{aligned}$$

We model Pfn1-KO by reducing the branching rate k_5 by the factor f_{pfn}. The branching rate is proportional to the square of the concentration of polymerizable G actin according to Carlsson and colleagues[6, 7]. Hence, the factor by which polymerizable G-actin is reduced in Pfn1-KO is $\sqrt{f_{pfn}}$. We model Ena/VASP- and CP-KO by setting the values of V_{bulk} and C_{bulk}, respectively, to 1% of their control values. Consequently, k_{60} and k_9 have 1% of their control values in those knockouts. Setting them to 0 would have rendered the Jacobi matrix of the system singular, which we chose to avoid since we also used implicit integration methods and Newton Raphson methods to determine stationary states (see section Numerical methods). The value of R has been determined in experiments (see Supplementary Table 4).

Results

Supplementary Table 3 displays 13 values to which we can fit the mathematical model. Additionally, we know that VASP increased 2-3fold upon CP KO, such that 14 values need to be fit by the model outcome. The network assembly rate is a parameter in our modelling (Supplementary Table 4).

Supplementary Table 3. Experimental results constituting fit criteria for the model

	EVM-KO ratio with control	Pfn1-KO ratio with control	EVM+Pfn1-KO ratio with control
F-actin	0.506	0.495	0.303
Arp2/3	1.29	0.491	0.397
WRC	0.934	0.677	0.500
CP	1.71	0.602	1.80
VASP/Mena	0.0	3.20/1.75	0.0

Supplementary Table 4. Experimental results for the network assembly rate (parameter R)

	control	EVM-KO	Pfn1-KO	EVM+Pfn1-KO
network assembly rate	6.48 µm min^{-1}	5.3 µm min^{-1}	3.18 µm min^{-1}	3.81 µm min^{-1}

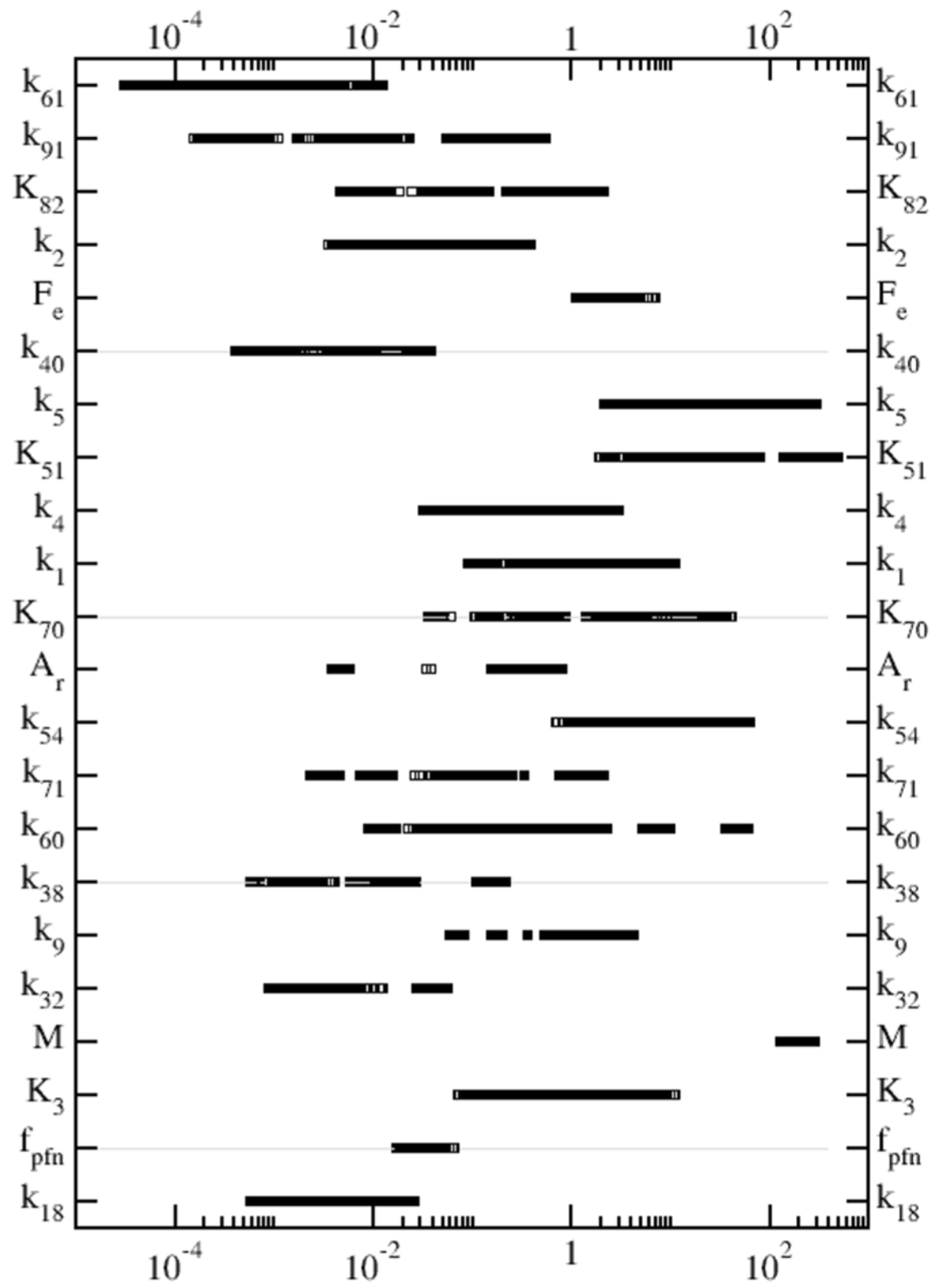

Figure M2. Values of the scaled parameters defined in Eqs. 1,2 resulting from the fits in Fig. 7E. The model output ranges represented comprise 2218 parameter value sets each meeting the criteria. They were found in a sample of 5 10^9 parameter value sets.

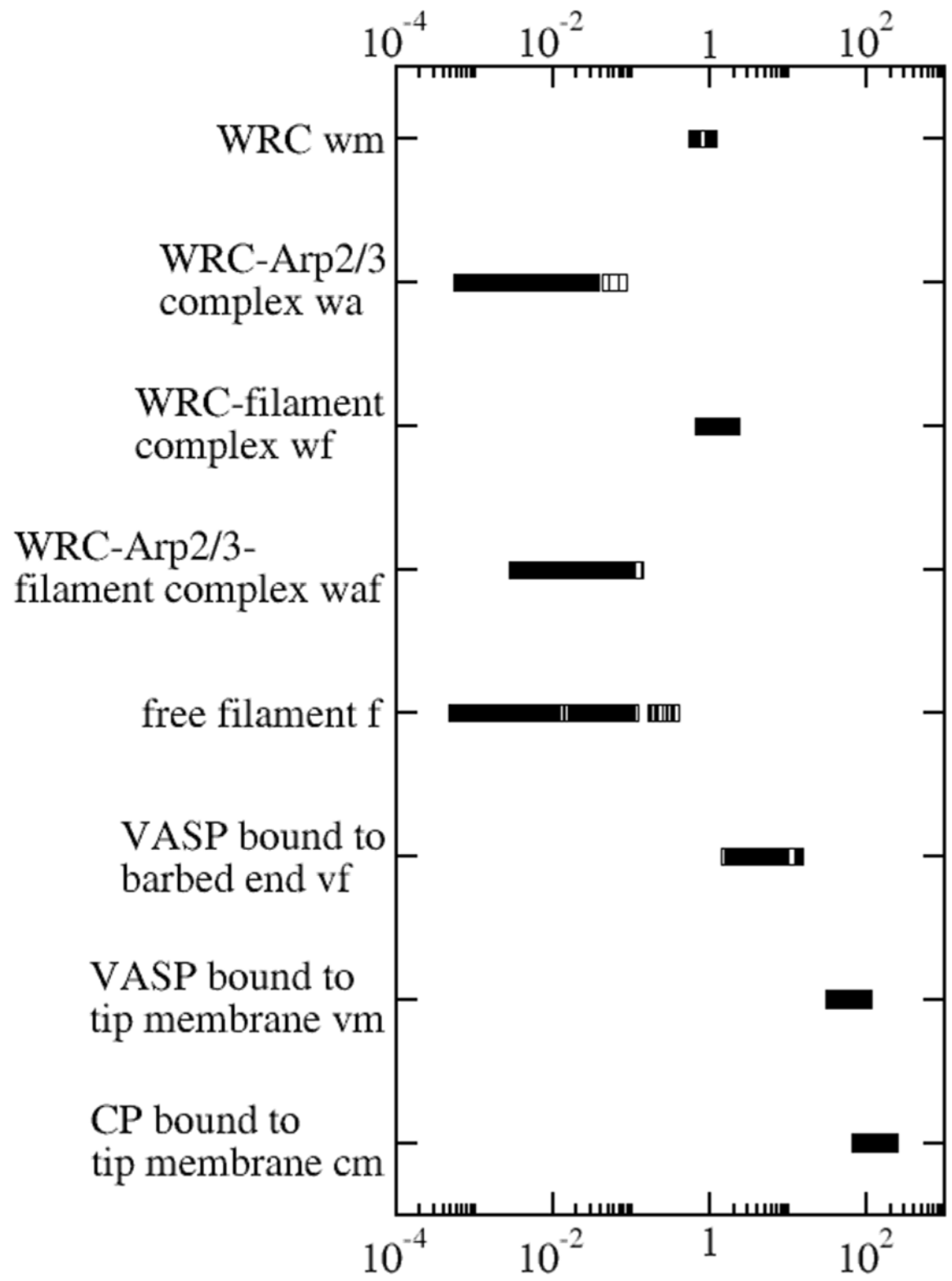

Figure M3. The values of the scaled control variables resulting from the model fits in Fig. 7E. The model output range represented comprises 2218 parameter value sets each meeting the criteria. They were found in a sample of 5 10^9 parameter value sets. All variables are defined on the membrane or cross section density in case of filaments. CP bound to barbed end is not among the model variables since capped barbed ends leave the membrane immediately upon capping.

We defined a fit criterion as met if the corresponding model variable output was within ±20% of each value listed in Supplementary Table 3. VASP increased 3.2fold and Mena 1.75 upon Pfn1-KO. Within this range, we defined the criterion (called VASP Pfn1-KO in Fig. 7E) met if model output was larger than 2.0. We find values up to about 2.2 as simulation output (but see next section also). We found parameter value sets producing outputs within our 20%-range for all the other criteria.

The parameter values and variable values resulting from the fits are shown in Figs. M2 and M3. We find fits to the fit criteria in Fig. 7E for rather large ranges of parameter values. Hence, the mechanism formulated in the model does not depend on very specific assumptions on parameter values (see parameter value ranges in Fig. M2), but instead is rather robust.

The largest fraction of filaments is VASP-bound, followed by WRC-bound. Filaments with WRC-bound Arp2/3 bound to their sides are in the range below 1% of total filament numbers. Filaments with free barbed ends in the range below 2%.

The fastest scaled rate in Fig. M2 is K_{51}, the binding of WRC-bound Arp2/3 to filament. The parameter f_{pfn} describes the modelling of Pfn1-KO. We model it as a reduction of the branching rate s_5 by this factor. Its value is in the range of 0.017-0.083. The branching rate is proportional to the square of the concentration of polymerizable G actin.[6, 7] Hence, the factor by which polymerizable G-actin is reduced by Pfn1 KO is $\sqrt{f_{pfn}}$ corresponding to a reduction of polymerizable G-actin to about 13.0%-28.7% of its control value in Pfn1-KO. The expression of actin in Pfn1-KO cells was measured to be reduced to

between 35% and 42%, dependent on the Pfn1-KO clone (main text Fig. 1G). Additionally, the fraction of ATP-G-actin of this reduced amount of actin is likely reduced compared to control, since profilin catalyses the exchange of ADP for ATP on monomeric actin. We conclude that our reduction of polymerizable G-actin in the model as consequence of Pfn1-KO might be realistic.

The slowest rate is the spontaneous dissociation rate k_{61} of VASP from the membrane binding site m. The spontaneous dissociation of CP from the membrane binding site is also slow. Binding of VASP and CP with the rates k_{60} and k_9 resp. to m is also slow. The binding of VASP and CP to barbed ends has faster rates (K_{70} and K_{82}) than binding of VASP and CP to m. Therefore, most of the membrane-bound VASP and CP molecules will bind barbed ends, at least with control values of barbed end density.

Other slow processes, i.e. processes not dominating the dynamics, are the losses due to transport by retrograde flow of WRC bound to filament (k_{18}), VASP bound to filament (k_{32}), free barbed ends (k_{38}) and filament loss due to severing (k_{40}).

The parameter A_r describes the fraction of bonds in the WRC-Arp2/3-filament complex breaking between WRC and Arp2/3 upon stress. In principle, it can assume values between 0 (no dissociation of the filament from the WRC-Arp2/3-filament complex) and 1 (no dissociation of the WRC from the WRC-Arp2/3-filament complex). Fig. M2 shows that we find fits for the whole range of values. Hence, the mechanism formulated in the model does not depend on assumptions on this fraction.

Lower margin for fit criterion on ratio of CP upon Pfn1-KO to control

If we allow for smaller values of the ratio of CP upon Pfn1-KO to control than our 20% criterion, we find larger values of the ratio of VASP upon Pfn1-KO to control, i.e. up to 2.62 (Fig. M4). This confirms the anti-correlation between VASP and CP. Parameter values and variable values resulting from the fit criteria in Fig. M4 are shown in Figs. M5 and M6.

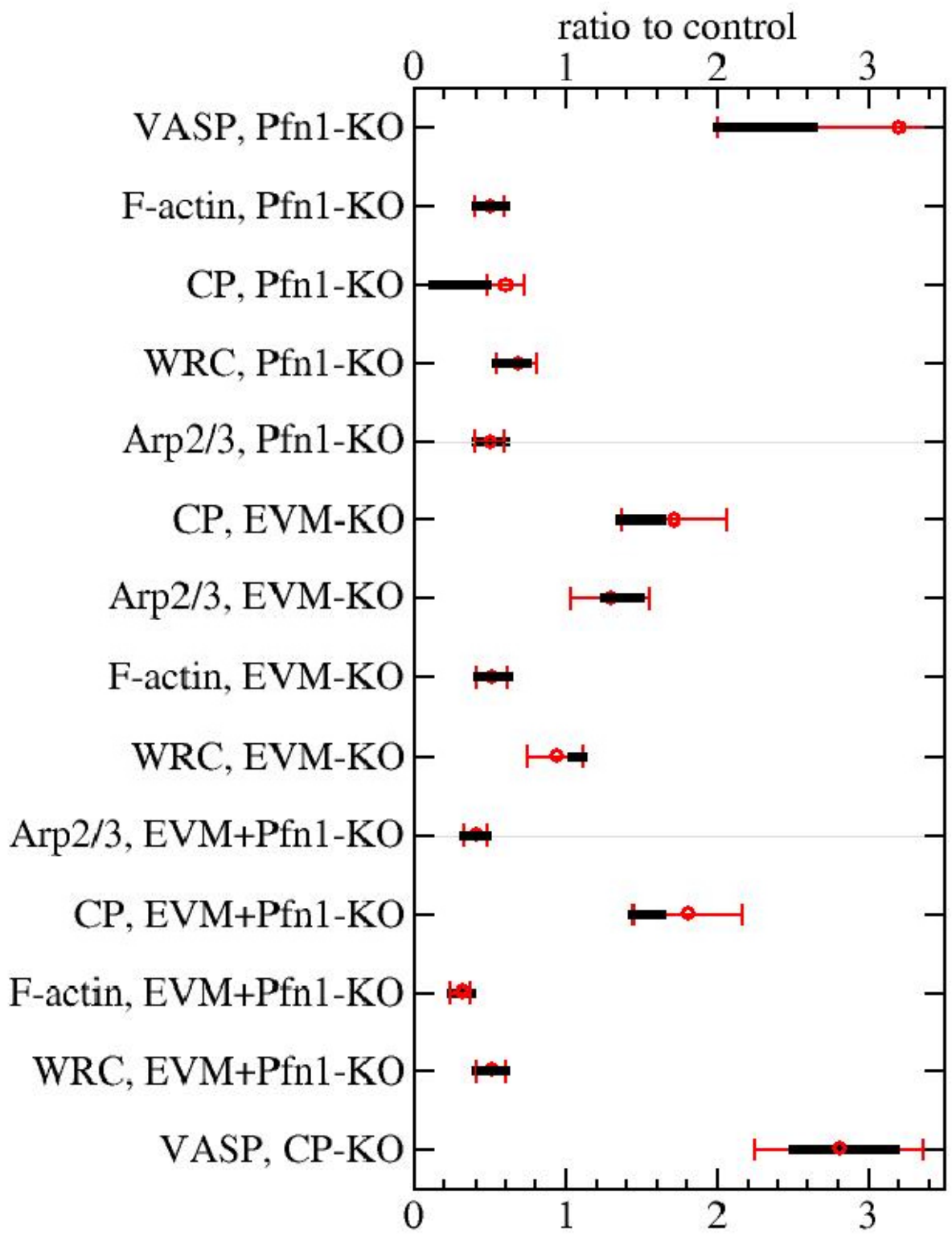

Figure M4. Fit results for the 14 fit criteria. We considered a criterion as met if the model output was in the range indicated by the red lines, except for the criterion 'CP, Pfn1 KO'. We show here results with this criterion being below the acceptance range. The model output range represented by black lines comprises 169.994 parameter value sets each meeting the criteria. They were found in a sample of 5 10^9 parameter value sets.

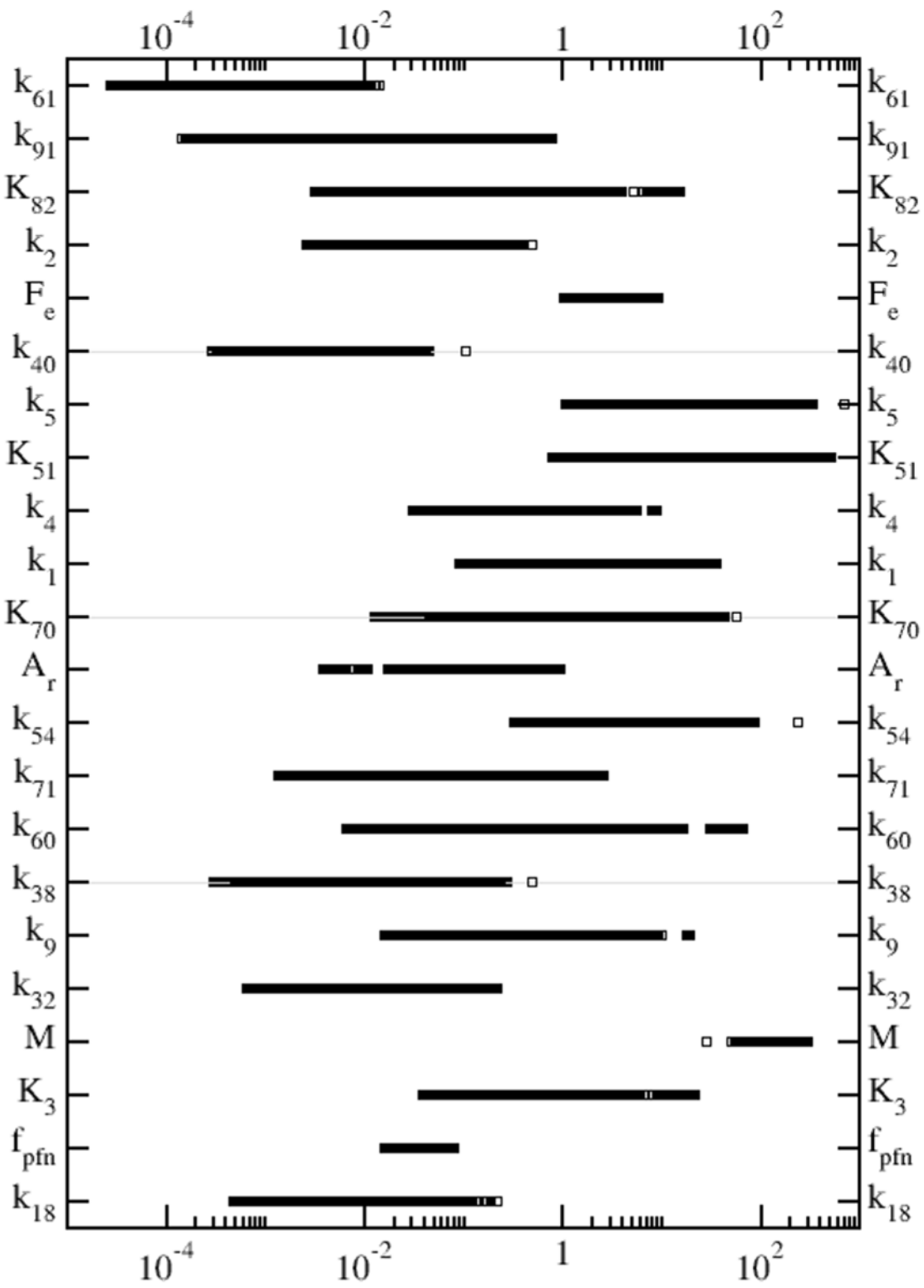

Figure M5. Values of the scaled parameters defined in Eqs. 1,2 resulting from the fits in Fig. M4.

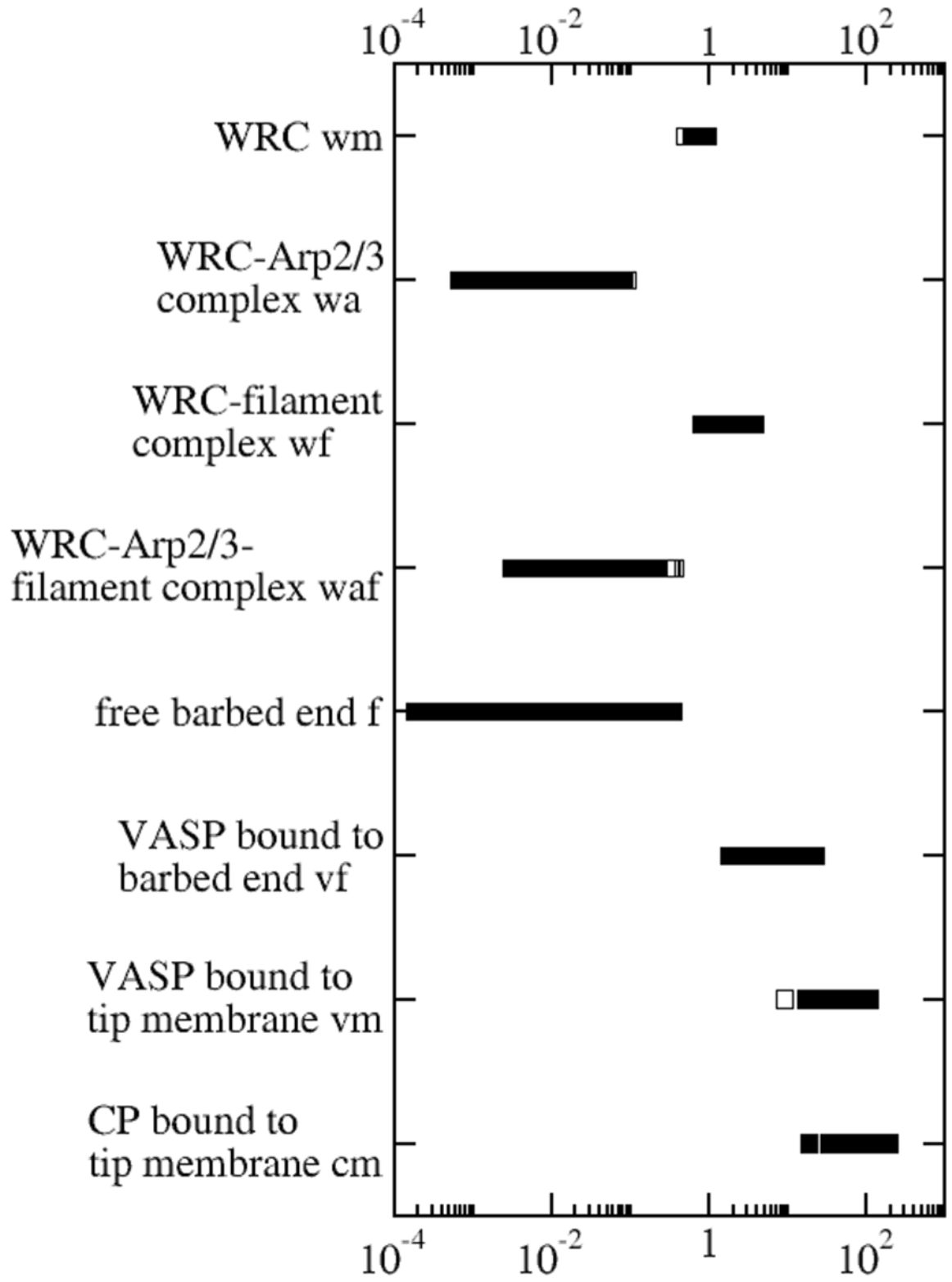

Figure M6. The values of the scaled control variables resulting from the model fits in Fig. M4.

Numerical methods

Most stationary states were found with a Newton-Raphson iteration[8]. The stability of selected stationary points found with this method was checked by integration of the ODEs. If Newton-Raphson iterations did not converge, we alternated integration of the ODEs and Newton-Raphson till convergence was reached. Computations have been carried out on the high performance compute cluster of the Max Delbrück Center. The calculations took about 9 months using on average 80 cores.

References

1. Lai, F. P., *et al.* Arp2/3 complex interactions and actin network turnover in lamellipodia. *EMBO J* **27**, 982–992 https://doi.org/emboj200834 [pii]10.1038/emboj.2008.34 (2008).
2. Schaks, M., *et al.* Distinct Interaction Sites of Rac GTPase with WAVE Regulatory Complex Have Non-redundant Functions in Vivo. *Curr Biol* **28**, 3674–3684 e3676 https://doi.org/10.1016/j.cub.2018.10.002 (2018).
3. Damiano-Guercio, J., *et al.* Loss of Ena/VASP interferes with lamellipodium architecture, motility and integrin-dependent adhesion. *Elife* **9**, https://doi.org/10.7554/eLife.55351 (2020).
4. Koestler, S. A., *et al.* F- and G-Actin Concentrations in Lamellipodia of Moving Cells. *PLoS one* **4**, e4810 (2009).
5. Urban, E., Jacob, S., Nemethova, M., Resch, G. P. & Small, J. V. Electron tomography reveals unbranched networks of actin filaments in lamellipodia. *Nat Cell Biol* **12**, 429–435 (2010).
6. Carlsson, A. E. The effect of branching on the critical concentration and average filament length of actin. *Biophys J* **89**, 130–140 https://doi.org/10.1529/biophysj.105.061598 (2005).
7. Carlsson, A. E., Wear, M. A. & Cooper, J. A. End versus side branching by Arp2/3 complex. *Biophys J* **86**, 1074–1081 https://doi.org/10.1016/S0006-3495(04)74182-X (2004).
8. Press, W. H., Teukolsky, S. A., Vetterling, W. T. & Flannery, B. P. *Numerical recipes in C++*. (Cambridge University Press, 2007).

Supplementary Fig. 13

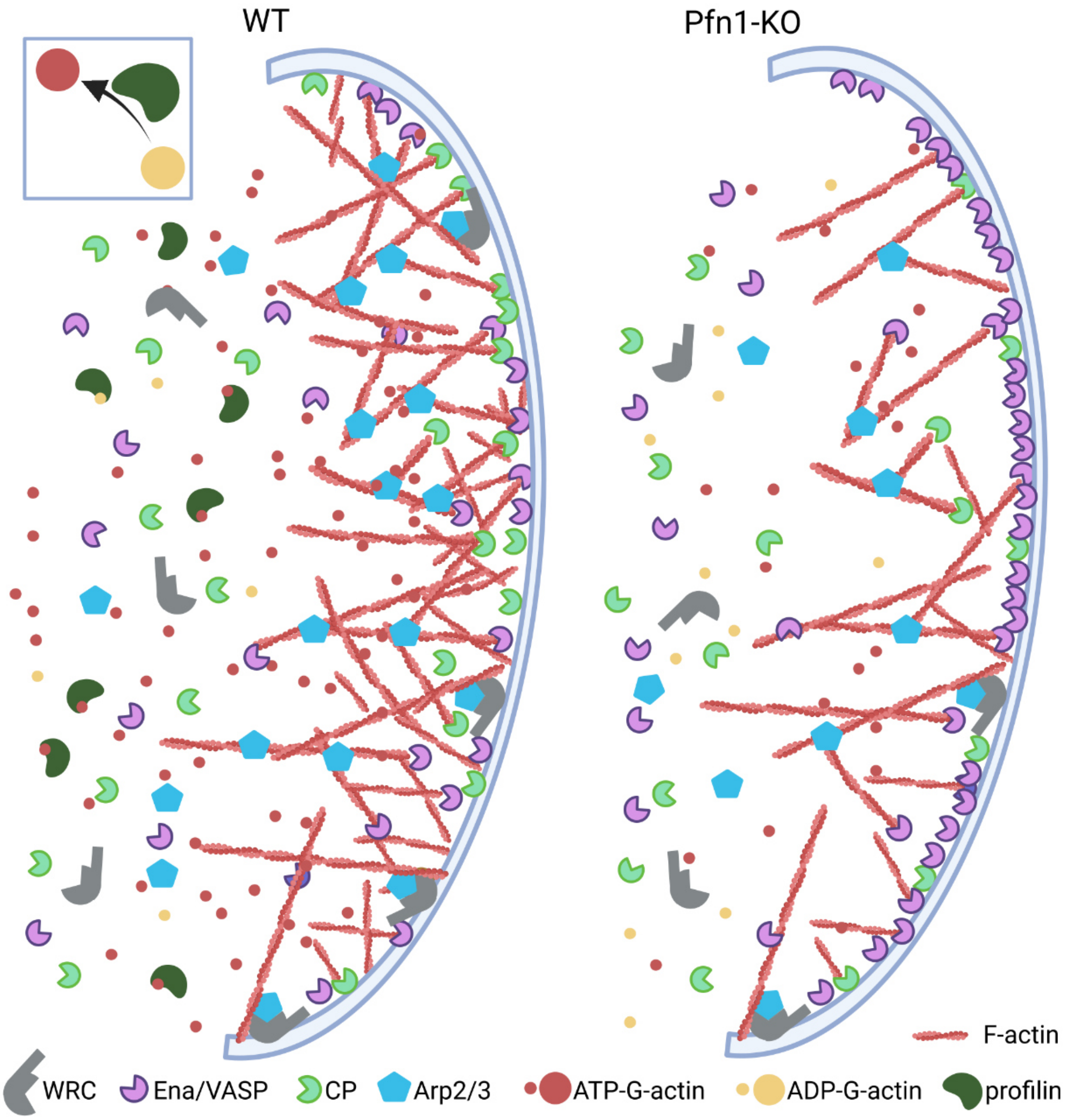

Supplementary Fig. 13. Summary of morphological and molecular transformations caused by Pfn-1 removal. Profilin loss of function reduces total actin levels reflected by both reduced polymerizable actin monomer and lamellipodial actin filaments and coinciding with strongly suppressed, but not eliminated, WRC-dependent Arp2/3 complex incorporation. In contrast, these transformations effect enhanced accumulation at lamellipodial edges of Ena/VASP family members and, as a consequence, reduced CP accumulation. This hypothesis for effects on the actin core machinery upon profilin loss of function is recapitulated by mathematical modeling (see Figs. 7 and S12). Inset on top left emphasizes nucleotide exchange on actin by profilin, symbols for actin regulators are depicted at the bottom and not drawn to scale.

Supplementary Fig. 14

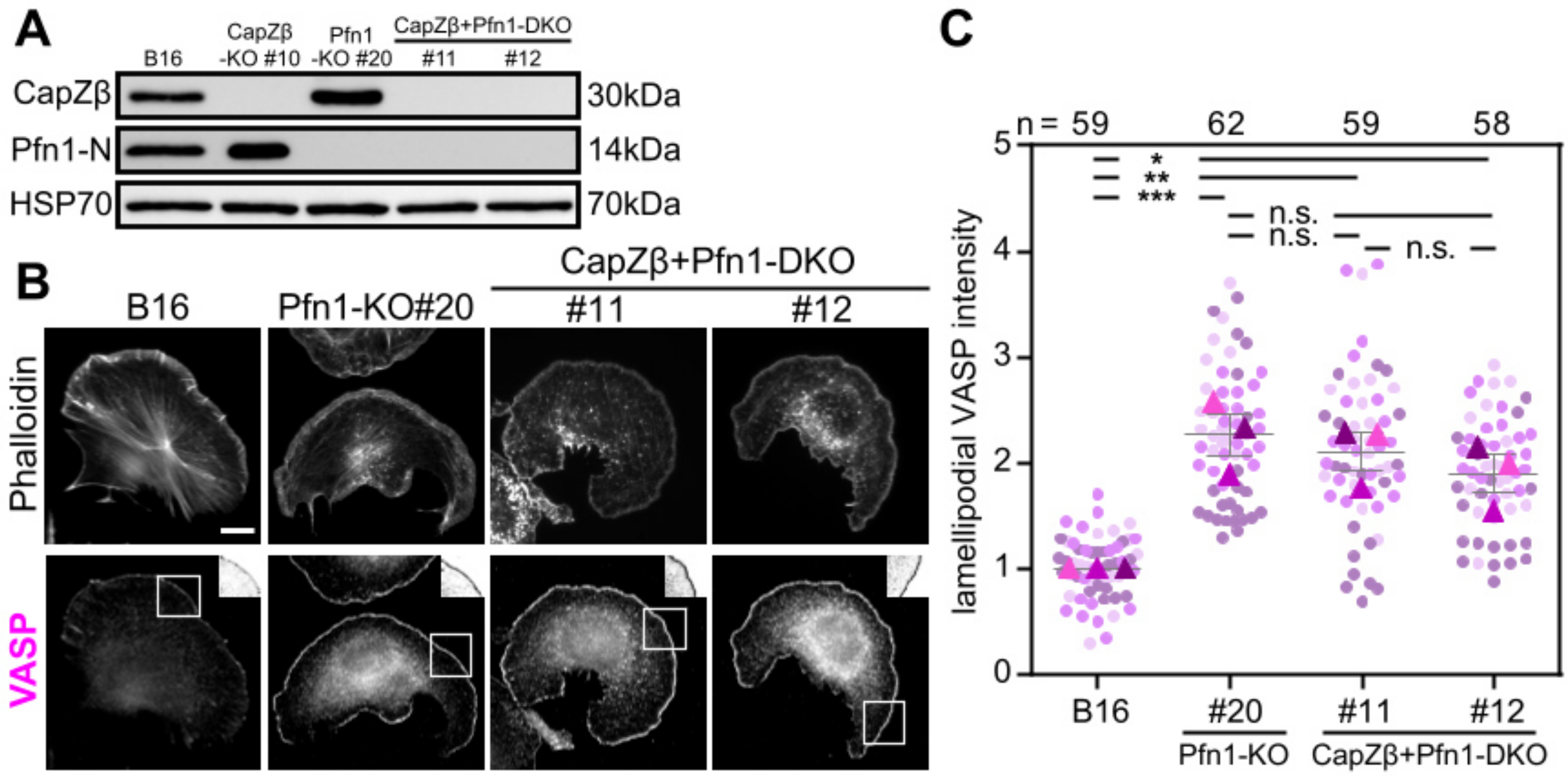

Supplementary Fig. 14. Experimental confirmation of the consequences of combined capping protein and profilin1 disruption on the lamellipodial accumulation of VASP. (**A**) Representative Western blot confirming elimination of expression of CapZβ, Pfn1 or both in respective, newly-generated cell lines, as indicated (CapZβ+Pfn1-DKO #11 and #12). HSP70 was used as loading control. (**B**) Representative phalloidin and VASP stainings of cells of different genotypes, as indicated. White squares mark cellular positions of inverted insets positioned in each subpanel at the top right. Note that the DKOs still harbor a peripheral structure highly reminiscent of a lamellipodium, accompanied by a strong increase of VASP accumulation at their edges, but no sign of additive VASP accumulation in the DKOs as compared to the Pfn1 single KO alone. (**C**) Quantitation of VASP intensity in cells of distinct genotype, as indicated. All parameters of the side-by-side quantitation were as described above, with data again displayed as superplots and arithmetic means of respective experiments shown as colored rectangles from three independent experiments. Statistics: comparison of arithmetic means of replicates of datasets with each other using ANOVA with Tukey's posthoc test, n.s. not significant; *$p \leq 0.05$; **$p \leq 0.01$; ***$p \leq 0.005$.

Uncropped scans of all blots and gels in Supplement

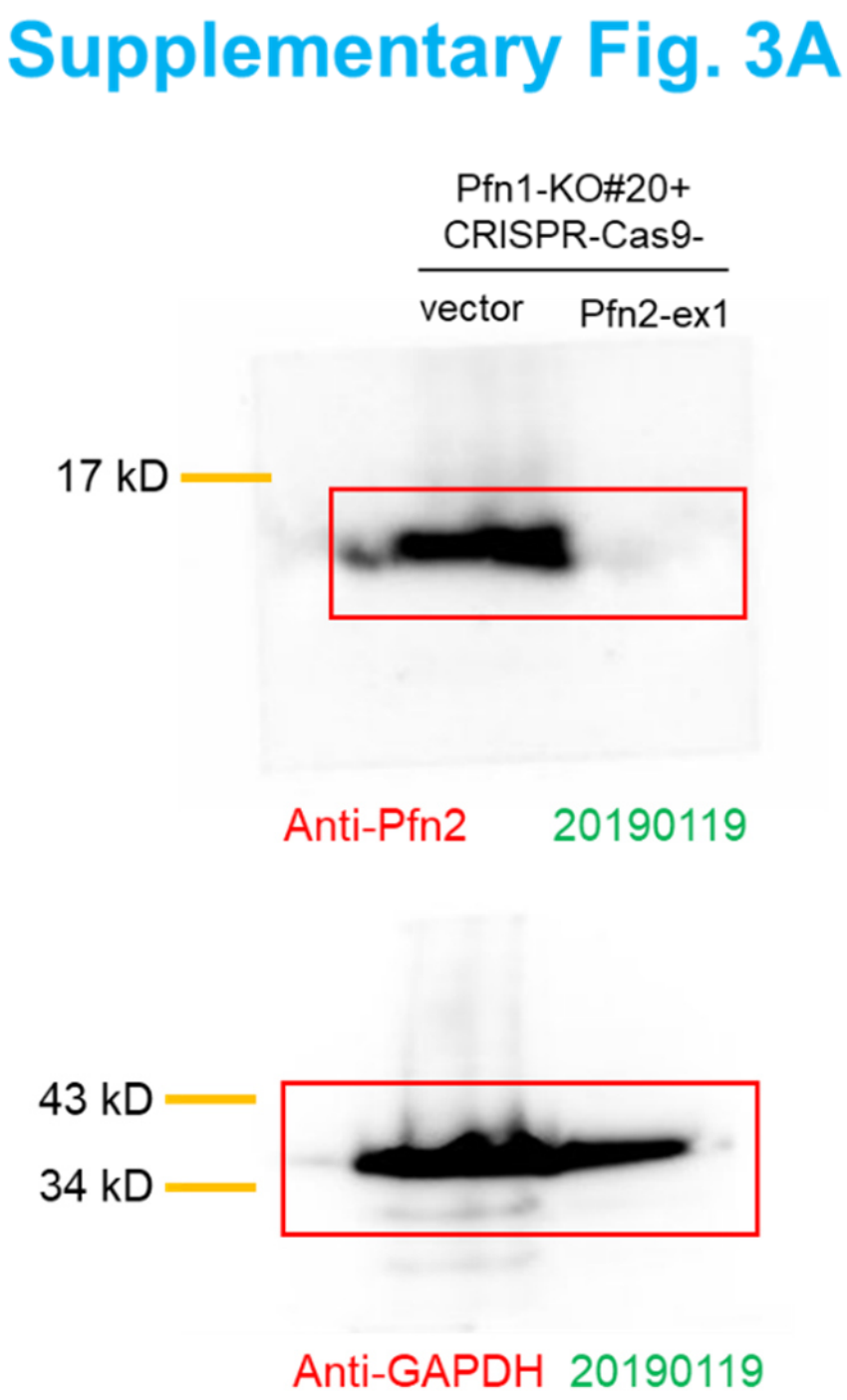

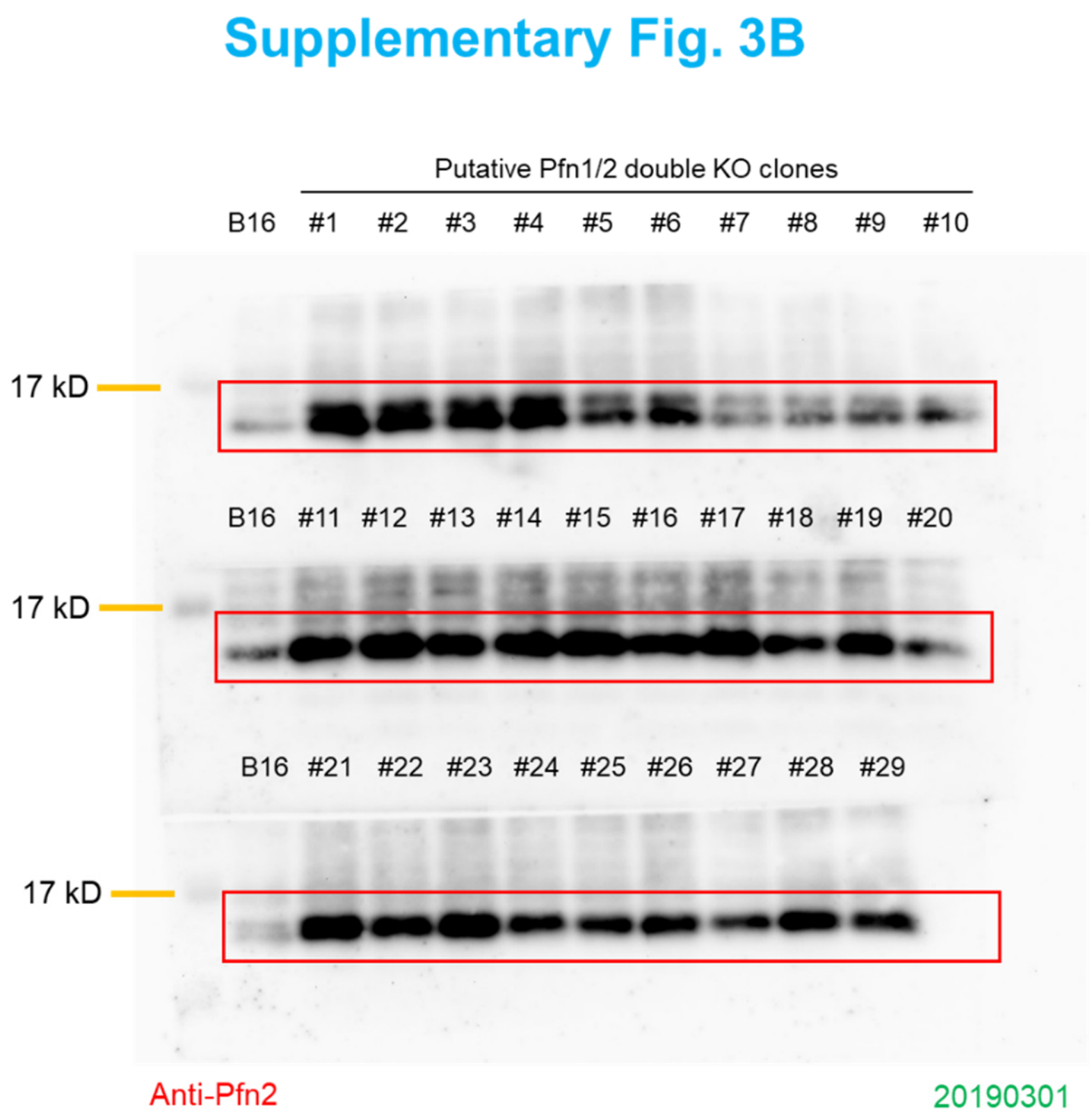

Supplementary Fig. 3C

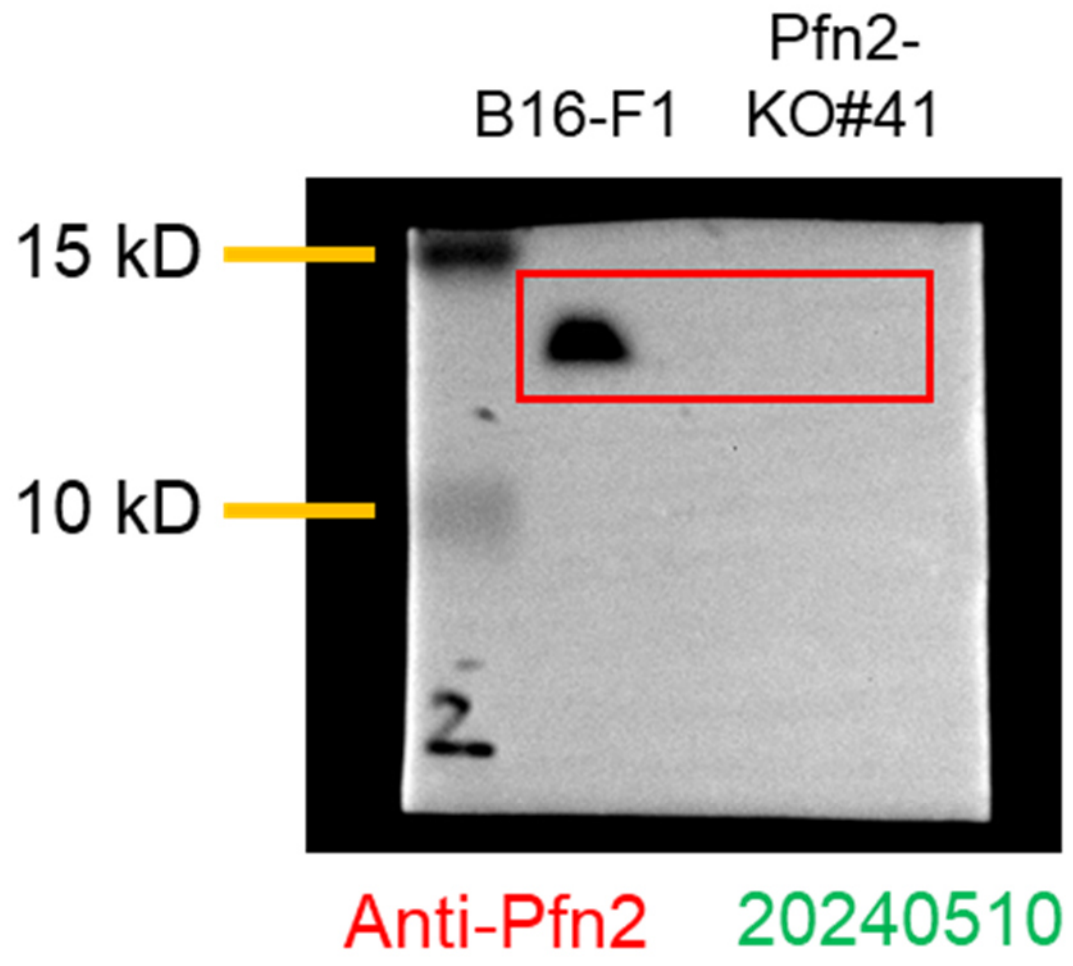

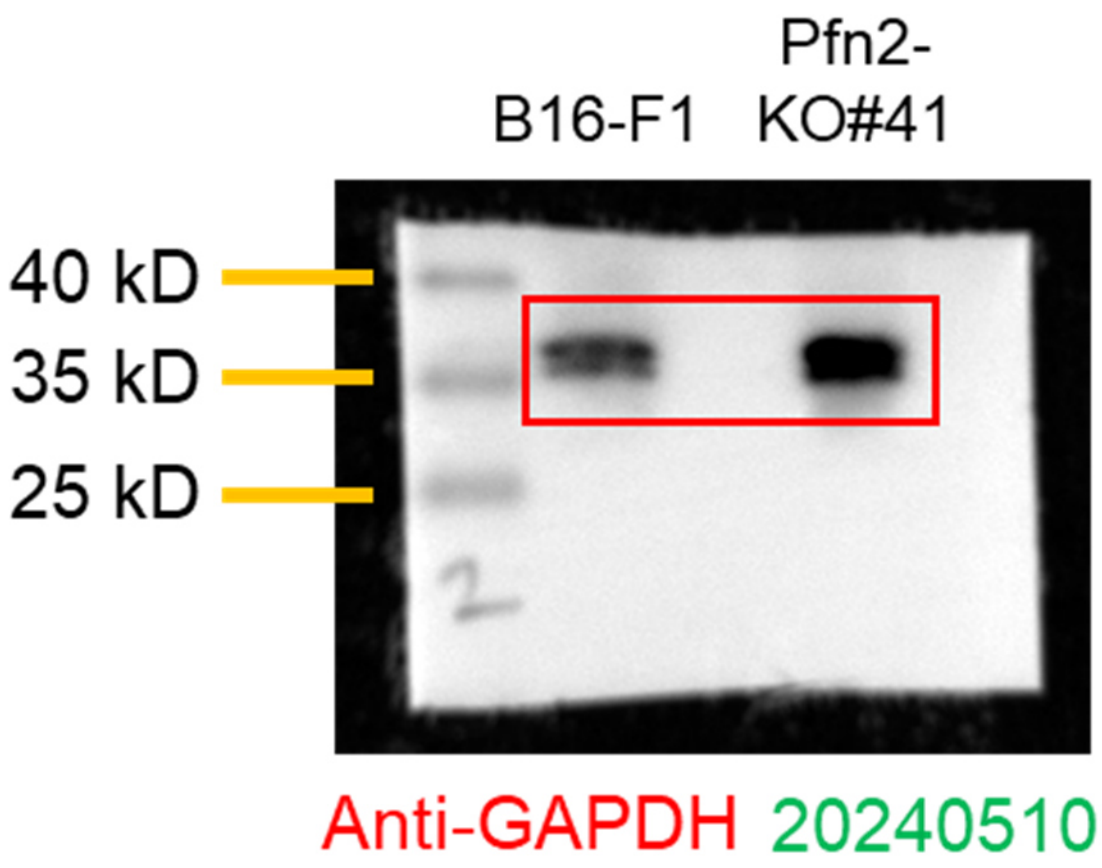

Supplementary Fig. 3D

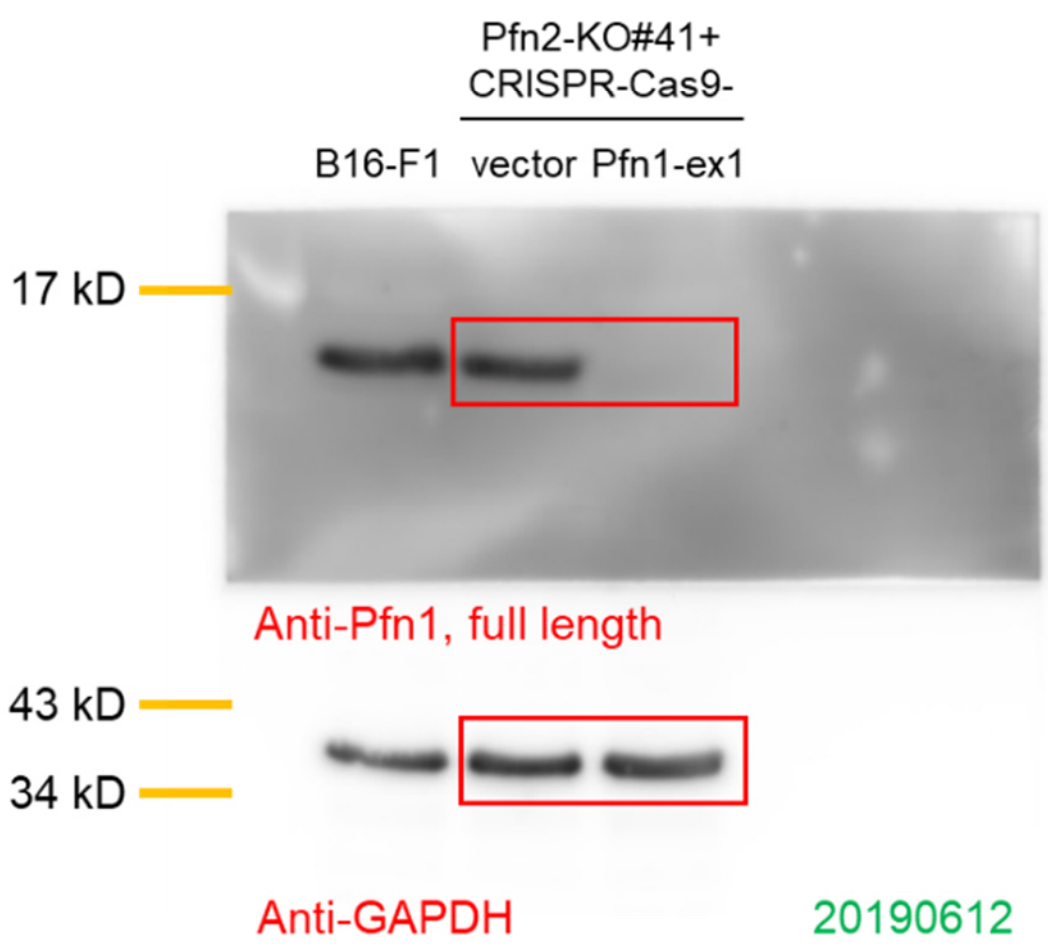

Supplementary Fig. 3E

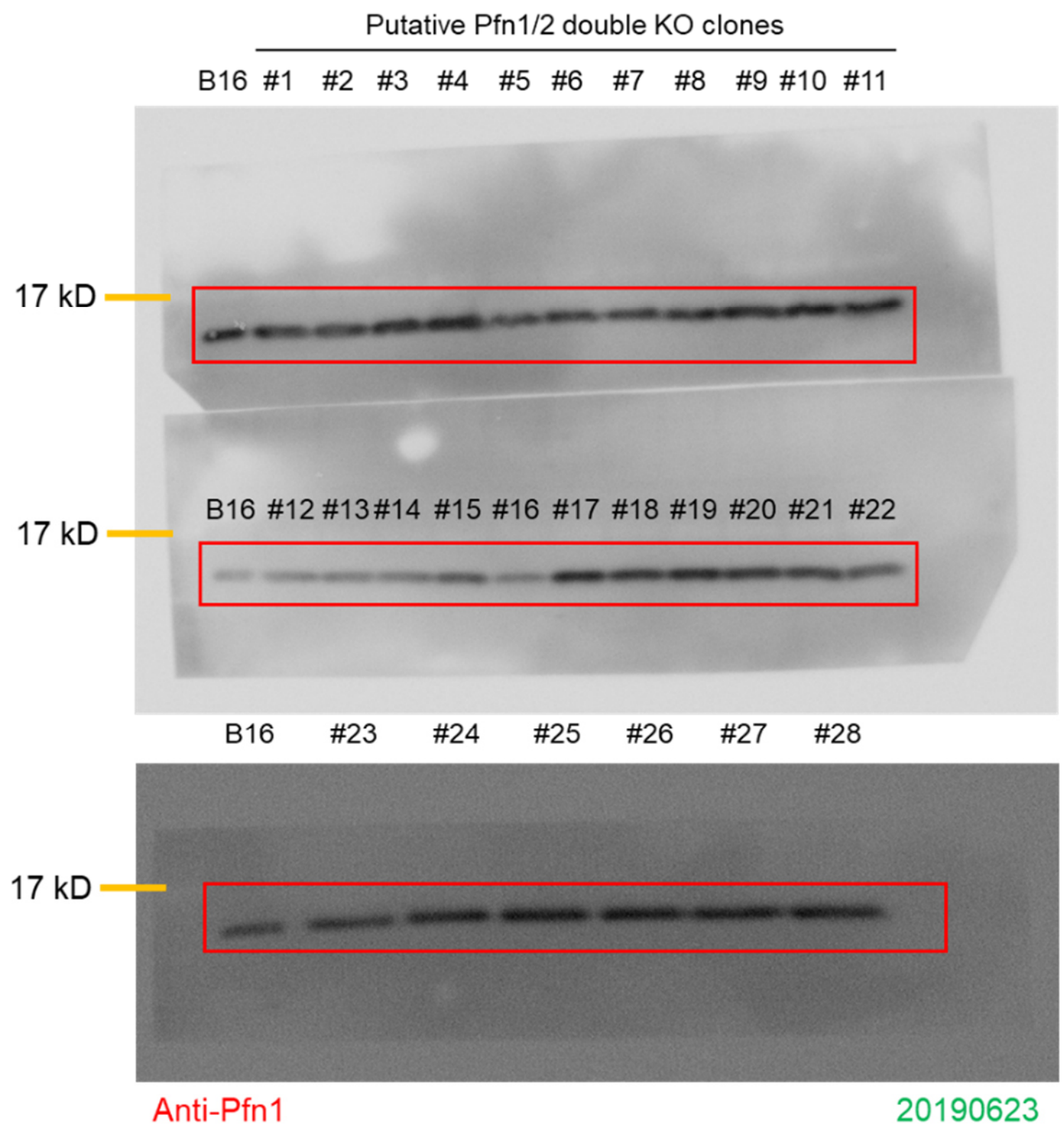

Supplementary Fig. 8A

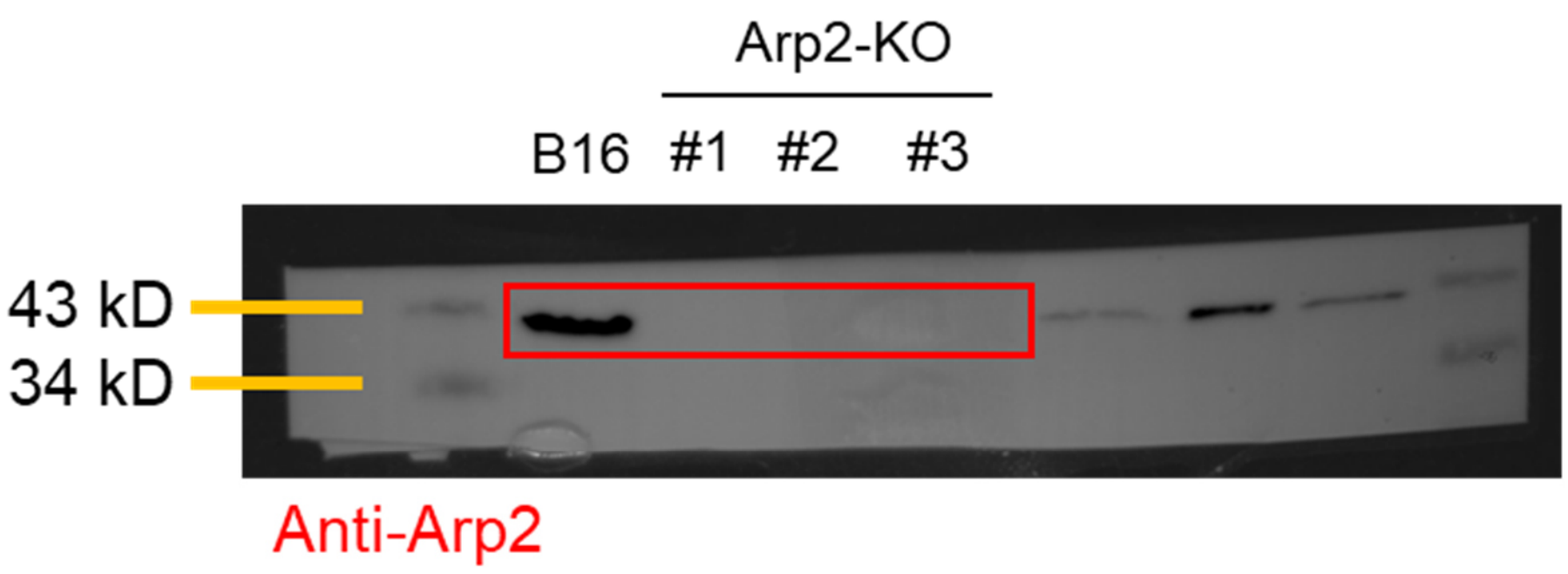

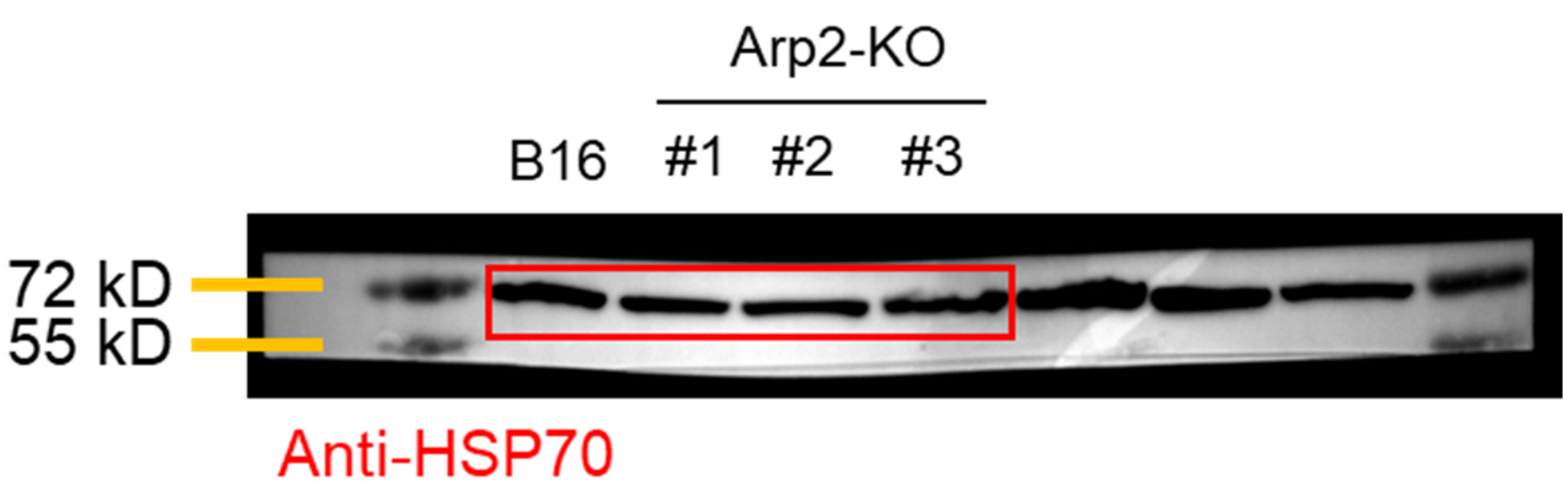

Supplementary Fig. 8E

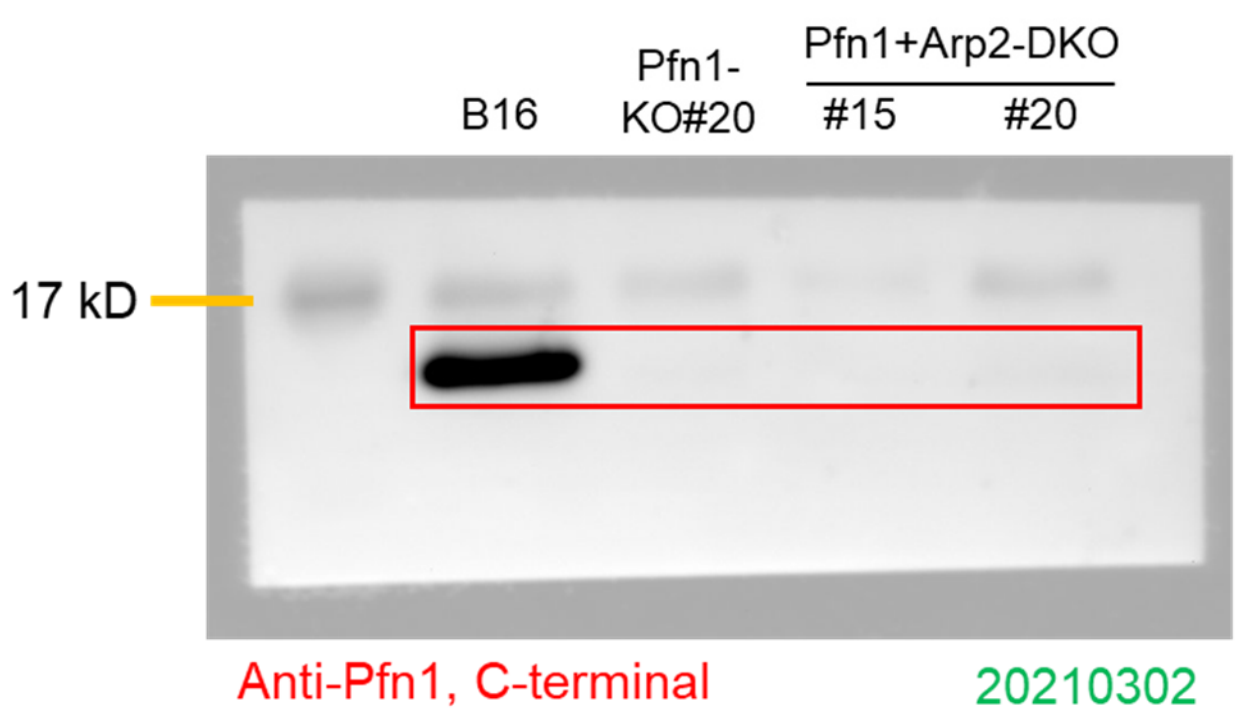

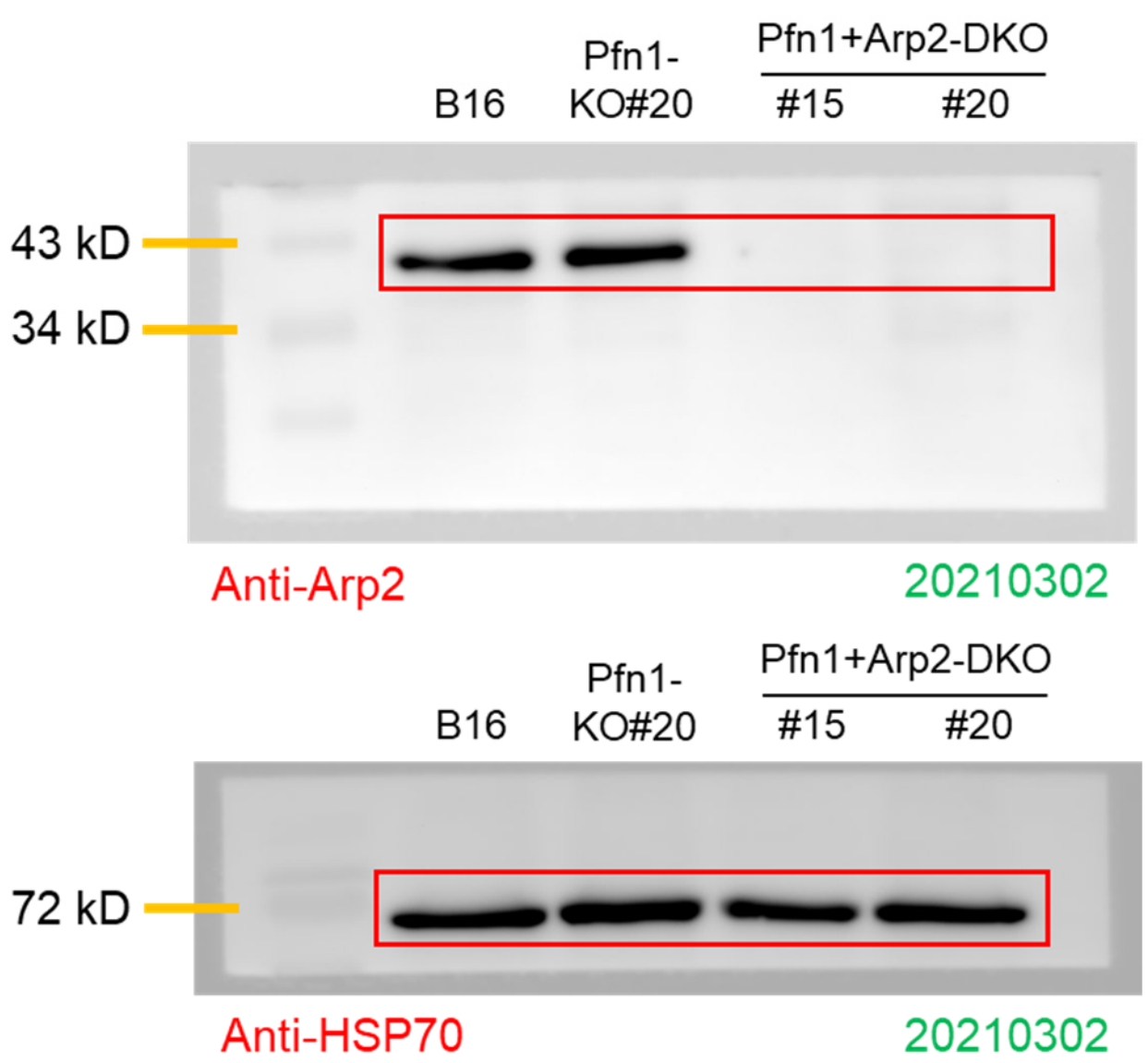

Supplementary Fig. 14A

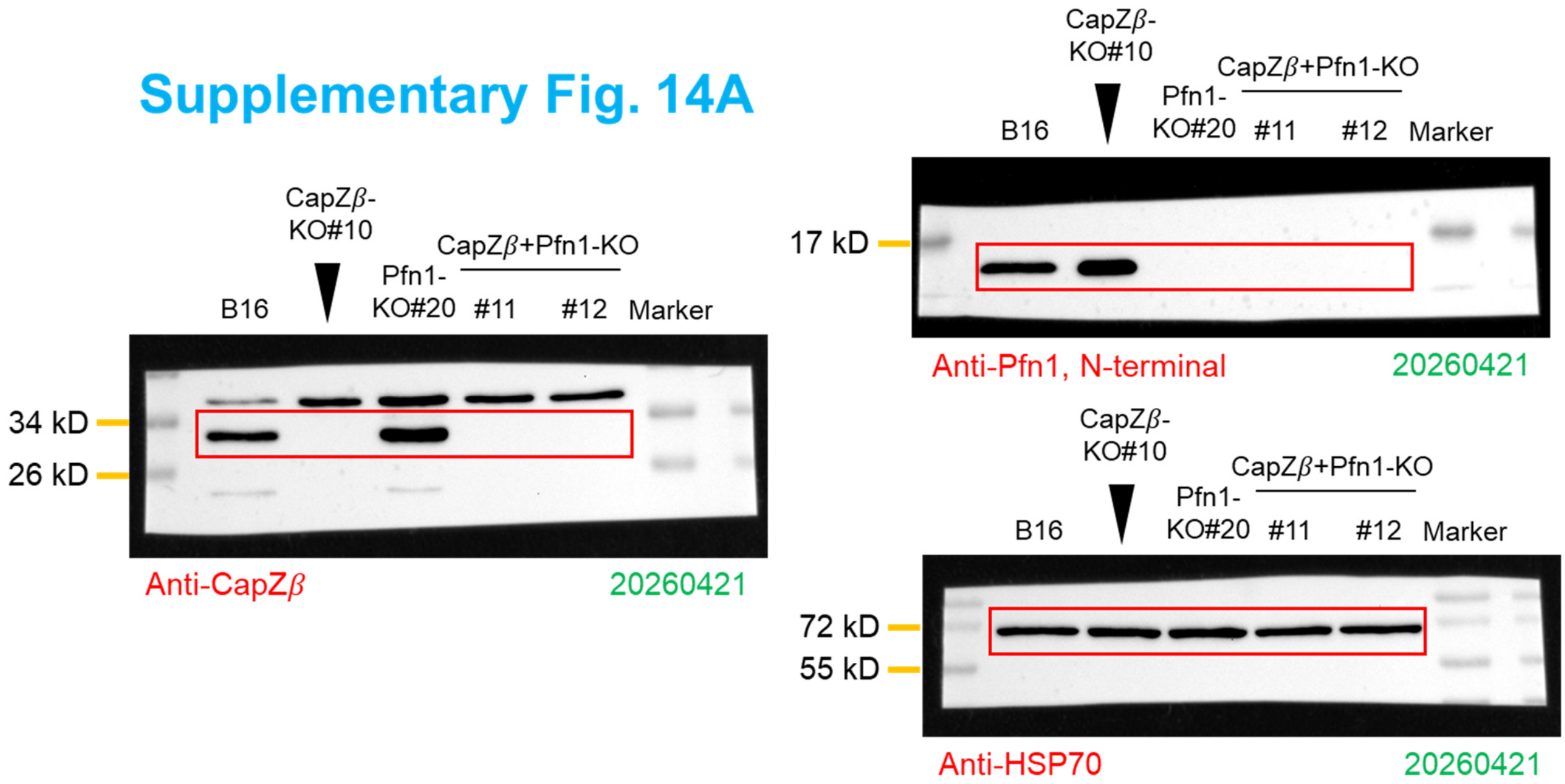

Supplement: Supplementary file 3 [file 41467_2026_77694_MOESM1_ESM.pdf]
