# Supplementary material for: Profilin promotes lamellipodium protrusion by tuning the antagonistic activities of capping protein and VASP
Source: Nat Commun. 2026 Sep 16;17:9887. doi: 10.1038/s41467-026-77694-8 (PMC13582894; doi:10.1038/s41467-026-77694-8)
Supplement: Supplementary file 4 [file 41467_2026_77694_MOESM2_ESM.pdf]

Description of Additional Supplementary Files

Supplementary Movie 1. Potent filopodia induction by active mDia2 requires profilin. Time-lapse phase contrast (phase) and fluorescence microscopy, as indicated, of B16-F1 cells transiently transfected with EGFP-tagged, full length mDia2 (EGFP-mDia2-FL) or a constitutively active variant of the formin (EGFP-mDia2-ΔDAD) known to potently stimulate filopodia formation (see e.g. PMID 18755006). As opposed to full length mDia2, which remains inactive and cytosolic in both the presence and absence of Pfn1, EGFP-mDia2-ΔDAD induces dozens if not hundreds of filopodia or filopodia-like structures in the presence, but not absence of Pfn1. Interestingly, cells lacking Pfn1 are capable of prominent mDia2-ΔDAD positioning at the plasma membrane, but emanation of filopodial bundles appears abrogated in these conditions.

Supplementary Movie 2. Genetic disruption of profilin 1 reduces random cell migration. Time-lapse phase contrast microscopy of B16-F1 wildtype (left) compared to three independently generated Pfn1-KO cell lines, as indicated. Movie was recorded over a time period of 10 hours.

Supplementary Movie 3. Pfn1 gene disruption does not eliminate lamellipodia protrusion. High magnification time-lapse phase contrast microscopy of representative cells in the presence and absence of profilin 1, revealing protrusion of smooth, convex lamellipodia in cells of both genotypes. Note that Pfn1-deficient lamellipodia appear to protrude more slowly as compared to wildtype cell lamellipodia and to be largely devoid of embedded microspike bundles.

Supplementary Movie 4. Pfn1-deficient lamellipodia are capable of actin filament dynamics and turnover. High magnification time-lapse phase contrast and fluorescence microscopy of representative cells harboring (left panel) or lacking Pfn1, as indicated, and transiently transfected with EGFP-actin followed by seeding on laminin. Note the protrusion of smooth lamellipodial edges in Pfn1-deficient cells lines, which consistently appeared largely devoid of F-actin-rich microspike bundles.

Supplementary Movie 5. Pfn1-deficient lamellipodia display reduced, but not abrogated actin network assembly. Video microscopy of FRAP of EGFP-tagged actin transiently expressed in wildtype (B16-F1) versus Pfn1-deficient clones (KO#6, #10, #20). Note the somewhat reduced actin network assembly rate in cell lines lacking Pfn1 as compared to control (left panel).

Supplementary Movie 6. Lamellipodia can incorporate low levels of Arp2/3 complex in the virtual absence of profilin. Pfn1-deficient cells (clone #20) and WT controls (left) were transiently transfected with EGFP-tagged ArpC5B (also known as ArpC5L), one of two variants of the smallest subunits of Arp2/3 complex, formerly known as p16.

Supplementary Movie 7. Genetic removal of Arp2/3 complex in the virtual absence of profilin abrogates random cell migration in B16-F1 cells. Arp2/3 complex-deficient cells are known to be capable, in principle, of migration in B16-F1 melanoma and other mesenchymal cell models, albeit with significantly reduced efficiency as compared to WT (see also our complementary data shown in Fig. S8A-D). In the absence of Pfn1 and thus abrogated formin-dependent actin filament elongation, additional removal of Arp2 was even more detrimental to the efficiency of cell migration in the two Pfn1+Arp2-KO cell lines analyzed (see right panels). This may well be explained by the removal of lamellipodia due to the absence of Arp2/3 complex activity, but even enhanced in phenotype due to the combined elimination of both proper formin and Arp2/3 complex activities.

Supplementary Movie 8. Ena/VASP and profilin contribute to the efficiency of random cell migration in a highly non-redundant fashion. Comparative time-lapse phase contrast microscopy of distinct cell lines, as indicated. Ena/VASP family- and Pfn1-KO cell lines display at best moderate effects on the efficiency of random cell migration, but the combination of these knockouts (EVM+Pfn1-KO) causes severe defects in migration, comparable perhaps in extent to the combined elimination of Pfn1 and Arp2 (see above).

Supplementary Movie 9. Rescue of mDia2-ΔDAD-induced filopodia formation in pfn1-deficient cells with Halo-tagged mPfn1 wildtype, but not with polyproline or actin binding-deficient variants. Pfn1-KO #20 cells were transfected with EGFP-mDia2-ΔDAD, which beautifully accumulates at lamellipodial edges due to being largely incapable of inducing filopodia-like structures in the absence of endogenous Pfn1 and minute amounts of Pfn2 (left panel and Fig. 1). Upon co-expression of Halo-tagged, murine profilin1 as wildtype version (Halo-mPfn1-WT), however, explosive formation of filopodia-like structures tipped by the formin is induced, an effect strongly abrogated again with single point mutants thought to abolish polyproline-binding (Y6D, second panel from the right) or actin-binding (R88E, right panel). For the sake of clarity, Halo-tag expression is not displayed here, but respective, transfected cells are marked with a turquoise plus in each case. Time is as indicated, scale bars: 20 µm each.
